# A Likelihood Ratio Approach for Utilizing Case-Control Data in the Clinical Classification of Rare Sequence Variants: Application to *BRCA1* and *BRCA2*

**DOI:** 10.1155/2023/9961341

**Published:** 2023-09-14

**Authors:** Maria Zanti, Denise G. O'Mahony, Michael T. Parsons, Hongyan Li, Joe Dennis, Kristiina Aittomäkkiki, Irene L. Andrulis, Hoda Anton-Culver, Kristan J. Aronson, Annelie Augustinsson, Heiko Becher, Stig E. Bojesen, Manjeet K. Bolla, Hermann Brenner, Melissa A. Brown, Saundra S. Buys, Federico Canzian, Sandrine M. Caputo, Jose E. Castelao, Jenny Chang-Claude, GC-HBOC study Collaborators, Kamila Czene, Mary B. Daly, Arcangela De Nicolo, Peter Devilee, Thilo Dörk, Alison M. Dunning, Miriam Dwek, Diana M. Eccles, Christoph Engel, D. Gareth Evans, Peter A. Fasching, Manuela Gago-Dominguez, Montserrat García-Closas, José A. García-Sáenz, Aleksandra Gentry-Maharaj, Willemina R. R. Geurts - Giele, Graham G. Giles, Gord Glendon, Mark S. Goldberg, Encarna B. Gómez Garcia, Melanie Güendert, Pascal Guénel, Eric Hahnen, Christopher A. Haiman, Per Hall, Ute Hamann, Elaine F. Harkness, Frans B. L. Hogervorst, Antoinette Hollestelle, Reiner Hoppe, John L. Hopper, Claude Houdayer, Richard S. Houlston, Anthony Howell, ABCTB Investigators, Milena Jakimovska, Anna Jakubowska, Helena Jernström, Esther M. John, Rudolf Kaaks, Cari M. Kitahara, Stella Koutros, Peter Kraft, Vessela N. Kristensen, James V. Lacey, Diether Lambrechts, Melanie Léoné, Annika Lindblom, Jan Lubiński, Michael Lush, Arto Mannermaa, Mehdi Manoochehri, Siranoush Manoukian, Sara Margolin, Maria Elena Martinez, Usha Menon, Roger L. Milne, Alvaro N. Monteiro, Rachel A. Murphy, Susan L. Neuhausen, Heli Nevanlinna, William G. Newman, Kenneth Offit, Sue K. Park, Paul James, Paolo Peterlongo, Julian Peto, Dijana Plaseska-Karanfilska, Kevin Punie, Paolo Radice, Muhammad U. Rashid, Gad Rennert, Atocha Romero, Efraim H. Rosenberg, Emmanouil Saloustros, Dale P. Sandler, Marjanka K. Schmidt, Rita K. Schmutzler, Xiao-Ou Shu, Jacques Simard, Melissa C. Southey, Jennifer Stone, Dominique Stoppa-Lyonnet, Rulla M. Tamimi, William J. Tapper, Jack A. Taylor, Soo Hwang Teo, Lauren R. Teras, Mary Beth Terry, Mads Thomassen, Melissa A. Troester, Celine M. Vachon, Ana Vega, Maaike P. G. Vreeswijk, Qin Wang, Barbara Wappenschmidt, Clarice R. Weinberg, Alicja Wolk, Wei Zheng, Bingjian Feng, Fergus J. Couch, Amanda B. Spurdle, Douglas F. Easton, David E. Goldgar, Kyriaki Michailidou

**Affiliations:** ^1^Biostatistics Unit, The Cyprus Institute of Neurology and Genetics, Nicosia, Cyprus; ^2^Population Health Program, QIMR Berghofer Medical Research Institute, Brisbane, Queensland, Australia; ^3^Cancer Control and Population Science, Huntsman Cancer Institute, University of Utah, Salt Lake City, UT, USA; ^4^Centre for Cancer Genetic Epidemiology, Department of Public Health and Primary Care, University of Cambridge, Cambridge, UK; ^5^Department of Clinical Genetics, Helsinki University Hospital, University of Helsinki, Helsinki, Finland; ^6^Fred A. Litwin Center for Cancer Genetics, Lunenfeld-Tanenbaum Research Institute of Mount Sinai Hospital, Toronto, Ontario, Canada; ^7^Department of Molecular Genetics, University of Toronto, Toronto, Ontario, Canada; ^8^Department of Medicine, Genetic Epidemiology Research Institute, University of California Irvine, Irvine, CA, USA; ^9^Department of Public Health Sciences, and Cancer Research Institute, Queen's University, Kingston, ON, Canada; ^10^Oncology, Department of Clinical Sciences in Lund, Lund University, Lund, Sweden; ^11^Institute for Medical Biometry and Epidemiology, University Medical Center Hamburg-Eppendorf, Hamburg, Germany; ^12^Copenhagen General Population Study, Herlev and Gentofte Hospital, Copenhagen University Hospital, Herlev, Denmark; ^13^Department of Clinical Biochemistry, Herlev and Gentofte Hospital, Copenhagen University Hospital, Herlev, Denmark; ^14^Faculty of Health and Medical Sciences, University of Copenhagen, Copenhagen, Denmark; ^15^Division of Clinical Epidemiology and Aging Research, German Cancer Research Center (DKFZ), Heidelberg, Germany; ^16^Division of Preventive Oncology, German Cancer Research Center (DKFZ) and National Center for Tumor Diseases (NCT), Heidelberg, Germany; ^17^German Cancer Consortium (DKTK), German Cancer Research Center (DKFZ), Heidelberg, Germany; ^18^School of Chemistry and Molecular Biosciences, University of Queensland, Brisbane, Queensland, Australia; ^19^Department of Internal Medicine and Huntsman Cancer Institute, University of Utah, Salt Lake City, UT, USA; ^20^Genomic Epidemiology Group, German Cancer Research Center (DKFZ), Heidelberg, Germany; ^21^Service de Génétique, Institut Curie, Paris, France; ^22^Paris Sciences Lettres Research University, Paris, France; ^23^Oncology and Genetics Unit, Instituto de Investigación Sanitaria Galicia Sur (IISGS), Xerencia de Xestion Integrada de Vigo-SERGAS, Vigo, Spain; ^24^Division of Cancer Epidemiology, German Cancer Research Center (DKFZ), Heidelberg, Germany; ^25^Cancer Epidemiology Group, University Cancer Center Hamburg (UCCH), University Medical Center Hamburg-Eppendorf, Hamburg, Germany; ^26^Center for Familial Breast and Ovarian Cancer, Faculty of Medicine and University Hospital Cologne, University of Cologne, Cologne, Germany; ^27^Department of Medical Epidemiology and Biostatistics, Karolinska Institutet, Stockholm, Sweden; ^28^Department of Clinical Genetics, Fox Chase Cancer Center, Philadelphia, PA, USA; ^29^Center for Omics Sciences, IRCCS San Raffaele Scientific Institute, Milan, Italy; ^30^Department of Pathology, Leiden University Medical Center, Leiden, Netherlands; ^31^Department of Human Genetics, Leiden University Medical Center, Leiden, Netherlands; ^32^Gynaecology Research Unit, Hannover Medical School, Hannover, Germany; ^33^Centre for Cancer Genetic Epidemiology, Department of Oncology, University of Cambridge, Cambridge, UK; ^34^School of Life Sciences, University of Westminster, London, UK; ^35^Faculty of Medicine, University of Southampton, Southampton, UK; ^36^Institute for Medical Informatics, Statistics and Epidemiology, University of Leipzig, Leipzig, Germany; ^37^LIFE-Leipzig Research Centre for Civilization Diseases, University of Leipzig, Leipzig, Germany; ^38^Division of Evolution and Genomic Sciences, School of Biological Sciences, Faculty of Biology, Medicine and Health, University of Manchester, Manchester Academic Health Science Centre, Manchester, UK; ^39^North West Genomics Laboratory Hub, Manchester Centre for Genomic Medicine, St Mary's Hospital, Manchester University NHS Foundation Trust, Manchester Academic Health Science Centre, Manchester, UK; ^40^Department of Gynecology and Obstetrics, Comprehensive Cancer Center Erlangen-EMN, Friedrich-Alexander University Erlangen-Nuremberg, University Hospital Erlangen, Erlangen, Germany; ^41^Genomic Medicine Group, International Cancer Genetics and Epidemiology Group, Fundación Pública Galega de Medicina Xenómica, Instituto de Investigación Sanitaria de Santiago de Compostela (IDIS), Complejo Hospitalario Universitario de Santiago, SERGAS, Santiago de Compostela, Spain; ^42^Division of Cancer Epidemiology and Genetics, National Cancer Institute, National Institutes of Health, Department of Health and Human Services, Bethesda, MD, USA; ^43^Medical Oncology Department, Hospital Clínico San Carlos, Instituto de Investigación Sanitaria San Carlos (IdISSC), Centro Investigación Biomédica en Red de Cáncer (CIBERONC), Madrid, Spain; ^44^MRC Clinical Trials Unit, Institute of Clinical Trials & Methodology, University College London, London, UK; ^45^Department of Clinical Genetics, Erasmus University Medical Center, Rotterdam, Netherlands; ^46^Cancer Epidemiology Division, Cancer Council Victoria, Melbourne, Victoria, Australia; ^47^Centre for Epidemiology and Biostatistics, Melbourne School of Population and Global Health, The University of Melbourne, Melbourne, Victoria, Australia; ^48^Precision Medicine, School of Clinical Sciences at Monash Health, Monash University, Clayton, Victoria, Australia; ^49^Department of Medicine, McGill University, Montréal, QC, Canada; ^50^Division of Clinical Epidemiology, Royal Victoria Hospital, McGill University, Montréal, QC, Canada; ^51^Department of Clinical Genetics, Maastricht University Medical Center, Maastricht, Netherlands; ^52^Molecular Epidemiology Group, C080, German Cancer Research Center (DKFZ), Heidelberg, Germany; ^53^Molecular Biology of Breast Cancer, University Womens Clinic Heidelberg, University of Heidelberg, Heidelberg, Germany; ^54^Institute of Diabetes Research, Helmholtz Zentrum München, German Research Center for Environmental Health, Neuherberg, Germany; ^55^Team 'Exposome and Heredity', CESP, Gustave Roussy, INSERM, University Paris-Saclay, UVSQ, Villejuif, France; ^56^Center for Integrated Oncology (CIO), Faculty of Medicine and University Hospital Cologne, University of Cologne, Cologne, Germany; ^57^Department of Preventive Medicine, Keck School of Medicine, University of Southern California, Los Angeles, CA, USA; ^58^Department of Oncology, Södersjukhuset, Stockholm, Sweden; ^59^Molecular Genetics of Breast Cancer, German Cancer Research Center (DKFZ), Heidelberg, Germany; ^60^Division of Informatics, Imaging and Data Sciences, Faculty of Biology, Medicine and Health, University of Manchester, Manchester Academic Health Science Centre, Manchester, UK; ^61^Nightingale & Genesis Prevention Centre, Wythenshawe Hospital, Manchester University NHS Foundation Trust, Manchester, UK; ^62^NIHR Manchester Biomedical Research Unit, Manchester University NHS Foundation Trust, Manchester Academic Health Science Centre, Manchester, UK; ^63^Family Cancer Clinic, The Netherlands Cancer Institute-Antoni van Leeuwenhoek hospital, Amsterdam, Netherlands; ^64^Department of Medical Oncology, Erasmus MC Cancer Institute, Rotterdam, Netherlands; ^65^Dr. Margarete Fischer-Bosch-Institute of Clinical Pharmacology, Stuttgart, Germany; ^66^University of Tübingen, Tübingen, Germany; ^67^Department of Genetics, F76000 and Normandy University, UNIROUEN, Inserm U1245, Normandy Centre for Genomic and Personalized Medicine, Rouen University Hospital, Rouen, France; ^68^Division of Genetics and Epidemiology, The Institute of Cancer Research, London, UK; ^69^Division of Cancer Sciences, University of Manchester, Manchester, UK; ^70^Australian Breast Cancer Tissue Bank, Westmead Institute for Medical Research, University of Sydney, Sydney, New South Wales, Australia; ^71^Research Centre for Genetic Engineering and Biotechnology 'Georgi D. Efremov', MASA, Skopje, North Macedonia; ^72^Department of Genetics and Pathology, Pomeranian Medical University, Szczecin, Poland; ^73^Independent Laboratory of Molecular Biology and Genetic Diagnostics, Pomeranian Medical University, Szczecin, Poland; ^74^Department of Epidemiology and Population Health, Stanford University School of Medicine, Stanford, CA, USA; ^75^Department of Medicine, Division of Oncology, Stanford Cancer Institute, Stanford University School of Medicine, Stanford, CA, USA; ^76^Radiation Epidemiology Branch, Division of Cancer Epidemiology and Genetics, National Cancer Institute, Bethesda, MD, USA; ^77^Program in Genetic Epidemiology and Statistical Genetics, Harvard T.H. Chan School of Public Health, Boston, MA, USA; ^78^Department of Epidemiology, Harvard T.H. Chan School of Public Health, Boston, MA, USA; ^79^Department of Medical Genetics, Oslo University Hospital and University of Oslo, Oslo, Norway; ^80^Institute of Clinical Medicine, Faculty of Medicine, University of Oslo, Oslo, Norway; ^81^Department of Computational and Quantitative Medicine, City of Hope, Duarte, CA, USA; ^82^City of Hope Comprehensive Cancer Center, City of Hope, Duarte, CA, USA; ^83^Laboratory for Translational Genetics, Department of Human Genetics, KU Leuven, Leuven, Belgium; ^84^VIB Center for Cancer Biology, VIB, Leuven, Belgium; ^85^Genetic and Cancer Medical Laboratory HCL-CLB, Hospices Civils de Lyon, Bron, France; ^86^Department of Molecular Medicine and Surgery, Karolinska Institutet, Stockholm, Sweden; ^87^Department of Clinical Genetics, Karolinska University Hospital, Stockholm, Sweden; ^88^Translational Cancer Research Area, University of Eastern Finland, Kuopio, Finland; ^89^Institute of Clinical Medicine, Pathology and Forensic Medicine, University of Eastern Finland, Kuopio, Finland; ^90^Biobank of Eastern Finland, Kuopio University Hospital, Kuopio, Finland; ^91^Unit of Medical Genetics, Department of Medical Oncology and Hematology, Fondazione IRCCS Istituto Nazionale dei Tumori di Milano, Milan, Italy; ^92^Department of Clinical Science and Education, Södersjukhuset, Karolinska Institutet, Stockholm, Sweden; ^93^Moores Cancer Center, University of California San Diego, La Jolla, CA, USA; ^94^Herbert Wertheim School of Public Health and Human Longevity Science, University of California San Diego, La Jolla, CA, USA; ^95^Department of Cancer Epidemiology, Moffitt Cancer Center, Tampa, FL, USA; ^96^School of Population and Public Health, University of British Columbia, Vancouver, BC, Canada; ^97^Cancer Control Research, BC Cancer, Vancouver, BC, Canada; ^98^Department of Population Sciences, Beckman Research Institute of City of Hope, Duarte, CA, USA; ^99^Department of Obstetrics and Gynecology, Helsinki University Hospital, University of Helsinki, Helsinki, Finland; ^100^Clinical Genetics Research Lab, Department of Cancer Biology and Genetics, Memorial Sloan Kettering Cancer Center, New York, NY, USA; ^101^Clinical Genetics Service, Department of Medicine, Memorial Sloan Kettering Cancer Center, New York, NY, USA; ^102^Department of Preventive Medicine, Seoul National University College of Medicine, Seoul, Republic of Korea; ^103^Integrated Major in Innovative Medical Science, Seoul National University College of Medicine, Seoul, Republic of Korea; ^104^Cancer Research Institute, Seoul National University, Seoul, Republic of Korea; ^105^Sir Peter MacCallum Department of Oncology, The University of Melbourne, Melbourne, Victoria, Australia; ^106^Parkville Familial Cancer Centre, Peter MacCallum Cancer Centre and Royal Melbourne Hospital, Melbourne, Victoria, Australia; ^107^Genome Diagnostics Program, IFOM-ETS the AIRC Institute of Molecular Oncology, Milan, Italy; ^108^Department of Non-Communicable Disease Epidemiology, London School of Hygiene and Tropical Medicine, London, UK; ^109^Department of General Medical Oncology and Multidisciplinary Breast Centre, Leuven Cancer Institute and University Hospitals Leuven, Leuven, Belgium; ^110^Unit of Molecular Bases of Genetic Risk and Genetic Testing, Department of Research, Fondazione IRCCS Istituto Nazionale dei Tumori (INT), Milan, Italy; ^111^Department of Basic Sciences, Shaukat Khanum Memorial Cancer Hospital and Research Centre (SKMCH & RC), Lahore, Pakistan; ^112^Clalit National Cancer Control Center, Carmel Medical Center and Technion Faculty of Medicine, Haifa, Israel; ^113^Medical Oncology Department, Hospital Universitario Puerta de Hierro, Madrid, Spain; ^114^Department of Pathology, The Netherlands Cancer Institute-Antoni van Leeuwenhoek hospital, Amsterdam, Netherlands; ^115^Department of Oncology, University Hospital of Larissa, Larissa, Greece; ^116^Epidemiology Branch, National Institute of Environmental Health Sciences, NIH, Research Triangle Park, NC, USA; ^117^Division of Molecular Pathology, The Netherlands Cancer Institute, Amsterdam, Netherlands; ^118^Division of Psychosocial Research and Epidemiology, The Netherlands Cancer Institute-Antoni van Leeuwenhoek hospital, Amsterdam, Netherlands; ^119^Department of Clinical Genetics, Leiden University Medical Center, Leiden, Netherlands; ^120^Center for Molecular Medicine Cologne (CMMC), Faculty of Medicine and University Hospital Cologne, University of Cologne, Cologne, Germany; ^121^Division of Epidemiology, Department of Medicine, Vanderbilt Epidemiology Center, Vanderbilt-Ingram Cancer Center, Vanderbilt University School of Medicine, Nashville, TN, USA; ^122^Genomics Center, Centre Hospitalier Universitaire de Québec-Université Laval Research Center, Québec City, QC, Canada; ^123^Department of Clinical Pathology, The University of Melbourne, Melbourne, Victoria, Australia; ^124^Genetic Epidemiology Group, School of Population and Global Health, University of Western Australia, Perth, Western Australia, Australia; ^125^Department of Tumour Biology, INSERM U830, Paris, France; ^126^Université Paris Descartes, Paris, France; ^127^Department of Population Health Sciences, Weill Cornell Medicine, New York, NY, USA; ^128^Epigenetic and Stem Cell Biology Laboratory, National Institute of Environmental Health Sciences, NIH, Research Triangle Park, NC, USA; ^129^Breast Cancer Research Programme, Cancer Research Malaysia, Subang Jaya, Selangor, Malaysia; ^130^Department of Surgery, Faculty of Medicine, University of Malaya, UM Cancer Research Institute, Kuala Lumpur, Malaysia; ^131^Department of Population Science, American Cancer Society, Atlanta, GA, USA; ^132^Department of Epidemiology, Mailman School of Public Health, Columbia University, New York, NY, USA; ^133^Department of Clinical Genetics, Odense University Hospital, Odence C, Denmark; ^134^Department of Epidemiology, Gillings School of Global Public Health and UNC Lineberger Comprehensive Cancer Center, University of North Carolina at Chapel Hill, Chapel Hill, NC, USA; ^135^Department of Quantitative Health Sciences, Division of Epidemiology, Mayo Clinic, Rochester, MN, USA; ^136^Centro de Investigación en Red de Enfermedades Raras (CIBERER), Madrid, Spain; ^137^Fundación Pública Galega de Medicina Xenómica, Santiago de Compostela, Spain; ^138^Instituto de Investigación Sanitaria de Santiago de Compostela (IDIS), Complejo Hospitalario Universitario de Santiago, SERGAS, Santiago de Compostela, Spain; ^139^Biostatistics and Computational Biology Branch, National Institute of Environmental Health Sciences, NIH, Research Triangle Park, NC, USA; ^140^Institute of Environmental Medicine, Karolinska Institutet, Stockholm, Sweden; ^141^Department of Surgical Sciences, Uppsala University, Uppsala, Sweden; ^142^Department of Dermatology, Huntsman Cancer Institute, University of Utah School of Medicine, Salt Lake City, UT, USA; ^143^Department of Laboratory Medicine and Pathology, Mayo Clinic, Rochester, MN, USA

## Abstract

A large number of variants identified through clinical genetic testing in disease susceptibility genes are of uncertain significance (VUS). Following the recommendations of the American College of Medical Genetics and Genomics (ACMG) and Association for Molecular Pathology (AMP), the frequency in case-control datasets (PS4 criterion) can inform their interpretation. We present a novel case-control likelihood ratio-based method that incorporates gene-specific age-related penetrance. We demonstrate the utility of this method in the analysis of simulated and real datasets. In the analysis of simulated data, the likelihood ratio method was more powerful compared to other methods. Likelihood ratios were calculated for a case-control dataset of *BRCA1* and *BRCA2* variants from the Breast Cancer Association Consortium (BCAC) and compared with logistic regression results. A larger number of variants reached evidence in favor of pathogenicity, and a substantial number of variants had evidence against pathogenicity—findings that would not have been reached using other case-control analysis methods. Our novel method provides greater power to classify rare variants compared with classical case-control methods. As an initiative from the ENIGMA Analytical Working Group, we provide user-friendly scripts and preformatted Excel calculators for implementation of the method for rare variants in *BRCA1*, *BRCA2*, and other high-risk genes with known penetrance.

## 1. Introduction

Clinical genetic testing of disease susceptibility genes often identifies variants of uncertain significance (VUS), complicating the clinical management of carriers and their families [1]. The assessment of the clinical significance of these rare sequence variants, including missense substitutions, in-frame deletions and insertions, and intronic variants, is essential to directing the clinical management of carriers and their relatives towards appropriate prevention, early detection, and personalized treatments.

The most widely used method for the interpretation of germline variants is via the application of the standards and guidelines recommended by the American College of Medical Genetics and Genomics and the Association for Molecular Pathology (ACMG/AMP) [2]. Strength levels (very strong, strong, moderate, and supporting) are assigned to independent lines of evidence for or against variant pathogenicity. These strength levels are then combined and used in a scoring system to provide a clinical class, expressed as pathogenic, likely pathogenic, likely benign, benign, or VUS. These guidelines integrate various sources of information including the variant's nature and position (e.g., nonsense, frameshift, and missense) and clinical data (e.g., prevalence in affected individuals and controls), and the combination of this information is interpreted to establish the significance of the variant under investigation with respect to risk. These criteria were recently reinterpreted in a quantitative Bayesian framework, which derived ranges of likelihood ratios (LRs) consistent with each of the evidence strength levels [3]. For case-control data, the specific criterion (PS4) states that a relative risk (RR) or odds ratio (OR) > 5.0 with nominal statistical significance (i.e., the confidence interval of the RR or OR does not include 1) provides strong evidence in favor of pathogenicity [2].

A significant advance in the classification of variants in cancer and other disease genes was the development of the multifactorial integrated likelihood ratio model [4]; this model combines multiple features under the assumption that each of them is an independent predictor of variant pathogenicity in a Bayesian framework, thus providing a quantitative estimate of the pathogenicity of a variant [5]. The ENIGMA consortium [6] has been applying and extending this multifactorial likelihood model. To date, application of this model has included clinically calibrated prior probabilities of pathogenicity derived from bioinformatic prediction of variant effect and location, along with a combined LR derived from clinical data [5], such as family history of cancer [7], breast cancer tumor pathology [8], variant cosegregation with disease [9, 10], and variant cooccurrence in trans with a pathogenic variant (PV) in the same gene [7]. This model can also incorporate LRs derived from variant frequency in cases and controls. Recently, case-control information derived from genotype data for 20 variants was incorporated into a comprehensive multifactorial likelihood analysis of *BRCA1* and *BRCA2* variants by ENIGMA [11], using a method incorporating gene- and age-specific penetrance of PV carriers only. Such case-control LR calculations take into consideration gene- and age-specific penetrance values, and hence they might be expected to outperform the statistical measures currently recommended by ACMG/AMP for the analysis of case-control data (i.e., OR or RR estimates).

In this paper, we present a novel case-control LR method, based on the same principle as used in Parsons et al.'s [11], that incorporates age information in both carriers and noncarriers in the dataset. The method can be used to obtain evidence in favor or against pathogenicity for rare variants in any gene for which there exist known age-specific penetrance estimates based on data obtained from case-control studies. We illustrate the use of this method to calculate LRs for 24 *BRCA1* and 68 *BRCA2* variants from breast cancer case-control genotype data generated by the Breast Cancer Association Consortium (BCAC) as part of the large-scale OncoArray project [12]. We further demonstrate the utility of this case-control LR approach to aid in the interpretation of the clinical significance of variants using evidence aligned to ACMG/AMP code strengths or other classification methods.

## 2. Methods

### 2.1. Case-Control Datasets

#### 2.1.1. Simulated Case-Control Dataset

Genotype data simulations were performed using the R (v3.6.1) (https://www.r-project.org/) statistical computing language. To create case-control datasets, genotypes for cases and controls were simulated using a Poisson distribution with *lambda* (*λ*) equal to the mean number of events (variant carriers) in the given interval, expressed as

(1) $$\begin{array}{c} \lambda_{\text{Cases}}=N\times \text{RR}\times \text{MAF},\\ \lambda_{\text{Controls}}=N\times \text{MAF}, \end{array}$$

where N denotes the sample size, RR denotes the relative breast cancer risk of the causal variant and MAF denotes the minor allele frequency of the variant in the general population. Ages were simulated using a normal distribution, with the mean and standard deviation following the gene-specific age distribution in the CARRIER population-based study [13].

Genotype data simulations were carried out for variants conferring a RR of 1 (indicating no increased risk), 2, 3, 4, 5, 6, 7, 8, 9, or 10, minor allele frequency in controls of 0.0001, 0.00005, or 0.00003, and sample size of *N* = 20,000 (20,000 breast cancer cases and 20,000 controls), 30,000 (30,000 breast cancer cases and 30,000 controls), or 50,000 (50,000 breast cancer cases and 50,000 controls). For each of these 90 scenarios, we simulated 10,000 replicates.

Additionally, in order to account for the possibility that age information is not available, we repeated the analysis using same age for all individuals.

#### 2.1.2. BCAC OncoArray Dataset

Genotype data were generated as part of the BCAC component of the OncoArray project [12] (studies included in the analysis are listed in Supplementary Table S1) and were available for 75,657 breast cancer cases and 52,987 controls of European ancestry. The majority of studies were population-based case–control studies or case–control studies nested within population-based cohorts. However, a subset of studies oversampled cases with a family history of breast cancer. Of these, 464 breast cancer cases and 1,347 controls had missing information regarding their age at diagnosis or interview, respectively and were excluded from the analyses. Another 1,445 cases and 858 controls were removed because their ages fell outside the interval of 21-80 years (the age range for which penetrance estimates were available). Cluster plots of 56 *BRCA1* and 127 *BRCA2* variants, nominated by ENIGMA researchers for inclusion in the OncoArray project were manually checked to review the automated calls. This was performed since automated genotype calling for rare variants from GWAS chips has been shown to be suboptimal [14]. Genotypes were adjusted for 41 *BRCA1* and 91 *BRCA2* variants, while 3 *BRCA1* and 2 *BRCA2* variant genotypes were determined to have been called correctly by automated clustering. Genotype recalling was not performed for 12 *BRCA1* and 34 *BRCA2* variants due to the low quality of the genotype data; these variants were not considered further.

After genotype cluster review and recalling, 16 *BRCA1* and 19 *BRCA2* variants were excluded from further analysis due to their high frequency (>0.1%). Additionally, case-control LR calculations were not possible for four *BRCA1* and six *BRCA2* variants due to the absence of variant carriers in the postfiltering dataset. After these exclusions, case-control LR and logistic regression analyses were performed for 24 *BRCA1* and 68 *BRCA2* variants. It should be noted that some of the variants selected for the array have subsequently been classified or were those whose pathogenicity status were known and were included as positive or negative controls.

### 2.2. Statistical Analyses

#### 2.2.1. Case-Control Likelihood Ratio Method

This method (detailed in Supplementary File 1) compares the likelihood of the distribution of the variant of interest among cases and controls under the hypothesis that the variant is associated with similar risks of the disease in question as the “average” pathogenic variant (*H*_*p*_), compared to the likelihood under the hypothesis that it is a benign variant not associated with increased risk (*H*_*b*_). These risks may be age-, sex-, and/or country-specific. Thus

(2) $$\begin{array}{c} \text{LR}=\frac{\text{Pr }\left(\text{Data}\left|H_{p}\right.\right)\;}{\text{Pr}\left(\text{Data}\left|H_{b}\right.\right)}, \end{array}$$

where Data denotes observed data on carrier status of a variant of interest, case-control status, and age at diagnosis or interview, combined over all individuals in the dataset.

In order to calculate the above LR, we follow a survival analysis framework. We first determine the probability that an individual with genotype *k* remains unaffected at age *t*, *S*_*k*_(*t*), and the corresponding probability that an individual with genotype *k* is affected at age *t*, *f*_*k*_(*t*) (where *k* = 0 or 1 for non-carriers and carriers, respectively). These probabilities can be computed from the age-specific baseline incidence, *λ*_0_(*t*), and the age-specific log-relative risk of an assumed pathogenic variant in the gene of interest, *β*(*t*). These probabilities are given by

(3) $$\begin{array}{c} S_{k}\left(t\right)=\exp\left(-\int_{0}^{t}\lambda_{0}\left(t\right)e^{\beta \left(t\right)k}dt\right),\\ f_{k}\left(t\right)=S_{k}\left(t\right)\;e^{\beta \left(t\right)k}. \end{array}$$

As detailed in Supplementary File 1, the likelihood ratio is to close approximation, given by

(4) $$\begin{array}{c} \text{ccLR}=\frac{\left(\prod_{v_{j}=1}S_{1}\left(t_{j}\right)e^{\beta \left(t\right)d_{j}}/S_{0}\left(t_{j}\right)\right)/\left({\left(\sum_{j}^{N}S_{1}\left(t_{j}\right)e^{\beta \left(t_{j}\right)d_{j}}/S_{0}\left(t_{j}\right)\right)}^{K}\right)}{1/N^{K}}, \end{array}$$

where *N* is the total number of individuals, *K* is the number of variant carriers, *v*_*j*_ is the variant status (0 for noncarriers and 1 for variant carriers), and *d*_*j*_ is the disease status (0 for controls and 1 for cases) for individual *j*.

The baseline incidence rates *λ*_0_(*t*) were taken from the age-specific background rates for England and Wales (1998-2002) (https://ci5.iarc.fr/CI5I-X/Default.aspx), and the age-specific breast cancer relative risks for pathogenic variant carriers *β*(*t*) were taken from the recent large-scale BRIDGES (Breast Cancer Risk after Diagnostic Gene Sequencing) project [15]. To allow for possible carrier frequency differences by country, stratified LR calculations were performed within each country and then multiplied to provide a final LR.

Likelihood ratios are further translated into ACMG/AMP code strength categories according to published recommendations [3]. Likelihood ratio estimates in favor of variant pathogenicity are scored as very strong, LR ≥ 350; strong, 350 > LR ≥ 18.7; moderate, 18.70 > LR ≥ 4.33; and supporting, 4.33 > LR ≥ 2.08. Likelihood ratio evidence for benign variant status is scored as very strong, LR ≤ 0.0029; strong, 0.0029 < LR ≤ 0.053; moderate, 0.053 < LR ≤ 0.231; and supporting, 0.231 < LR ≤ 0.48. No evidence strength corresponded to estimates of 0.48 ≤ LR < 2.08.

In a series of sensitivity analyses, the method was applied using three other published RR estimates: from case series unselected for family history of breast cancer [16], cohort series of *BRCA1* and *BRCA2* carriers [17], and breast cancer hazard ratio estimates for missense *BRCA1* and *BRCA2* variants [18]. In order to account for country-specific effects, the stratified analysis was also performed using age- and country-specific incidence rates derived from the Cancer Incidence in Five Continents, volume 9, 1998-2002, (https://ci5.iarc.fr/CI5I-X/Default.aspx). Age-specific breast cancer incidences for Greece and North Macedonia were retrieved from the 2020 cancer registry (European Cancer Information System (ECIS), https://ecis.jrc.ec.europa.eu/) since cancer incidence data were not available for the years 1998-2019. Unstratified analyses were also performed for comparison.

Detailed R scripts and preformatted Excel calculators (user can either input individual-level data or tabulated by age groups) for the calculation of case-control LRs can be found using the following GitHub link (https://github.com/BiostatUnitCING/ccLR). The files provided can be used to derive estimates based on the RR from Dorling et al. [15], Kuchenbaecker et al. [17], or Antoniou et al. [16]. In addition, this method can also be used to compute case-control LRs for variants in other disease susceptibility genes by using age-specific penetrance estimates for the gene of interest (indicated by “custom” gene in the preformatted Excel calculators and R script). Furthermore, to allow for the possibility that age information is not available (or is only available for a subset of the dataset), the user can incorporate individuals with unknown age at diagnosis or interview into any of the age groups specified in the tabulated calculator.

#### 2.2.2. Odds Ratio Analysis

Odds ratio analysis was performed using logistic regression adjusted by age and country (if applicable) and Fisher's exact test (corrected using Haldane's method when simulations resulted in zero variant carriers in cases or controls [19]). Logistic regression *p* values were estimated using the likelihood ratio test. Based on the original ACMG/AMP recommendations [2], an OR estimate greater than 5.0, with the confidence interval not including 1.0, was used to define strong evidence of pathogenicity (PS4).

#### 2.2.3. Evaluation and Application of the Case-Control Analyses Methods

The simulated datasets were analyzed using the novel case-control LR method, logistic regression (adjusted by age), and Fisher's exact test. The case-control LR method was applied using age-specific breast cancer ORs for *BRCA1* and *BRCA2* PVs [15]. For causal variants with a relative risk of 2 to 10, the power of the case-control LR method was estimated either as the probability of reaching at least supporting (LR ≥ 2.08) or at least strong pathogenic (LR ≥ 18.7) evidence. For benign variants with a relative risk of 1, the power of the case-control LR method was estimated either as the probability of reaching at least supporting (LR ≤ 0.48) or at least strong (LR ≤ 0.053) benign ACMG/AMP evidence. Correspondingly, type I error for pathogenicity was calculated as the probability of obtaining at least supporting or at least strong pathogenic ACMG/AMP evidence when the relative risk was set to 1. Equivalently, type I error for evidence against pathogenicity was calculated as the probability of obtaining at least supporting or at least strong benign ACMG/AMP evidence when the relative risk was greater than one. The power of the OR methods was estimated as the probability of reaching the ACMG/AMP PS4 criterion (OR > 5.0, CI not including 1.0, *p* value <0.05). Following the analyses results of the simulated datasets, optimal LR cut-offs (to maximize power and minimize type I error) are used to define ACMG/AMP evidence strengths for the 92 variants included in the BCAC OncoArray dataset.

## 3. Results

### 3.1. Simulated Datasets

Based on the simulation results for high-risk *BRCA1* (RR > 9) and *BRCA2* (RR > 5) variants, LR of strong and very strong evidence in favor of pathogenicity (LR ≥ 18.7) and of at least supporting evidence against pathogenicity (LR ≤ 0.48) should be used in order to maintain a high power (>80%) and low type I error (<0.05) (Supplementary Table S2).

**Figure 1:**
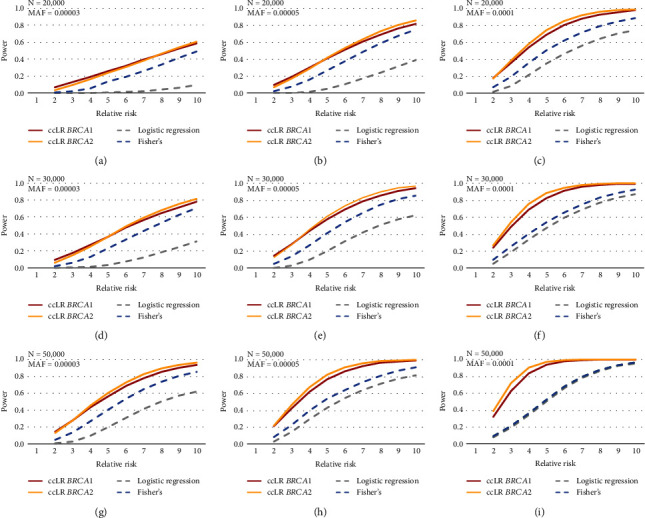
Performance of the case-control likelihood ratio method and odds ratio analysis in providing at least strong ACMG/AMP evidence in favor of pathogenicity (LR ≥ 18.7) using simulated datasets. Power equals the probability of reaching at least strong pathogenic ACMG/AMP evidence. Genotype data simulations were carried out for causal variants conferring disease relative risk between 2 and 10. We performed 10,000 simulations for each case scenario. Results represent simulated case-control data for 20,000 (a–c), 30,000 (d–f), or 50,000 (g–i) breast cancer cases and controls and minor allele frequency of 0.00003 (a, d, g), 0.00005 (b, e, h), or 0.0001 (c, f, i). ccLR: case-control likelihood ratio; MAF: minor allele frequency; *N*: sample size.

Results for all measures in all simulated datasets show that the power to achieve strong evidence in favour of pathogenicity is consistently greater for the case-control LR method using age-specific breast cancer risks compared to standard OR analysis methods (Figure 1, Supplementary Table S2). The power to correctly categorize variants with a RR comparable to a typical *BRCA1* PV was >80% in all scenarios except for small datasets (*N* ≤ 30,000) with causal variants present at a lower frequency (MAF = 0.00003) (Figure 1(a)).

**Figure 2:**
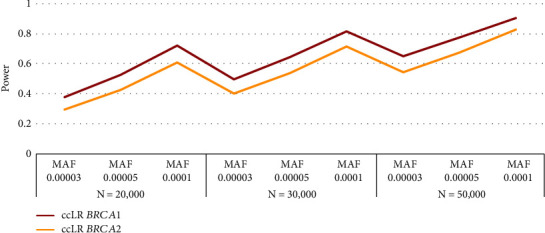
Performance of the case-control likelihood ratio method in providing ACMG/AMP evidence against pathogenicity, using simulated datasets. Power equals to the probability of reaching at least supporting benign ACMG/AMP evidence (LR ≤0.48) when the relative risk was set to 1. We performed 10,000 simulations for each case scenario. Results represent simulated case-control data for 20,000, 30,000, or 50,000 breast cancer cases and controls and minor allele frequency of 0.00003, 0.00005, or 0.0001. ccLR: case-control likelihood ratio; MAF: minor allele frequency; *N*: sample size.

In addition, the case-control LR method can also be used to obtain evidence against pathogenicity, something that cannot be achieved using standard OR analysis methods. Results from simulated case-control datasets of benign variants (RR of 1, Figure 2) show that the case-control LR method using the age-specific RRs of the “average” *BRCA1* PV exhibits adequate power (>80%) to identify variants with evidence against pathogenicity (LR ≤ 0.48) for larger datasets (*N* ≥ 30,000) and a MAF of 0.0001.

The implementation of the method to account for datasets with missing information, assuming the same age for all individuals, demonstrated reduced power and increased type I error in all simulations. However, the type I error was still less than 0.05 in all cases (Supplementary Figures S1 and S2, Supplementary Table S3).

### 3.2. BCAC OncoArray Dataset

#### 3.2.1. Logistic Regression Results

Using logistic regression, two *BRCA2* variants (2%) (Table 1) reached strong pathogenic evidence following the ACMG/AMP classification criterion (PS4 criterion, OR > 5, *p* value <0.05, and CI not including 1.0) [2]. Detailed logistic regression results for all variants are shown in Supplementary Table S4.

#### 3.2.2. Case-Control LRs and ACMG/AMP Code Strengths

In the country-stratified baseline analysis (using the breast cancer ORs estimated from BRIDGES [15]), evidence in favor of pathogenicity (defined as LR ≥ 18.70 following the simulation cut-offs) was achieved for 6 variants (6.5%) (Table 2), of which 3 variants were assigned very strong and another 3 strong strengths. Evidence against pathogenicity (defined as LR ≤ 0.48) was observed for 59 variants (64.1%), of which 26 were assigned very strong, 14 strong, 7 moderate, and 12 supporting strengths. The results for the remaining 27 variants (29.3%) were uninformative. Case-control LRs and corresponding ACMG/AMP code strengths for all 92 *BRCA1* and *BRCA2* variants are shown in Supplementary Table S4. The different sensitivity analyses did not show any major discrepancies in the estimated LRs (Supplementary Table S5).

## 4. Discussion

This study provides a detailed description of the methodology to calculate case-control LRs for rare variants using case-control data based on age- and gene-specific relative risks and age information for noncarriers. The LRs are calculated by comparing the likelihood of the distribution of the variant of interest in cases and controls under the hypothesis that the variant has similar age-specific relative risks as the “average” pathogenic variant, compared to the hypothesis that it is not associated with increased (or decreased) disease risk. We evaluated the method using simulated datasets and further applied it to derive LRs for pathogenicity for individual variants from the analysis of genotype data from a large case-control study. These can now be used in combination with other evidence to inform variant classification—either according to ACMG/AMP classification standards and guidelines [2, 3] or using multifactorial likelihood modelling approaches [4, 11]. Further, we provide user-friendly scripts and preformatted Excel calculators to facilitate the future implementation of this method for the calculation of case-control LRs. These resources may be readily applied for the calculation of LRs to be used in the classification of VUS in the *BRCA1* and *BRCA2* and other disease susceptibility genes with known penetrance values.

Notably, our results demonstrate the improved performance of our LR-based method for assessing variant pathogenicity as it considers gene- and age-specific penetrance for carriers and age information for noncarriers. Using simulated case-control datasets, we show that the case-control LR method using age-specific breast cancer ORs from high-penetrance genes (e.g., *BRCA1* and *BRCA2*) outperforms other OR analysis methods. These observations reflect the fact that the method presented here is more suitable for the analysis of rare variants in a case-control setting. We further provide cut-offs of LRs in favor or against pathogenicity to be used in a real setting.

Analysis of the BCAC OncoArray data using our proposed method provided informative pathogenic ACMG/AMP classification evidence for six out of the 92 variants analyzed. Furthermore, 59 variants reached evidence against pathogenicity, something that is not directly measured as a code strength through classical calculations of ORs. Given that, *a priori*, the vast majority of rare sequence variants (e.g., *BRCA1* and *BRCA2*) will be neutral with respect to risk, this is a key advantage of our approach. In contrast, using logistic regression analysis, the informative ACMG/AMP classification criterion PS4 (OR > 5.0, *p* value <0.05, and CI not including 1.0) was reached only for two variants.

There are possible caveats that should be recognized. The selection of cases or controls for a family history of cancer would affect the carrier probabilities. The likelihood ratios would then be inaccurate, but in principle, this could be considered by incorporating family history into the likelihoods, if known. Depletion of cases with known pathogenic variants by prior clinical sequencing could also bias the likelihood ratios; therefore, the method is best applied to population-based case-control studies. For these reasons, we highlight the ACMG/AMP recommendation to review all available evidence for/against pathogenicity for a given variant and to denote obviously conflicting findings for different evidence types, before assigning a final classification. A conservative approach may be to assign case-control weight with a cap, for example, at moderate strength for or against pathogenicity.

Our method gains power in part because it leverages data on individual-level age, but we have to acknowledge that age is not always available. The method can be implemented more approximately by assuming that individuals with unknown information are of the same age, but this reduces power because the expectation that carriers of risk variants develop the disease at a younger age is then not utilised. It may also increase type I error because the likelihood ratio may be calculated for an age that is not appropriate for the dataset (for example, if the dataset consists predominantly of older individuals), although the type I error was still low in the simulations we considered. In the tabulated, preformatted calculator, we allow the user to incorporate individuals of unknown age at diagnosis or interview into any of the age groups specified. A conservative approach would be to include individuals of unknown age in the oldest age group. In this way, case-control genotypes from both existing data and new series, with and without age data, can be incorporated. However, we would like to emphasize that pooling series, particularly from different populations with different age/ethnicity structures or with different genotyping technologies, can lead to biased results. Ideally, datasets should be analysed separately, and the overall likelihood ratio generated by multiplying the study-specific likelihood ratios.

## 5. Conclusions

This manuscript describes in detail a novel method used for the calculation of the case-control LR to provide evidence of variant pathogenicity. This LR method is more informative compared to logistic regression analysis (or an OR calculation based on contingency tables and Fisher's exact test). It improves power as it considers age- and gene-specific penetrance values and age information for noncarriers and can provide both evidence in favor of and against pathogenicity. In addition, this method can also be implemented towards the classification of VUS in any disease susceptibility gene for which disease penetrance has been reliably estimated. Open-access scripts and preformatted Excel calculators with code and instructions on how to use the method are available at the following address: https://github.com/BiostatUnitCING/ccLR.

## Figures and Tables

**Table 1:** Statistically significant associated variants with breast cancer risk estimated by logistic regression (based on the ACMG/AMP PS4 criterion).

Gene	Variant_ID (GRCh37/hg19)	HGVS nucleotide	HGVS protein	Variant carriers		OR (95% CI)	*p* value
				Cases	Controls		
				*N* (frequency)	*N* (frequency)		
*BRCA2*	chr13_32937506_C_G	c.8167G > C	p. (Asp2723His)	18/72392 (2.49 × 10^−4^)	1/50680 (1.97 × 10^−5^)	12.30 (1.66-91.23)	0.014
	chr13_32954180_C_T	c.9154C > T	p. (Arg3052Trp)	10/72563 (1.38 × 10^−4^)	1/50779 (1.97 × 10^−5^)	8.32 (1.04-66.48)	0.045

Variant nomenclature according to *BRCA2* (NM_000059.3, NP_000050.2). OR: odds ratio; CI: confidence interval.

**Table 2:** Variants with informative LRs in favor of pathogenicity, estimated by the baseline analysis.

Gene	Variant_ID (GRCh37/hg19)	HGVS nucleotide	HGVS protein	Variant carriers		LR
				Cases	Controls	
				N (frequency)	N (frequency)	
*BRCA1*	chr17_41234451_A_G	c.4327C > T	p.(Arg1443^∗^)	11/72558 (1.52 × 10^−4^)	3/50781 (5.91 × 10^−5^)	526.71
	chr17_41215947_T_G	c.5096G > T	p.(Arg1699Leu)	17/72560 (2.34 × 10^−4^)	3/50780 (5.91 × 10^−5^)	307.47

*BRCA2*	chr13_32937506_C_G	c.8167G > C	p.(Asp2723His)	18/72392 (2.49 × 10^−4^)	1/50680 (1.97 × 10^−5^)	8193.33
	chr13_32953453_A_G	c.8755-1G > A	p.?	3/72562 (4.13 × 10^−5^)	—	41.18
	chr13_32954180_C_T	c.9154C > T	p.(Arg3052Trp)	10/72563 (1.38 × 10^−4^)	1/50779 (1.97 × 10^−5^)	86.82
	chr13_32968940_A_T	c.9371A > T	p.(Asn3124Ile)	16/72548 (2.21 × 10^−4^)	—	3530.99

Variant nomenclature according to *BRCA1* (NM_007294.4, NP_009225.1), *BRCA2* (NM_000059.3, NP_000050.2). LR: likelihood ratio.

## Data Availability

All scripts allowing for replication of all analyses are available in the supplementary files and public repository (https://github.com/BiostatUnitCING/ccLR). Requests for the genotyped BCAC raw data can be made to the Data Access Coordination Committee (DACC) of BCAC (http://bcac.ccge.medschl.cam.ac.uk/).

## Supplementary Materials

Supplementary 1 Supplementary File 1: Case-control likelihood ratio (LR) method presented in detail.

Supplementary 2 Supplementary Table S1: BCAC studies participating in the case-control likelihood ratio analysis with the number of cases and controls. Supplementary Table S2: Power calculations of the case-control likelihood ratio method and odds ratio analysis methods using a simulated case-control datasets. Supplementary Table S2a: Power calculations using a relative risk of 1. Supplementary Table S2b: Power calculations using relative risk of 2 to 10. Supplementary Table S3: Power calculations of the case-control likelihood ratio method and odds ratio analysis methods using simulated case-control datasets of assumed same age. Supplementary Table S3a: Power calculations using relative risk of 1. Supplementary Table S3b: Power calculations using a relative risk of 2 to 10. Supplementary Table S4: Case-control evidence for the 92 *BRCA1* and *BRCA2* variants included in the BCAC OncoArray dataset.

Supplementary 3 Supplementary Table S5: Case-control likelihood ratios and evidence for assignment to ACMG/AMP code strengths for the 92 *BRCA1* and *BRCA2* variants using country-specific analyses with different penetrance models.

Supplementary 4 Supplementary Figure S1: Performance of the case-control likelihood ratio method and odds ratio analysis in providing at least strong ACMG/AMP evidence in favor of pathogenicity (LR ≥ 18.7), using simulated datasets of assumed same age. Power equals the probability of reaching at least strong pathogenic ACMG/AMP evidence. Genotype data simulations were carried out for causal variants conferring disease relative risk between 2 and 10. We performed 10,000 simulations for each case scenario. Results represent simulated case-control data for 20,000 (A–C), or 30,000 (D–F) or 50,000 (G–I) breast cancer cases and controls, and minor allele frequency of 0.00003 (A–G), 0.00005 (B–H), or 0.0001 (C–I). ccLR: case-control likelihood ratio; MAF: minor allele frequency; *N*: sample size. Supplementary Figure S2: performance of the case-control likelihood ratio method in providing ACMG/AMP evidence against pathogenicity using simulated datasets of assumed same age. Power equals the probability of reaching at least supporting benign ACMG/AMP evidence (*LR* ≤ 0.48) when the relative risk was set to 1. We performed 10,000 simulations for each case scenario. Results represent simulated case-control data for 20,000, 30,000, or 50,000 breast cancer cases and controls and minor allele frequency of 0.00003, 0.00005, or 0.0001. ccLR: case-control likelihood ratio; MAF: minor allele frequency; *N*: sample size.

## References

[1] Eccles D. M., Mitchell G., Monteiro A. N. A. (2015). *BRCA1* and *BRCA2* genetic testing–pitfalls and recommendations for managing variants of uncertain clinical significance. *Annals of Oncology*.

[2] Richards S., Aziz N., Bale S. (2015). Standards and guidelines for the interpretation of sequence variants: a joint consensus recommendation of the American College of Medical Genetics and Genomics and the Association for Molecular Pathology. *Genetics in Medicine*.

[3] Tavtigian S. V., Greenblatt M. S., Harrison S. M. (2018). Modeling the ACMG/AMP variant classification guidelines as a Bayesian classification framework. *Genetics in Medicine*.

[4] Goldgar D. E., Easton D. F., Deffenbaugh A. M., Monteiro A. N., Tavtigian S. V., Couch F. J. (2004). Integrated evaluation of DNA sequence variants of unknown clinical significance: application to *BRCA1* and *BRCA2*. *The American Journal of Human Genetics*.

[5] Goldgar D. E., Easton D. F., Byrnes G. B. (2008). Genetic evidence and integration of various data sources for classifying uncertain variants into a single model. *Human Mutation*.

[6] Spurdle A. B., Healey S., Devereau A. (2012). ENIGMA–evidence-based network for the interpretation of germline mutant alleles: an international initiative to evaluate risk and clinical significance associated with sequence variation in *BRCA1* and *BRCA2* genes. *Human Mutation*.

[7] Easton D. F., Deffenbaugh A. M., Pruss D. (2007). A systematic genetic assessment of 1,433 sequence variants of unknown clinical significance in the *BRCA1* and *BRCA2* breast cancer-predisposition genes. *The American Journal of Human Genetics*.

[8] Spurdle A. B., EMBRACE Group, Couch F. J. (2014). Refined histopathological predictors of *BRCA1* and *BRCA2* mutation status: a large-scale analysis of breast cancer characteristics from the BCAC, CIMBA, and ENIGMA consortia. *Breast Cancer Research*.

[9] Belman S., Parsons M. T., Spurdle A. B., Goldgar D. E., Feng B. J. (2020). Considerations in assessing germline variant pathogenicity using cosegregation analysis. *Genetics in Medicine*.

[10] Thompson D., Easton D. F., Goldgar D. E. (2003). A full-likelihood method for the evaluation of causality of sequence variants from family data. *American Journal of Human Genetics*.

[11] Parsons M. T., Tudini E., Li H. (2019). Large scale multifactorial likelihood quantitative analysis of BRCA1 and BRCA2 variants: an ENIGMA resource to support clinical variant classification. *Human Mutation*.

[12] Michailidou K., Lindström S., Dennis J. (2017). Association analysis identifies 65 new breast cancer risk loci. *Nature*.

[13] Hu C., Hart S. N., Gnanaolivu R. (2021). A population-based study of genes previously implicated in breast cancer. *New England Journal of Medicine*.

[14] Coleman J. R., Euesden J., Patel H., Folarin A. A., Newhouse S., Breen G. (2016). Quality control, imputation and analysis of genome-wide genotyping data from the Illumina HumanCoreExome microarray. *Briefings in Functional Genomics*.

[15] Dorling L., Carvalho S., Allen J. (2021). Breast cancer risk genes - association analysis in more than 113,000 women. *New England Journal of Medicine*.

[16] Antoniou A., Pharoah P. D. P., Narod S. (2003). Average risks of breast and ovarian cancer associated with *BRCA1* or *BRCA2* mutations detected in case series unselected for family history: a combined analysis of 22 studies. *American Journal of Human Genetics*.

[17] Kuchenbaecker K. B., Hopper J. L., Barnes D. R. (2017). Risks of breast, ovarian, and contralateral breast cancer for *BRCA1* and *BRCA2* mutation carriers. *JAMA*.

[18] Li H., Engel C., de la Hoya M. (2022). Risks of breast and ovarian cancer for women harboring pathogenic missense variants in *BRCA1* and *BRCA2* compared with those harboring protein truncating variants. *Genetics in Medicine*.

[19] Agresti A. (2003). *Categorical Data Analysis*.

