# Supplementary material for: A Likelihood Ratio Approach for Utilizing Case-Control Data in the Clinical Classification of Rare Sequence Variants: Application to BRCA1 and BRCA2
Source: Hum Mutat. 2023 Sep 14;2023:9961341. doi: 10.1155/2023/9961341 (PMC11080979; doi:10.1155/2023/9961341)
Supplement: Supplementary 1 — Supplementary File 1: Case-control likelihood ratio (LR) method presented in detail. [file 9961341.f1.docx]

**A likelihood ratio approach for utilizing case-control data in the clinical classification of rare sequence variants: application to *BRCA1* and *BRCA2* (Zanti et al. 2022)**

**Supplementary File 1.** Case-control Likelihood ratio (LR) method presented in detail

**Assumptions:**

Assume a dataset with N affected or unaffected individuals, in whom sequencing is performed.

Let $\boldsymbol{S}_{\boldsymbol{k}}\left( \boldsymbol{t} \right)\boldsymbol{=}\mathbf{exp}\boldsymbol{(-}\int_{\boldsymbol{0}}^{\boldsymbol{t}} \boldsymbol{\lambda}_{\boldsymbol{0}}\left( \boldsymbol{t} \right)\boldsymbol{e}^{\boldsymbol{\beta}\left( \boldsymbol{t} \right)\boldsymbol{k}}\boldsymbol{dt}\boldsymbol{)}$ be the probability that an individual with genotype *k* remains unaffected at age *t* (survival function).

For a variant carrier (*k* = 1), $S_{k}\left( t \right)$ is given by:

$$S_{1}(t)=exp(- \lambda_{0}\left( t \right)e^{\beta\left( t \right)}t)$$

For a non-carrier (*k* = 0), $S_{k}\left( t \right)$ is given by:

$$S_{0}(t)=exp(- \lambda_{0}\left( t \right)t)$$

Where both $S_{1}$and $S_{0}$ should be calculated for a given variant.

Let $\boldsymbol{f}_{\boldsymbol{k}}\left( \boldsymbol{t} \right)\boldsymbol{=}\boldsymbol{S}_{\boldsymbol{k}}\left( \boldsymbol{t} \right)\boldsymbol{\times}\boldsymbol{e}^{\boldsymbol{\beta}\left( \boldsymbol{t} \right)\boldsymbol{k}}$ be the corresponding probability that an individual with genotype *k* is affected at age t (hazard function).

$\boldsymbol{\lambda}_{\boldsymbol{0}}\left( \boldsymbol{t} \right)$, the baseline incidence, which is equals to population incidence and $\boldsymbol{\beta}\left( \boldsymbol{t} \right)$, the age-specific log-relative risks in carriers of an assumed pathogenic variant, are assumed known, where both $S_{k}\left( t \right)$ and $f_{k}\left( t \right)$ are fixed.

**Conditional case-control likelihood ratio (“ccLR”) calculation:**

The likelihood under the alternative hypothesis (H_1_) (i.e., variant is pathogenic) is given by:

$$\frac{\prod_{j}^{N} S_{v_{j}}(t_{j})e^{\beta(t_{j})d_{j}v_{j}}}{\sum_{\tau} \prod_{j=1}^{N} S_{v_{\tau(j)}}(t_{j})e^{\beta(t_{j})d_{j}v_{\tau(j)}}}$$

Where the sum is over all possible permutations $\tau$ of the variants ($\Sigma_{\tau}$). For individual j, t_j_ is the age at diagnosis or last observation, d_j_ = 0 or 1 is the disease status and v_j_ = 0 or 1 is the variant genotype status.

Under the null hypothesis of no association (H_0_) (i.e., variant is benign), the corresponding likelihood is:

$\frac{\prod_{j}^{N} S_{0}(t_{j})}{\sum_{\tau} \prod_{j=1}^{N} S_{0}(t_{j})}$=1/N!

Since genotypes (v_j_) take values of 0 and 1, the null hypothesis can be simplified to the $\left( \begin{matrix} N \\ K \end{matrix} \right)$ combinations:

$$\frac{\prod_{j}^{N} S_{v_{j}}(t_{j})e^{\beta(t_{j})d_{j}v_{j}}}{\sum_{\tau} \prod_{j=1}^{N} S_{v_{\tau(j)}}(t_{j})e^{\beta(t_{j})d_{j}v_{\tau(j)}}}$$

Where the sum in the denominator is over the $\left( \begin{matrix} N \\ K \end{matrix} \right)$ unique combinations of genotypes.

Assuming there is just one variant carrier (and *N*-1 non-carriers), the likelihood reduces to:

$$\frac{S_{1}(t_{j})e^{\beta(t_{j})d_{j}}\prod_{l\neq j}^{N} S_{0}(t_{l})}{\sum_{v_{j}=1}^{N} S_{1}(t_{v_{j}})e^{\beta(t_{v_{j}})d_{v_{j}}}\prod_{l\neq v_{j}} S_{0}(t_{l})}$$

$$=\frac{S_{1}\left( t_{j} \right)e^{\beta\left( t_{j} \right)d_{j}}/S_{0}(t_{j})}{\sum_{v_{j}=1}^{N} S_{1}\left( t_{v_{j}} \right)e^{\beta\left( t_{v_{j}} \right)d_{v_{j}}}/S_{0}(t_{v_{j}})}$$

If there are K number of carriers, and assuming a low rate of carriers, permutations (sampling without replacement) should be equivalent to sampling with replacement, where the resulting likelihood should be approximately equal to:

$$\frac{\prod_{v_{j}=1} S_{1}\left( t_{j} \right)e^{\beta\left( t_{j} \right)d_{j}}/S_{0}(t_{j})}{\left( \sum_{j}^{N} {S_{1}(t_{j})e}^{\beta(t_{j})d_{j}}/S_{0}(t_{j}) \right)^{K}}$$

Under the null hypothesis (H_0_) the above equals $1/N^{K}$. The likelihood ratio then reduces to:

$$ccLR= \frac{\prod_{v_{j}=1} S_{1}\left( t_{j} \right)e^{\beta\left( t_{j} \right)d_{j}}/S_{0}(t_{j})}{\left( \sum_{j}^{N} {S_{1}(t_{j})e}^{\beta(t_{j})d_{j}}/S_{0}(t_{j}) \right)^{K}} / \frac{1}{N^{K}}$$
