# Supplementary material for: A Likelihood Ratio Approach for Utilizing Case-Control Data in the Clinical Classification of Rare Sequence Variants: Application to BRCA1 and BRCA2
Source: Hum Mutat. 2023 Sep 14;2023:9961341. doi: 10.1155/2023/9961341 (PMC11080979; doi:10.1155/2023/9961341)
Supplement: Supplementary 2 — Supplementary Table S1: BCAC studies participating in the case-control likelihood ratio analysis with the number of cases and controls. Supplementary Table S2: Power calculations of the case-control likelihood ratio method and odds ratio analysis methods using a simulated case-control datasets. Supplementary Table S2a: Power calculations using a relative risk of 1. Supplementary Table S2b: Power calculations using relative risk of 2 to 10. Supplementary Table S3: Power calculations of the case-control likelihood ratio method and odds ratio analysis methods using simulated case-control datasets of assumed same age. Supplementary Table S3a: Power calculations using relative risk of 1. Supplementary Table S3b: Power calculations using a relative risk of 2 to 10. Supplementary Table S4: Case-control evidence for the 92 BRCA1 and BRCA2 variants included in the BCAC OncoArray dataset. [file 9961341.f2.docx]

| **Study name** | **Study Acronymn** | **Country** | **Study Design** | **Cases** | **Controls** |
| --- | --- | --- | --- | --- | --- |
| The Two Sister Study | 2SISTER | USA | Cases from sister-matched case-control study | 1064 | 0 |
| Australian Breast Cancer Family Study | ABCFS | Australia | Population-based case-control study | 1115 | 187 |
| Amsterdam Breast Cancer Study | ABCS | Netherlands | Non-population-based study | 347 | 189 |
| Australian Breast Cancer Tissue Bank | ABCTB | Australia | Non-population-based study | 949 | 374 |
| Agricultural Health Study | AHS | USA | Prospective cohort study | 476 | 1066 |
| Bavarian Breast Cancer Cases and Controls | BBCC | Germany | Non-population-based study | 410 | 252 |
| British Breast Cancer Study | BBCS | UK | Population-based case-control study | 121 | 441 |
| Breast Cancer Employment and Environment Study | BCEES | Australia | Population-based case-control study | 778 | 828 |
| New York Breast Cancer Family Registry | BCFR-NY | USA | Clinic-based recruitment of families; family-based cohort | 454 | 27 |
| Philadelphia Breast Cancer Family Registry | BCFR-PA | USA | Clinic-based recruitment of families; family-based cohort | 139 | 0 |
| Utah Breast Cancer Family Registry | BCFR-UTAH | USA | Clinic-based recruitment of nonBRCA1/2 familial breast cancer cases; unaffected BRCA1/2 carriers as controls | 99 | 0 |
| Breast Cancer in Northern Israel Study | BCINIS | Israel | Population-based case-control study | 1431 | 717 |
| Breast Oncology Galicia Network | BREOGAN | Spain | Non-population-based study | 1374 | 721 |
| Breast Cancer Study of the University of Heidelberg | BSUCH | Germany | Hospital-based cases; healthy blood donator controls | 277 | 168 |
| Canadian Breast Cancer Study | CBCS | Canada | Population-based case-control study | 675 | 817 |
| Crete Cancer Genetics Program | CCGP | Greece | Hospital-based case-control study | 670 | 330 |
| CECILE Breast Cancer Study | CECILE | France | Population-based case-control study | 305 | 159 |
| Copenhagen General Population Study | CGPS | Denmark | Non-population-based study | 1401 | 711 |
| Cancer Prevention Study-II Nutrition Cohort | CPSII | USA | Prospective cohort study | 3000 | 2977 |
| California Teachers Study | CTS | USA | Prospective cohort study | 1150 | 609 |
| DietCompLyf Breast Cancer Survival Study | DIETCOMPLYF | UK | Prospective cohort study | 708 | 0 |
| European Prospective Investigation Into Cancer and Nutrition | EPIC | France, Germany, Greece, Italy, Spain, The Netherlands, and UK | Prospective cohort study | 3828 | 3625 |
| ESTHER Breast Cancer Study | ESTHER | Germany | Population-based case-control study | 296 | 187 |
| Family History Risk Study | FHRISK | UK | Clinic-based cohort study with a nested case-control study | 146 | 296 |
| German Familial Breast Group | GC-HBOC | Germany | Population-based case-control study | 3608 | 1591 |
| Gene Environment Interaction and Breast Cancer in Germany | GENICA | Germany | Population-based case-control study | 460 | 284 |
| A randomized phase II trial investigating the addition of carboplatin to neoadjuvant therapy for triple-negative and HER2-positive early breast cancer | GEPARSIXTO | Germany | Multicentre, prospective, randomized, open-label phase II study | 385 | 0 |
| Genetic Epidemiology Study of Breast Cancer by Age 50 | GESBC | Germany | Population-based case-control study | 356 | 180 |
| Hannover Breast Cancer Study | HABCS | Germany | Hospital-based case-control study | 921 | 863 |
| Hospital Clinico San Carlos | HCSC | Spain | Population-based study of priori sporadic breast cancer cases | 425 | 0 |
| Helsinki Breast Cancer Study | HEBCS | Finland | Hospital-based case-control study, plus additional familial cases | 279 | 176 |
| Hannover- Minsk Breast Cancer Study | HMBCS | Belarus | Hospital-based cases; population-based controls | 211 | 241 |
| Hannover-Ufa Breast Cancer Study | HUBCS | Russia | Hospital-based cases; population-based controls | 209 | 117 |
| Karolinska Breast Cancer Study | KARBAC | Sweden | Population and hospital-based cases; geographically matched controls | 503 | 0 |
| Karolinska Mammography Project for Risk Prediction of Breast Cancer - Cohort Study | KARMA | Sweden | Prospective cohort study | 2672 | 5942 |
| Kuopio Breast Cancer Project | KBCP | Finland | Population-based prospective clinical cohort | 553 | 244 |
| Leuven Multidisciplinary Breast Centre | LMBC | Belgium | Non-population-based study | 804 | 1265 |
| Macedonian Breast Cancer Study | MABCS | Macedonia | Hospital-based case-control study | 89 | 91 |
| Mammary Carcinoma Risk Factor Investigation | MARIE | Germany | Population-based case-control study | 506 | 282 |
| Milan Breast Cancer Study Group | MBCSG | Italy | Clinic-based recruitment of familial/early onset breast cancer patients (cases); population-based controls | 787 | 365 |
| Mayo Clinic Breast Cancer Study | MCBCS | USA | Non-population-based study | 926 | 221 |
| Melbourne Collaborative Cohort Study | MCCS | Australia | Prospective cohort study | 1050 | 977 |
| Multiethnic Cohort | MEC | USA | Prospective cohort study | 669 | 717 |
| Melanoma Inquiry of Southern Sweden | MISS | Sweden | Prospective cohort study | 698 | 1532 |
| Mayo Mammography Health Study | MMHS | USA | Prospective cohort study | 381 | 1631 |
| Memorial Sloan-Kettering Cancer Centre Study | MSKCC | USA | Case-control study | 138 | 0 |
| Montreal Gene-Environment Breast Cancer Study | MTLGEBCS | Canada | Population-based case-control study | 341 | 170 |
| Norwegian Breast Cancer Study | NBCS | Norway | Hospital-based case-control study | 1238 | 0 |
| Nashville Breast Health Study | NBHS | USA | Population-based case-control study | 672 | 642 |
| Northern California Breast Cancer Family Registry | NC-BCFR | USA | Non-population-based study | 773 | 151 |
| North Carolina Breast Cancer Study | NCBCS | USA | Population-based case-control study | 2322 | 976 |
| Nurses' Health Study | NHS | USA | Prospective cohort study | 1457 | 1672 |
| Nurses' Health Study 2 | NHS2 | USA | Prospective cohort study | 1450 | 1777 |
| Ontario Familial Breast Cancer Registry | OFBCR | Canada | Non-population-based study | 1661 | 373 |
| Leiden University Medical Centre Breast Cancer Study | ORIGO | Netherlands | Hospital-based prospective cohort study | 1051 | 655 |
| NCI Polish Breast Cancer Study | PBCS | Poland | Population-based case-control study | 1904 | 2022 |
| Karolinska Mammography Project for Risk Prediction of Breast Cancer - Case-Control Study | PKARMA | Sweden | Non-population-based study | 831 | 48 |
| The Prostate, Lung, Colorectal and Ovarian Cancer Screening Trial | PLCO | USA | Prospective cohort study | 2243 | 2533 |
| Prospective Study of Outcomes in Sporadic Versus Hereditary Breast Cancer | POSH | UK | Prospective cohort | 1082 | 0 |
| Evaluation of Predictive Factors regarding the Effectivity of Aromatase Inhibitor Therapy | PREFACE | Germany | Multicentre, prospective, randomized, open-label phase IV study | 2988 | 0 |
| Predicting the Risk Of Cancer At Screening Study | PROCAS | UK | Population-based case-control study | 647 | 1653 |
| Rotterdam Breast Cancer Study | RBCS | Netherlands | Hospital-based case-control study, Rotterdam area | 470 | 238 |
| Study of Epidemiology and Risk factors in Cancer Heredity | SEARCH | UK | Non-population-based study | 4049 | 2665 |
| The Sister Study | SISTER | USA | Prospective cohort study | 2016 | 1556 |
| Städtisches Klinikum Karlsruhe Deutsches Krebsforschungszentrum Study | SKKDKFZS | Germany | Hospital-based case-control study | 1093 | 0 |
| Swedish Mammography Cohort | SMC | Sweden | Prospective cohort study | 1501 | 701 |
| Simultaneous Study of Gemcitabine-Docetaxel Combination adjuvant treatment | SUCCESSB | Germany | Multicentre, prospective, randomized, open-label phase III study | 436 | 0 |
| Simultaneous Study of Docetaxel Based Anthracycline Free Adjuvant Treatment Evaluation | SUCCESSC | Germany | Multicentre, prospective, randomized, open-label phase III study | 2785 | 0 |
| Szczecin Breast Cancer Study | SZBCS | Poland | Hospital-based case-control study | 386 | 174 |
| Triple Negative Breast Cancer Consortium Study | TNBCC | Various | Population-based case-control study | 619 | 0 |
| UCI Breast Cancer Study | UCIBCS | USA | Non-population-based study | 501 | 257 |
| UK Breakthrough Generations Study | UKBGS | UK | Prospective cohort study | 1629 | 704 |
| UK Ovarian Cancer Population Study | UKOPS | UK | Hospital-based ovarian case-control study | 0 | 951 |
| US Radiologic Technologists Study | USRT | USA | Non-population-based study | 1669 | 1669 |
| **Total** | | | | **75657** | **52987** |

| **Relative Risk** | **Sample Size** | **Minor Allele Frequency** | ***BRCA1*** | | | | ***BRCA2*** | | | | **Power**  **-**  **Logistic Regression Analysis (OR)§** | **Power**  **-**  **Fisher's exact test (OR)§** |
| --- | --- | --- | --- | --- | --- | --- | --- | --- | --- | --- | --- | --- |
|  |  |  | **†Power**  **(LR ≤ 0.053)** | **†Power (LR ≤ 0.48)** | **‡Type I error (LR ≥ 18.7)** | **‡Type I error (LR ≥ 2.08)** | **†Power**  **(LR ≤ 0.053)** | **†Power**  **(LR ≤ 0.48)** | **‡Type I error (LR ≥ 18.7)** | **‡Type I error (LR ≥ 2.08)** |  |  |
| 1 | 20000 | 0.00003 | 0.0208 | 0.3792 | 0.0012 | 0.1275 | 0.0104 | 0.2962 | 0.0006 | 0.1287 | - | - |
| 1 | 20000 | 0.00005 | 0.0739 | 0.5234 | 0.0024 | 0.1096 | 0.0400 | 0.4247 | 0.0012 | 0.1182 | - | - |
| 1 | 20000 | 0.0001 | 0.2764 | 0.7232 | 0.0067 | 0.0856 | 0.1678 | 0.6113 | 0.0046 | 0.1045 | - | - |
| 1 | 30000 | 0.00003 | 0.0578 | 0.4976 | 0.0017 | 0.1084 | 0.0301 | 0.3979 | 0.0011 | 0.1126 | - | - |
| 1 | 30000 | 0.00005 | 0.1729 | 0.6458 | 0.0042 | 0.0908 | 0.0980 | 0.5399 | 0.0028 | 0.1053 | - | - |
| 1 | 30000 | 0.0001 | 0.4696 | 0.8142 | 0.0054 | 0.0607 | 0.3122 | 0.7181 | 0.0059 | 0.0880 | - | - |
| 1 | 50000 | 0.00003 | 0.1739 | 0.6486 | 0.0039 | 0.0951 | 0.0955 | 0.5405 | 0.0022 | 0.1115 | - | - |
| 1 | 50000 | 0.00005 | 0.3791 | 0.7740 | 0.0058 | 0.0741 | 0.2383 | 0.6736 | 0.0051 | 0.0954 | - | - |
| 1 | 50000 | 0.0001 | 0.7209 | 0.9058 | 0.0053 | 0.0375 | 0.5415 | 0.8309 | 0.0080 | 0.0618 | - | - |

† Power equals to the probability of meeting benign ACMG/AMP evidence; LR ≤ 0.053 for at least strong benign evidence and LR ≤ 0.48 for at least supporting benign evidence.

‡ Type I error equals to the probability of meeting pathogenic ACMG/AMP evidence; LR ≥ 18.7 for at least strong pathogenic evidence and LR ≥ 2.08 for at least supporting pathogenic evidence.

§ Odds ratio analysis methods do not provide ACMG/AMP evidence against pathogenicity

LR, likelihood ratio. Genotype data simulations were carried out for variants conferring disease relative risk of 1, 2, 3, 4, 5, 6, 7, 8, 9 or 10, minor allele frequency of 0.0001, 0.00005 or 0.00003 and sample size of N = 20,000 (20,000 BC cases and 20,000 controls), 30,000 (30,000 BC cases and 30,000 controls) or 50,000 (50,000 BC cases and 50,000 controls). The number of permutations for each case scenario was 10,000 times.

| **Relative Risk** | **Sample Size** | **Minor Allele Frequency** | ***BRCA1*** | | | | ***BRCA2*** | | | | **Power - Logistic Regression Analysis (OR)§** | **Power - Fisher's exact test (OR)§** |
| --- | --- | --- | --- | --- | --- | --- | --- | --- | --- | --- | --- | --- |
|  |  |  | **†Power**  **(LR ≥ 18.7)** | **†Power**  **(LR ≥ 2.08)** | **‡Type I error**  **(LR ≤ 0.053)** | **‡Type I error**  **(LR ≤ 0.48)** | **†Power**  **(LR ≥ 18.7)** | **†Power**  **(LR ≥ 2.08)** | **‡Type I error**  **(LR ≤ 0.053)** | **‡Type I error**  **(LR ≤ 0.48)** |  |  |
| 2 | 20000 | 0.00003 | 0.0703 | 0.4341 | 0.0131 | 0.2087 | 0.0339 | 0.4697 | 0.0042 | 0.1279 | 0.0000 | 0.0046 |
| 2 | 20000 | 0.00005 | 0.1001 | 0.4142 | 0.0386 | 0.2913 | 0.0723 | 0.4477 | 0.0160 | 0.201 | 0.0004 | 0.0206 |
| 2 | 20000 | 0.0001 | 0.1778 | 0.4394 | 0.1148 | 0.3544 | 0.1726 | 0.4994 | 0.0526 | 0.2596 | 0.0149 | 0.0675 |
| 2 | 30000 | 0.00003 | 0.0912 | 0.4163 | 0.0321 | 0.2753 | 0.0601 | 0.4489 | 0.0131 | 0.1846 | 0.0004 | 0.0158 |
| 2 | 30000 | 0.00005 | 0.1451 | 0.4235 | 0.0820 | 0.335 | 0.1266 | 0.4671 | 0.0350 | 0.2417 | 0.0046 | 0.0474 |
| 2 | 30000 | 0.0001 | 0.2432 | 0.4655 | 0.1636 | 0.368 | 0.2690 | 0.5493 | 0.0776 | 0.2635 | 0.0523 | 0.0960 |
| 2 | 50000 | 0.00003 | 0.1452 | 0.4237 | 0.0839 | 0.3345 | 0.1280 | 0.4656 | 0.0348 | 0.2412 | 0.0045 | 0.0474 |
| 2 | 50000 | 0.00005 | 0.2123 | 0.4599 | 0.1440 | 0.3636 | 0.2174 | 0.5259 | 0.0681 | 0.2631 | 0.0327 | 0.0830 |
| 2 | 50000 | 0.0001 | 0.3233 | 0.5081 | 0.2016 | 0.3667 | 0.3890 | 0.6181 | 0.0987 | 0.2461 | 0.0782 | 0.0904 |
| 3 | 20000 | 0.00003 | 0.1317 | 0.5221 | 0.0101 | 0.1689 | 0.0935 | 0.5633 | 0.0027 | 0.1032 | 0.0004 | 0.0202 |
| 3 | 20000 | 0.00005 | 0.1933 | 0.5391 | 0.0302 | 0.214 | 0.1715 | 0.5890 | 0.0099 | 0.1371 | 0.0047 | 0.0740 |
| 3 | 20000 | 0.0001 | 0.3584 | 0.6273 | 0.0601 | 0.2131 | 0.3806 | 0.6990 | 0.0241 | 0.134 | 0.0823 | 0.1900 |
| 3 | 30000 | 0.00003 | 0.1739 | 0.5328 | 0.0257 | 0.2101 | 0.1467 | 0.5813 | 0.0070 | 0.134 | 0.0027 | 0.0563 |
| 3 | 30000 | 0.00005 | 0.2816 | 0.5890 | 0.0507 | 0.2193 | 0.2783 | 0.6497 | 0.0174 | 0.1423 | 0.0299 | 0.1403 |
| 3 | 30000 | 0.0001 | 0.4836 | 0.6882 | 0.0706 | 0.1889 | 0.5377 | 0.7780 | 0.0244 | 0.1073 | 0.1878 | 0.2506 |
| 3 | 50000 | 0.00003 | 0.2819 | 0.5876 | 0.0514 | 0.2217 | 0.2800 | 0.6512 | 0.0191 | 0.143 | 0.0305 | 0.1403 |
| 3 | 50000 | 0.00005 | 0.4266 | 0.6591 | 0.0683 | 0.2006 | 0.4638 | 0.7446 | 0.0261 | 0.1211 | 0.1461 | 0.2293 |
| 3 | 50000 | 0.0001 | 0.6268 | 0.7760 | 0.0661 | 0.1456 | 0.7213 | 0.8692 | 0.0206 | 0.0689 | 0.1929 | 0.2108 |
| 4 | 20000 | 0.00003 | 0.1899 | 0.5919 | 0.0085 | 0.1439 | 0.1604 | 0.6380 | 0.0021 | 0.0852 | 0.0017 | 0.0532 |
| 4 | 20000 | 0.00005 | 0.2999 | 0.6466 | 0.0213 | 0.1549 | 0.2851 | 0.7047 | 0.0061 | 0.0934 | 0.0179 | 0.1574 |
| 4 | 20000 | 0.0001 | 0.5363 | 0.7656 | 0.0304 | 0.1236 | 0.5804 | 0.8329 | 0.0092 | 0.0645 | 0.2128 | 0.3525 |
| 4 | 30000 | 0.00003 | 0.2702 | 0.6313 | 0.0186 | 0.156 | 0.2501 | 0.6873 | 0.0058 | 0.0932 | 0.0111 | 0.1304 |
| 4 | 30000 | 0.00005 | 0.4363 | 0.7111 | 0.0311 | 0.1403 | 0.4558 | 0.7788 | 0.0088 | 0.0789 | 0.0987 | 0.2696 |
| 4 | 30000 | 0.0001 | 0.6856 | 0.8439 | 0.0291 | 0.0852 | 0.7564 | 0.9060 | 0.0073 | 0.0401 | 0.3349 | 0.4025 |
| 4 | 50000 | 0.00003 | 0.4359 | 0.7113 | 0.0308 | 0.139 | 0.4565 | 0.7806 | 0.0091 | 0.0811 | 0.0997 | 0.2696 |
| 4 | 50000 | 0.00005 | 0.6199 | 0.8118 | 0.0289 | 0.101 | 0.6772 | 0.8801 | 0.0082 | 0.0491 | 0.2936 | 0.3942 |
| 4 | 50000 | 0.0001 | 0.8351 | 0.9171 | 0.0178 | 0.0453 | 0.9061 | 0.9675 | 0.0039 | 0.0149 | 0.3442 | 0.3635 |
| 5 | 20000 | 0.00003 | 0.2589 | 0.6513 | 0.0069 | 0.1186 | 0.2354 | 0.7002 | 0.0019 | 0.0676 | 0.0041 | 0.1370 |
| 5 | 20000 | 0.00005 | 0.4093 | 0.7339 | 0.0144 | 0.1122 | 0.4180 | 0.7877 | 0.0036 | 0.0632 | 0.0512 | 0.2675 |
| 5 | 20000 | 0.0001 | 0.6857 | 0.8641 | 0.0148 | 0.0646 | 0.7424 | 0.9165 | 0.0034 | 0.0276 | 0.3494 | 0.5043 |
| 5 | 30000 | 0.00003 | 0.3677 | 0.7161 | 0.0136 | 0.112 | 0.3662 | 0.7687 | 0.0038 | 0.0639 | 0.0330 | 0.2317 |
| 5 | 30000 | 0.00005 | 0.5707 | 0.8036 | 0.0178 | 0.0852 | 0.6078 | 0.8676 | 0.0041 | 0.0439 | 0.1998 | 0.4088 |
| 5 | 30000 | 0.0001 | 0.8234 | 0.9262 | 0.0099 | 0.0366 | 0.8861 | 0.9662 | 0.0021 | 0.0128 | 0.4736 | 0.5411 |
| 5 | 50000 | 0.00003 | 0.5681 | 0.8060 | 0.0184 | 0.0857 | 0.6092 | 0.8635 | 0.0049 | 0.0446 | 0.2008 | 0.4088 |
| 5 | 50000 | 0.00005 | 0.7699 | 0.8999 | 0.0137 | 0.0486 | 0.8270 | 0.9492 | 0.0033 | 0.0193 | 0.4306 | 0.5365 |
| 5 | 50000 | 0.0001 | 0.9405 | 0.9737 | 0.0044 | 0.0133 | 0.9745 | 0.9927 | 0.0002 | 0.0025 | 0.5062 | 0.5280 |
| 6 | 20000 | 0.00003 | 0.3228 | 0.7073 | 0.0058 | 0.0968 | 0.3031 | 0.7568 | 0.0015 | 0.0533 | 0.0105 | 0.1874 |
| 6 | 20000 | 0.00005 | 0.5136 | 0.8010 | 0.0093 | 0.0752 | 0.5354 | 0.8566 | 0.0021 | 0.0395 | 0.1030 | 0.3754 |
| 6 | 20000 | 0.0001 | 0.8029 | 0.9242 | 0.0082 | 0.0354 | 0.8547 | 0.9594 | 0.0017 | 0.0121 | 0.4677 | 0.6233 |
| 6 | 30000 | 0.00003 | 0.4711 | 0.7825 | 0.0089 | 0.0821 | 0.4855 | 0.8358 | 0.0019 | 0.044 | 0.0703 | 0.3340 |
| 6 | 30000 | 0.00005 | 0.6882 | 0.8762 | 0.0097 | 0.0505 | 0.7327 | 0.9255 | 0.0015 | 0.0231 | 0.3119 | 0.5411 |
| 6 | 30000 | 0.0001 | 0.9130 | 0.9636 | 0.0040 | 0.0165 | 0.9500 | 0.9859 | 0.0004 | 0.0037 | 0.5915 | 0.6580 |
| 6 | 50000 | 0.00003 | 0.6879 | 0.8757 | 0.0095 | 0.0517 | 0.7348 | 0.9230 | 0.0021 | 0.0229 | 0.3128 | 0.5411 |
| 6 | 50000 | 0.00005 | 0.8670 | 0.9518 | 0.0041 | 0.0225 | 0.9156 | 0.9795 | 0.0013 | 0.0065 | 0.5433 | 0.6426 |
| 6 | 50000 | 0.0001 | 0.9817 | 0.9927 | 0.0013 | 0.0038 | 0.9940 | 0.9983 | 0.0000 | 0.0008 | 0.6643 | 0.6824 |
| 7 | 20000 | 0.00003 | 0.3916 | 0.7533 | 0.0048 | 0.0773 | 0.3794 | 0.8042 | 0.0009 | 0.0405 | 0.0217 | 0.2570 |
| 7 | 20000 | 0.00005 | 0.6093 | 0.8534 | 0.0067 | 0.0528 | 0.6377 | 0.9015 | 0.0010 | 0.0249 | 0.1698 | 0.4855 |
| 7 | 20000 | 0.0001 | 0.8759 | 0.9587 | 0.0027 | 0.0168 | 0.9204 | 0.9829 | 0.0009 | 0.0047 | 0.5629 | 0.7163 |
| 7 | 30000 | 0.00003 | 0.5628 | 0.8348 | 0.0063 | 0.0565 | 0.5880 | 0.8837 | 0.0014 | 0.0290 | 0.1192 | 0.4356 |
| 7 | 30000 | 0.00005 | 0.7824 | 0.9235 | 0.0046 | 0.0284 | 0.8285 | 0.9577 | 0.0009 | 0.0099 | 0.4166 | 0.6497 |
| 7 | 30000 | 0.0001 | 0.9608 | 0.9850 | 0.0016 | 0.0060 | 0.9812 | 0.9958 | 0.0003 | 0.0016 | 0.6857 | 0.7489 |
| 7 | 50000 | 0.00003 | 0.7823 | 0.9244 | 0.0052 | 0.0291 | 0.8292 | 0.9603 | 0.0008 | 0.0115 | 0.4141 | 0.6497 |
| 7 | 50000 | 0.00005 | 0.9297 | 0.9766 | 0.0018 | 0.0108 | 0.9613 | 0.9920 | 0.0004 | 0.0017 | 0.6391 | 0.7379 |
| 7 | 50000 | 0.0001 | 0.9948 | 0.9985 | 0.0003 | 0.0006 | 0.9991 | 0.9957 | 0.0000 | 0.0002 | 0.7837 | 0.7996 |
| 8 | 20000 | 0.00003 | 0.4586 | 0.7986 | 0.0034 | 0.0643 | 0.4610 | 0.8435 | 0.0005 | 0.0313 | 0.0389 | 0.3361 |
| 8 | 20000 | 0.00005 | 0.6905 | 0.8950 | 0.0040 | 0.0350 | 0.7280 | 0.9346 | 0.0006 | 0.0141 | 0.2388 | 0.5841 |
| 8 | 20000 | 0.0001 | 0.9259 | 0.9767 | 0.0022 | 0.0094 | 0.9575 | 0.9907 | 0.0003 | 0.0024 | 0.6390 | 0.7929 |
| 8 | 30000 | 0.00003 | 0.6427 | 0.8784 | 0.0041 | 0.0408 | 0.6756 | 0.9208 | 0.0006 | 0.0196 | 0.1789 | 0.5304 |
| 8 | 30000 | 0.00005 | 0.8545 | 0.9526 | 0.0036 | 0.0169 | 0.8947 | 0.9776 | 0.0005 | 0.0054 | 0.5053 | 0.7419 |
| 8 | 30000 | 0.0001 | 0.9816 | 0.9940 | 0.0006 | 0.0025 | 0.9932 | 0.9985 | 0.0001 | 0.0004 | 0.7718 | 0.8310 |
| 8 | 50000 | 0.00003 | 0.8539 | 0.9514 | 0.0033 | 0.0174 | 0.8960 | 0.9774 | 0.0005 | 0.0049 | 0.5055 | 0.7419 |
| 8 | 50000 | 0.00005 | 0.9650 | 0.9891 | 0.0007 | 0.0042 | 0.9855 | 0.9968 | 0.0001 | 0.0008 | 0.7173 | 0.8126 |
| 8 | 50000 | 0.0001 | 0.9989 | 0.9998 | 0.0000 | 0.0000 | 0.9999 | 1.0000 | 0.0000 | 0.0000 | 0.8686 | 0.8807 |
| 9 | 20000 | 0.00003 | 0.5264 | 0.8362 | 0.0023 | 0.0493 | 0.5379 | 0.8784 | 0.0006 | 0.0220 | 0.0616 | 0.4129 |
| 9 | 20000 | 0.00005 | 0.7601 | 0.9244 | 0.0023 | 0.0238 | 0.8011 | 0.9580 | 0.0004 | 0.0087 | 0.3132 | 0.6738 |
| 9 | 20000 | 0.0001 | 0.9564 | 0.9890 | 0.0006 | 0.0038 | 0.9783 | 0.9966 | 0.0001 | 0.0007 | 0.7009 | 0.8472 |
| 9 | 30000 | 0.00003 | 0.7148 | 0.9111 | 0.0027 | 0.0282 | 0.7497 | 0.9453 | 0.0002 | 0.0117 | 0.2457 | 0.6242 |
| 9 | 30000 | 0.00005 | 0.9065 | 0.9701 | 0.0016 | 0.0105 | 0.9412 | 0.9884 | 0.0000 | 0.0027 | 0.5713 | 0.8100 |
| 9 | 30000 | 0.0001 | 0.9914 | 0.9975 | 0.0002 | 0.0009 | 0.9978 | 0.9995 | 0.0000 | 0.0002 | 0.8303 | 0.8863 |
| 9 | 50000 | 0.00003 | 0.9061 | 0.9703 | 0.0015 | 0.0102 | 0.9411 | 0.9881 | 0.0001 | 0.0027 | 0.5717 | 0.8100 |
| 9 | 50000 | 0.00005 | 0.9813 | 0.9951 | 0.0004 | 0.0018 | 0.9935 | 0.9991 | 0.0001 | 0.0003 | 0.7749 | 0.8697 |
| 9 | 50000 | 0.0001 | 0.9997 | 1.0000 | 0.0000 | 0.0000 | 1.0000 | 1.0000 | 0.0000 | 0.0000 | 0.9239 | 0.9331 |
| 10 | 20000 | 0.00003 | 0.5859 | 0.8686 | 0.0014 | 0.0360 | 0.6025 | 0.9088 | 0.0004 | 0.0162 | 0.0925 | 0.4917 |
| 10 | 20000 | 0.00005 | 0.8197 | 0.9505 | 0.0021 | 0.0144 | 0.8564 | 0.9747 | 0.0004 | 0.0049 | 0.3874 | 0.7473 |
| 10 | 20000 | 0.0001 | 0.9774 | 0.9938 | 0.0004 | 0.0022 | 0.9904 | 0.9984 | 0.0000 | 0.0005 | 0.7446 | 0.8889 |
| 10 | 30000 | 0.00003 | 0.7770 | 0.9352 | 0.0019 | 0.0193 | 0.8163 | 0.9637 | 0.0002 | 0.0074 | 0.3075 | 0.7027 |
| 10 | 30000 | 0.00005 | 0.9387 | 0.9835 | 0.0007 | 0.0052 | 0.9645 | 0.9939 | 0.0001 | 0.0011 | 0.6198 | 0.8540 |
| 10 | 30000 | 0.0001 | 0.9962 | 0.9990 | 0.0000 | 0.0004 | 0.9989 | 1.0000 | 0.0000 | 0.0000 | 0.8698 | 0.9235 |
| 10 | 50000 | 0.00003 | 0.9389 | 0.9829 | 0.0007 | 0.0049 | 0.9651 | 0.9937 | 0.0001 | 0.0012 | 0.6207 | 0.8540 |
| 10 | 50000 | 0.00005 | 0.9920 | 0.9985 | 0.0001 | 0.0003 | 0.9981 | 0.9998 | 0.0000 | 0.0001 | 0.8188 | 0.9095 |
| 10 | 50000 | 0.0001 | 1.0000 | 1.0000 | 0.0000 | 0 | 1.0000 | 1.0000 | 0.0000 | 0 | 0.9556 | 0.9638 |

† Power equals to the probability of meeting pathogenic ACMG/AMP evidence; LR ≥ 18.7 for at least strong pathogenic evidence and LR ≥ 2.08 for at least supporting pathogenic evidence.

‡ Type I error equals to the probability of meeting benign ACMG/AMP evidence; LR ≤ 0.053 for at least strong benign evidence and LR ≤ 0.48 for at least supporting benign evidence.

§ Power equals to the probability of meeting strong ACMG/AMP evidence (PS4 criterion, OR > 5.0, CI not including 1.0/p-value < 0.05)

LR, likelihood ratio. Genotype data simulations were carried out for variants conferring disease relative risk of 1, 2, 3, 4, 5, 6, 7, 8, 9 or 10, minor allele frequency of 0.0001, 0.00005 or 0.00003 and sample size of N = 20,000 (20,000 BC cases and 20,000 controls), 30,000 (30,000 BC cases and 30,000 controls) or 50,000 (50,000 BC cases and 50,000 controls). The number of permutations for each case scenario was 10,000 time

| **Relative Risk** | **Sample Size** | **Minor Allele Frequency** | ***BRCA1*** | | | | ***BRCA2*** | | | | **Power**  **-**  **Logistic Regression Analysis (OR)§** | **Power**  **-**  **Fisher's exact test (OR)§** |
| --- | --- | --- | --- | --- | --- | --- | --- | --- | --- | --- | --- | --- |
|  |  |  | **†Power**  **(LR ≤ 0.053)** | **†Power (LR ≤ 0.48)** | **‡Type I error (LR ≥ 18.7)** | **‡Type I error (LR ≥ 2.08)** | **†Power**  **(LR ≤ 0.053)** | **†Power**  **(LR ≤ 0.48)** | **‡Type I error (LR ≥ 18.7)** | **‡Type I error (LR ≥ 2.08)** |  |  |
| 1 | 20000 | 0.00003 | 0.0021 | 0.1207 | 0 | 0.066 | 2.00E-04 | 0.1107 | 0 | 0.066 | - | - |
| 1 | 20000 | 0.00005 | 0.0126 | 0.2545 | 0 | 0.1054 | 0.0019 | 0.2172 | 0 | 0.1054 | - | - |
| 1 | 20000 | 0.0001 | 0.0855 | 0.4915 | 7.00E-04 | 0.1258 | 0.028 | 0.407 | 4.00E-04 | 0.1266 | - | - |
| 1 | 30000 | 0.00003 | 0.0088 | 0.224 | 0 | 0.0978 | 0.0016 | 0.1928 | 0 | 0.0978 | - | - |
| 1 | 30000 | 0.00005 | 0.0429 | 0.3946 | 5.00E-04 | 0.1236 | 0.011 | 0.3261 | 2.00E-04 | 0.1237 | - | - |
| 1 | 30000 | 0.0001 | 0.1788 | 0.6197 | 0.0023 | 0.1149 | 0.0843 | 0.5234 | 0.0012 | 0.119 | - | - |
| 1 | 50000 | 0.00003 | 0.0429 | 0.3946 | 5.00E-04 | 0.1236 | 0.011 | 0.3261 | 2.00E-04 | 0.1237 | - | - |
| 1 | 50000 | 0.00005 | 0.1329 | 0.5698 | 0.0014 | 0.1228 | 0.0548 | 0.4696 | 7.00E-04 | 0.1248 | - | - |
| 1 | 50000 | 0.0001 | 0.3657 | 0.7481 | 0.0048 | 0.0914 | 0.2043 | 0.6516 | 0.0044 | 0.1167 | - | - |

† Power equals to the probability of meeting benign ACMG/AMP evidence; LR ≤ 0.053 for at least strong benign evidence and LR ≤ 0.48 for at least supporting benign evidence.

‡ Type I error equals to the probability of meeting pathogenic ACMG/AMP evidence; LR ≥ 18.7 for at least strong pathogenic evidence and LR ≥ 2.08 for at least supporting pathogenic evidence.

§ Odds ratio analysis methods do not provide ACMG/AMP evidence against pathogenicity

LR, likelihood ratio. Genotype data simulations were carried out for variants conferring disease relative risk of 1, 2, 3, 4, 5, 6, 7, 8, 9 or 10, minor allele frequency of 0.0001, 0.00005 or 0.00003 and sample size of N = 20,000 (20,000 BC cases and 20,000 controls), 30,000 (30,000 BC cases and 30,000 controls) or 50,000 (50,000 BC cases and 50,000 controls). The number of permutations for each case scenario was 10,000 times.

| **Relative Risk** | **Sample Size** | **Minor Allele Frequency** | ***BRCA1*** | | | | ***BRCA2*** | | | | **Power - Logistic Regression Analysis (OR)§** | **Power - Fisher's exact test (OR)§** |
| --- | --- | --- | --- | --- | --- | --- | --- | --- | --- | --- | --- | --- |
|  |  |  | **†Power**  **(LR ≥ 18.7)** | **†Power**  **(LR ≥ 2.08)** | **‡Type I error**  **(LR ≤ 0.053)** | **‡Type I error**  **(LR ≤ 0.48)** | **†Power**  **(LR ≥ 18.7)** | **†Power**  **(LR ≥ 2.08)** | **‡Type I error**  **(LR ≤ 0.053)** | **‡Type I error**  **(LR ≤ 0.48)** |  |  |
| 2 | 20000 | 0.00003 | 3.00E-04 | 0.3595 | 0.0011 | 0.1107 | 0 | 0.3595 | 2.00E-04 | 0.0573 | 0 | 0.0046 |
| 2 | 20000 | 0.00005 | 0.002 | 0.329 | 0.0061 | 0.2048 | 8.00E-04 | 0.3293 | 7.00E-04 | 0.1215 | 0.000422 | 0.0206 |
| 2 | 20000 | 0.0001 | 0.0215 | 0.3507 | 0.0344 | 0.3107 | 0.0141 | 0.3627 | 0.0106 | 0.2204 | 0.013837 | 0.0675 |
| 2 | 30000 | 0.00003 | 0.0012 | 0.332 | 0.0047 | 0.1843 | 5.00E-04 | 0.3321 | 6.00E-04 | 0.1085 | 0.000429 | 0.0158 |
| 2 | 30000 | 0.00005 | 0.0099 | 0.3324 | 0.0188 | 0.2709 | 0.0044 | 0.3355 | 0.005 | 0.1809 | 0.004151 | 0.0474 |
| 2 | 30000 | 0.0001 | 0.0555 | 0.3774 | 0.0702 | 0.3494 | 0.048 | 0.4208 | 0.0238 | 0.2493 | 0.055111 | 0.0960 |
| 2 | 50000 | 0.00003 | 0.0099 | 0.3324 | 0.0188 | 0.2709 | 0.0044 | 0.3355 | 0.005 | 0.1809 | 0.004455 | 0.0474 |
| 2 | 50000 | 0.00005 | 0.0385 | 0.3616 | 0.0538 | 0.3285 | 0.0297 | 0.3887 | 0.0177 | 0.2371 | 0.033817 | 0.0830 |
| 2 | 50000 | 0.0001 | 0.1179 | 0.3995 | 0.1209 | 0.3732 | 0.1172 | 0.4793 | 0.045 | 0.2552 | 0.0843 | 0.0904 |
| 3 | 20000 | 0.00003 | 0.0017 | 0.4229 | 6.00E-04 | 0.0793 | 6.00E-04 | 0.4229 | 1.00E-04 | 0.0535 | 0.00044 | 0.0202 |
| 3 | 20000 | 0.00005 | 0.0147 | 0.4619 | 0.0041 | 0.1378 | 0.0066 | 0.4634 | 8.00E-04 | 0.0861 | 0.004391 | 0.0740 |
| 3 | 20000 | 0.0001 | 0.106 | 0.565 | 0.0159 | 0.1702 | 0.0868 | 0.5957 | 0.0037 | 0.1118 | 0.083042 | 0.1900 |
| 3 | 30000 | 0.00003 | 0.0094 | 0.4518 | 0.003 | 0.1292 | 0.0036 | 0.4527 | 6.00E-04 | 0.0785 | 0.002475 | 0.0563 |
| 3 | 30000 | 0.00005 | 0.0533 | 0.5163 | 0.0099 | 0.1651 | 0.0341 | 0.5287 | 0.0021 | 0.1056 | 0.030689 | 0.1403 |
| 3 | 30000 | 0.0001 | 0.2256 | 0.6354 | 0.0235 | 0.1624 | 0.2204 | 0.6982 | 0.0054 | 0.0981 | 0.2009 | 0.2506 |
| 3 | 50000 | 0.00003 | 0.0533 | 0.5163 | 0.0099 | 0.1651 | 0.0341 | 0.5287 | 0.0021 | 0.1056 | 0.031291 | 0.1403 |
| 3 | 50000 | 0.00005 | 0.1663 | 0.6062 | 0.0202 | 0.1682 | 0.1536 | 0.6572 | 0.0046 | 0.1026 | 0.154415 | 0.2293 |
| 3 | 50000 | 0.0001 | 0.4382 | 0.7177 | 0.0278 | 0.128 | 0.4407 | 0.7956 | 0.0076 | 0.0691 | 0.2053 | 0.2108 |
| 4 | 20000 | 0.00003 | 0.0072 | 0.5418 | 7.00E-04 | 0.0712 | 0.0024 | 0.5419 | 1.00E-04 | 0.0405 | 0.001688 | 0.0532 |
| 4 | 20000 | 0.00005 | 0.0486 | 0.6013 | 0.0022 | 0.0962 | 0.0276 | 0.6057 | 4.00E-04 | 0.055 | 0.015921 | 0.1574 |
| 4 | 20000 | 0.0001 | 0.27 | 0.7395 | 0.0062 | 0.0882 | 0.2513 | 0.7814 | 0.0017 | 0.0467 | 0.2201 | 0.3525 |
| 4 | 30000 | 0.00003 | 0.0357 | 0.5805 | 0.0024 | 0.0931 | 0.0181 | 0.5828 | 4.00E-04 | 0.0512 | 0.010941 | 0.1304 |
| 4 | 30000 | 0.00005 | 0.1492 | 0.6811 | 0.0045 | 0.0974 | 0.1191 | 0.701 | 0.0012 | 0.0569 | 0.102782 | 0.2696 |
| 4 | 30000 | 0.0001 | 0.4715 | 0.8169 | 0.0079 | 0.0652 | 0.4692 | 0.866 | 0.0015 | 0.0308 | 0.3532 | 0.4025 |
| 4 | 50000 | 0.00003 | 0.1492 | 0.6811 | 0.0045 | 0.0974 | 0.1191 | 0.701 | 0.0012 | 0.0569 | 0.101481 | 0.2696 |
| 4 | 50000 | 0.00005 | 0.3726 | 0.7872 | 0.0066 | 0.0716 | 0.3642 | 0.8328 | 0.0024 | 0.0368 | 0.3116 | 0.3942 |
| 4 | 50000 | 0.0001 | 0.7523 | 0.9103 | 0.0063 | 0.0277 | 0.7609 | 0.9467 | 0.0012 | 0.012 | 0.3579 | 0.3635 |
| 5 | 20000 | 0.00003 | 0.02 | 0.6111 | 5.00E-04 | 0.0584 | 0.0089 | 0.6115 | 0 | 0.03 | 0.003918 | 0.1370 |
| 5 | 20000 | 0.00005 | 0.1168 | 0.7085 | 0.0015 | 0.0652 | 0.0759 | 0.7135 | 1.00E-04 | 0.0357 | 0.048245 | 0.2675 |
| 5 | 20000 | 0.0001 | 0.4587 | 0.8548 | 0.003 | 0.0407 | 0.446 | 0.8892 | 6.00E-04 | 0.0187 | 0.3659 | 0.5043 |
| 5 | 30000 | 0.00003 | 0.0836 | 0.6865 | 0.0013 | 0.0638 | 0.0495 | 0.6908 | 1.00E-04 | 0.0339 | 0.03268 | 0.2317 |
| 5 | 30000 | 0.00005 | 0.296 | 0.7969 | 0.0025 | 0.0534 | 0.2618 | 0.8234 | 4.00E-04 | 0.0301 | 0.209042 | 0.4088 |
| 5 | 30000 | 0.0001 | 0.6952 | 0.9216 | 0.0024 | 0.0224 | 0.6954 | 0.9502 | 4.00E-04 | 0.0093 | 0.4928 | 0.5411 |
| 5 | 50000 | 0.00003 | 0.296 | 0.7969 | 0.0025 | 0.0534 | 0.2618 | 0.8234 | 4.00E-04 | 0.0301 | 0.207441 | 0.4088 |
| 5 | 50000 | 0.00005 | 0.5906 | 0.8963 | 0.0027 | 0.0296 | 0.5876 | 0.9281 | 1.00E-04 | 0.0126 | 0.4527 | 0.5365 |
| 5 | 50000 | 0.0001 | 0.9181 | 0.9754 | 9.00E-04 | 0.0072 | 0.926 | 0.9873 | 1.00E-04 | 0.0024 | 0.5225 | 0.5280 |
| 6 | 20000 | 0.00003 | 0.0443 | 0.6851 | 4.00E-04 | 0.0453 | 0.0209 | 0.6856 | 0 | 0.0223 | 0.008848 | 0.1874 |
| 6 | 20000 | 0.00005 | 0.2099 | 0.7933 | 0.0011 | 0.0413 | 0.1586 | 0.8034 | 0 | 0.0226 | 0.09992 | 0.3754 |
| 6 | 20000 | 0.0001 | 0.6346 | 0.9242 | 8.00E-04 | 0.0192 | 0.6278 | 0.9467 | 0 | 0.0078 | 0.486 | 0.6233 |
| 6 | 30000 | 0.00003 | 0.1617 | 0.7716 | 7.00E-04 | 0.0425 | 0.1131 | 0.7781 | 0 | 0.0232 | 0.070684 | 0.3340 |
| 6 | 30000 | 0.00005 | 0.45 | 0.8757 | 0.0012 | 0.0294 | 0.4249 | 0.8996 | 1.00E-04 | 0.0153 | 0.3252 | 0.5411 |
| 6 | 30000 | 0.0001 | 0.8573 | 0.9696 | 0.001 | 0.0078 | 0.858 | 0.9816 | 1.00E-04 | 0.0021 | 0.6097 | 0.6580 |
| 6 | 50000 | 0.00003 | 0.45 | 0.8757 | 0.0012 | 0.0294 | 0.4249 | 0.8996 | 1.00E-04 | 0.0153 | 0.3255 | 0.5411 |
| 6 | 50000 | 0.00005 | 0.763 | 0.9547 | 0.0011 | 0.0117 | 0.7621 | 0.9718 | 1.00E-04 | 0.0043 | 0.561 | 0.6426 |
| 6 | 50000 | 0.0001 | 0.9778 | 0.9951 | 0 | 0.0012 | 0.9825 | 0.9977 | 0 | 2.00E-04 | 0.6767 | 0.6824 |
| 7 | 20000 | 0.00003 | 0.0903 | 0.7477 | 2.00E-04 | 0.0345 | 0.0447 | 0.7494 | 0 | 0.0156 | 0.019695 | 0.2570 |
| 7 | 20000 | 0.00005 | 0.326 | 0.8578 | 4.00E-04 | 0.0269 | 0.268 | 0.8675 | 0 | 0.0121 | 0.169085 | 0.4855 |
| 7 | 20000 | 0.0001 | 0.7721 | 0.9649 | 9.00E-04 | 0.0073 | 0.7702 | 0.9787 | 1.00E-04 | 0.0028 | 0.5801 | 0.7163 |
| 7 | 30000 | 0.00003 | 0.2595 | 0.8362 | 5.00E-04 | 0.029 | 0.1993 | 0.844 | 0 | 0.0146 | 0.12181 | 0.4356 |
| 7 | 30000 | 0.00005 | 0.5951 | 0.9317 | 7.00E-04 | 0.0144 | 0.5791 | 0.9493 | 0 | 0.0062 | 0.4315 | 0.6497 |
| 7 | 30000 | 0.0001 | 0.9384 | 0.9891 | 1.00E-04 | 0.002 | 0.9393 | 0.9939 | 0 | 5.00E-04 | 0.7009 | 0.7489 |
| 7 | 50000 | 0.00003 | 0.5951 | 0.9317 | 7.00E-04 | 0.0144 | 0.5791 | 0.9493 | 0 | 0.0062 | 0.4321 | 0.6497 |
| 7 | 50000 | 0.00005 | 0.8828 | 0.9822 | 1.00E-04 | 0.0041 | 0.883 | 0.9894 | 0 | 0.0016 | 0.6562 | 0.7379 |
| 7 | 50000 | 0.0001 | 0.995589415 | 0.998897354 | 0 | 0.000501203 | 0.996992783 | 0.999398557 | 0 | 0.000100241 | 0.794 | 0.7996 |
| 8 | 20000 | 0.00003 | 0.1399 | 0.8007 | 2.00E-04 | 0.0245 | 0.0852 | 0.8019 | 0 | 0.0116 | 0.036389 | 0.3361 |
| 8 | 20000 | 0.00005 | 0.4425 | 0.9044 | 1.00E-04 | 0.0168 | 0.3941 | 0.9166 | 0 | 0.0079 | 0.2402 | 0.5841 |
| 8 | 20000 | 0.0001 | 0.8721 | 0.9818 | 1.00E-04 | 0.0044 | 0.8714 | 0.9885 | 0 | 0.0014 | 0.6581 | 0.7929 |
| 8 | 30000 | 0.00003 | 0.37 | 0.886 | 1.00E-04 | 0.0201 | 0.3057 | 0.8945 | 0 | 0.0078 | 0.182473 | 0.5304 |
| 8 | 30000 | 0.00005 | 0.7202 | 0.9593 | 0 | 0.007 | 0.7104 | 0.9727 | 0 | 0.0032 | 0.5204 | 0.7419 |
| 8 | 30000 | 0.0001 | 0.9796 | 0.9961 | 1.00E-04 | 9.00E-04 | 0.9809 | 0.9979 | 0 | 3.00E-04 | 0.7835 | 0.8310 |
| 8 | 50000 | 0.00003 | 0.7202 | 0.9593 | 0 | 0.007 | 0.7104 | 0.9727 | 0 | 0.0032 | 0.521 | 0.7419 |
| 8 | 50000 | 0.00005 | 0.9483 | 0.9922 | 0 | 0.0013 | 0.949 | 0.9962 | 0 | 3.00E-04 | 0.7323 | 0.8126 |
| 8 | 50000 | 0.0001 | 0.999388442 | 0.999898074 | 0 | 0 | 0.999592294 | 1 | 0 | 0 | 0.8756 | 0.8807 |
| 9 | 20000 | 0.00003 | 0.2027 | 0.8463 | 0 | 0.0179 | 0.1389 | 0.8488 | 0 | 0.0083 | 0.057986 | 0.4129 |
| 9 | 20000 | 0.00005 | 0.5539 | 0.9357 | 0 | 0.0093 | 0.511 | 0.9455 | 0 | 0.0044 | 0.3123 | 0.6738 |
| 9 | 20000 | 0.0001 | 0.9303 | 0.9934 | 0 | 0.0012 | 0.9299 | 0.9961 | 0 | 6.00E-04 | 0.7127 | 0.8472 |
| 9 | 30000 | 0.00003 | 0.4739 | 0.9221 | 1.00E-04 | 0.0113 | 0.4201 | 0.9308 | 0 | 0.0049 | 0.2518 | 0.6242 |
| 9 | 30000 | 0.00005 | 0.8133 | 0.9785 | 1.00E-04 | 0.0037 | 0.8093 | 0.9865 | 0 | 0.0014 | 0.5904 | 0.8100 |
| 9 | 30000 | 0.0001 | 0.9912 | 0.9989 | 0 | 2.00E-04 | 0.9922 | 0.9994 | 0 | 1.00E-04 | 0.8385 | 0.8863 |
| 9 | 50000 | 0.00003 | 0.8133 | 0.9785 | 1.00E-04 | 0.0037 | 0.8093 | 0.9865 | 0 | 0.0014 | 0.5893 | 0.8100 |
| 9 | 50000 | 0.00005 | 0.9768 | 0.9973 | 0 | 2.00E-04 | 0.9773 | 0.999 | 0 | 1.00E-04 | 0.7917 | 0.8697 |
| 9 | 50000 | 0.0001 | 0.999670438 | 1 | 0 | 0 | 0.999890146 | 1 | 0 | 0 | 0.9306 | 0.9331 |
| 10 | 20000 | 0.00003 | 0.2734 | 0.8777 | 0 | 0.0138 | 0.198 | 0.8807 | 0 | 0.0062 | 0.086865 | 0.4917 |
| 10 | 20000 | 0.00005 | 0.6568 | 0.9606 | 3.00E-04 | 0.0052 | 0.625 | 0.9687 | 0 | 0.0027 | 0.3906 | 0.7473 |
| 10 | 20000 | 0.0001 | 0.97 | 0.9961 | 0 | 4.00E-04 | 0.97 | 0.9983 | 0 | 0 | 0.7552 | 0.8889 |
| 10 | 30000 | 0.00003 | 0.5758 | 0.9463 | 0 | 0.0061 | 0.5279 | 0.953 | 0 | 0.003 | 0.3145 | 0.7027 |
| 10 | 30000 | 0.00005 | 0.8892 | 0.9898 | 0 | 0.0017 | 0.8869 | 0.9935 | 0 | 4.00E-04 | 0.6362 | 0.8540 |
| 10 | 30000 | 0.0001 | 0.9964 | 0.9997 | 0 | 0 | 0.9972 | 1 | 0 | 0 | 0.8755 | 0.9235 |
| 10 | 50000 | 0.00003 | 0.8892 | 0.9898 | 0 | 0.0017 | 0.8869 | 0.9935 | 0 | 4.00E-04 | 0.6366 | 0.8540 |
| 10 | 50000 | 0.00005 | 0.9901 | 0.999 | 0 | 1.00E-04 | 0.9904 | 0.9999 | 0 | 0 | 0.8299 | 0.9095 |
| 10 | 50000 | 0.0001 | 1 | 1 | 0 | 0 | 1 | 1 | 0 | 0 | 0.9586 | 0.9638 |

† Power equals to the probability of meeting pathogenic ACMG/AMP evidence; LR ≥ 18.7 for at least strong pathogenic evidence and LR ≥ 2.08 for at least supporting pathogenic evidence.

‡ Type I error equals to the probability of meeting benign ACMG/AMP evidence; LR ≤ 0.053 for at least strong benign evidence and LR ≤ 0.48 for at least supporting benign evidence.

§ Power equals to the probability of meeting strong ACMG/AMP evidence (PS4 criterion, OR > 5.0, CI not including 1.0/p-value < 0.05)

LR, likelihood ratio. Genotype data simulations were carried out for variants conferring disease relative risk of 1, 2, 3, 4, 5, 6, 7, 8, 9 or 10, minor allele frequency of 0.0001, 0.00005 or 0.00003 and sample size of N = 20,000 (20,000 BC cases and 20,000 controls), 30,000 (30,000 BC cases and 30,000 controls) or 50,000 (50,000 BC cases and 50,000 controls). The number of permutations for each case scenario was 10,000 time

| **Gene** | **Variant_ID**  **(GRCh37/hg19)** | **HGVS**  **Nucleotide;**  **Protein** | **ClinVar Class** | **Total**  **Cases/**  **Controls** | **Case Carriers** | | **Control Carriers** | | **Logistic Regression analysis** | | **Case-control likelihood ratio Country-stratified analysis**  **[Dorling et al. 2021]** | |
| --- | --- | --- | --- | --- | --- | --- | --- | --- | --- | --- | --- | --- |
|  |  |  |  |  | **N (Frequency)** | **†Age** | **N (Frequency)** | **†Age** | **OR (95% CI)** | **P-value** | **LR** | **ACMG/AMP evidence strength PS4‡** |
| *BRCA1* | chr17_41196683_C_T | c.*1012A>G; p.? | - | 72564/50781 | 1 (1.38x10^-5^) | 55 | 1 (1.97x10^-5^) | 56 | 0.47 (0.02-8.14) | 0.60 | 0.09 | Benign Moderate |
|  | chr17_41197404_A_G | c.*291C>T; p.? | Uncertain | 72523/50754 | 8 (1.10x10^-4^) | 56.4±9.7 (38-69) | 12 (2.36x10^-4^) | 59.6±12 (27-74) | 0.45 (0.17-1.13) | 0.09 | 5.14x10^-9^ | Benign Very Strong |
|  | chr17_41267714_A_T | c.134+29T>A; p.? | - | 72453/50706 | 2 (2.76x10^-5^) | 38±1.4 (37-39) | 1 (1.97x10^-5^) | 42 | 1.07 (0.09-12.18) | 0.96 | 2.69 | No evidence§ |
|  | chr17_41245683_A_G | c.1865C>T; p.(Ala622Val) | Benign | 72545/50769 | 14 (1.93x10^-4^) | 58.1±11.2 (39-72) | 11 (2.17x10^-4^) | 53.6±12.7 (25-77) | 0.73 (0.32-1.65) | 0.46 | 7.28x10^-10^ | Benign Very Strong |
|  | chr17_41245577_C_T | c.1971A>G; p.(=) | Benign | 72561/50778 | 4 (5.51x10^-5^) | 47±6.9 (37-52) | 1 (1.97x10^-5^) | 56 | 3.89 (0.43-35.3) | 0.23 | 3.05 | No evidence§ |
|  | chr17_41245465_T_C | c.2083G>A; p.(Asp695Asn) | Conflicting | 72564/50782 | 4 (5.51x10^-5^) | 44.5±10.9 (32-58) | 7 (1.38x10^-4^) | 57.4±13.6 (38-75) | 0.49 (0.2-1.23) | 0.13 | 1.30x10^-4^ | Benign Very Strong |
|  | chr17_41244982_T_C | c.2566T>C; p.(Tyr856His) | Benign | 72565/50780 | 2 (2.76x10^-5^) | 59±0  (59-59) | 1 (1.97x10^-5^) | 55 | 1.47 (0.13-16.32) | 0.75 | 0.11 | Benign Moderate |
|  | chr17_41244886_A_G | c.2662C>T; p.(His888Tyr) | Conflicting | 72557/50774 | 4 (5.51x10^-5^) | 52.5±4.2 (47-57) | 5 (9.85x10^-5^) | 53.2±7.4 (46-65) | 0.62 (0.16-2.36) | 0.48 | 6.83x10^-5^ | Benign Very Strong |
|  | chr17_41243840_A_C | c.3708T>G; p.(Asn1236Lys) | Benign | 72562/50779 | 45 (6.20x10^-4^) | 57.9±10.8 (34-74) | 45 (8.86x10^-4^) | 54.8±10.4 (30-78) | 0.74 (0.48-1.13) | 0.17 | 4.93x10^-34^ | Benign Very Strong |
|  | chr17_41243655_GAA_INDEL | c.3891_3893delTTC; p.(Ser1298del) | Uncertain | 72563/50781 | - | - | 2 (3.94x10^-5^) | 43±1.4 (42-44) | 2.99x10^-4^  (1.05x10^-30^-8.53x10^22^) | 0.79 | 0.04 | Benign Strong |
|  | chr17_41243494_C_T | c.4054G>A; p.(Glu1352Lys) | Uncertain | 72557/50777 | 1 (1.38x10^-5^) | 65 | - | - | 3878.68  (1.49x10^-34^-1.01x10^41^) | 0.85 | 0.64 | No evidence |
|  | chr17_41243449_C_T | c.4096+3A>G; p.? | Uncertain | 72562/50778 | 4 (5.51x10^-5^) | 63.5±10.8 (51-74) | 1 (1.97x10^-5^) | 69 | 3.67 (0.4-33.58) | 0.25 | 0.20 | Benign Moderate |
|  | chr17_41243033_C_T | c.4113G>A; p.(=) | Likely benign | 72563/50781 | 8 (1.10x10^-4^) | 57.9±16.2 (33-80) | 1 (1.97x10^-5^) | 66 | 5.14 (0.63-41.51) | 0.12 | 0.56 | No evidence |
|  | chr17_41234451_A_G | c.4327C>T; p.(Arg1443*) | Pathogenic | 72558/50781 | 11 (1.52x10^-4^) | 41±8.8 (26-54) | 3 (5.91x10^-5^) | 63±8.7 (57-73) | 2.66 (0.73-9.7) | 0.14 | 526.71 | Pathogenic Very Strong |
|  | chr17_41251882_T_G | c.457A>C; p.(Ser153Arg) | Conflicting | 72559/50779 | 7 (9.65x10^-5^) | 49.9±12.5 (27-62) | 3 (5.91x10^-5^) | 57.7±11.4 (45-67) | 2.11 (0.53-8.41) | 0.29 | 0.36 | Benign Supporting |
|  | chr17_41226348_C_T | c.4675G>A; p.(Glu1559Lys) | Pathogenic | 72546/50770 | 1 (1.38x10^-5^) | 25 | 2 (3.94x10^-5^) | 47.5±13.4 (38-57) | 0.56 (0.04-6.35) | 0.64 | 0.23 | Benign Supporting |
|  | chr17_41219642_C_T | c.5057A>G; p.(His1686Arg) | (Likely) pathogenic | 72566/50779 | 1 (1.38x10^-5^) | 31 | - | - | 4288.82  (1.65x10^-34^-1.11x10^41^) | 0.85 | 5.92 | No evidence§ |
|  | chr17_41219619_C_G | c.5074+6C>G; p.? | Benign | 72525/50754 | 22 (3.03x10^-4^) | 55.7±12.1 (29-72) | 5 (9.85x10^-5^) | 56.6±10.7 (46-72) | 3.05 (1.14-8.14) | 0.03 | 1.05x10^-3^ | Benign Very Strong |
|  | chr17_41215947_T_G | c.5096G>T; p.(Arg1699Leu) | Uncertain | 72560/50780 | 17 (2.34x10^-4^) | 50.6±15.9 (24-76) | 3 (5.91x10^-5^) | 63.7±4.2 (59-67) | 3.8 (1.08-13.36) | 0.04 | 307.47 | Pathogenic Strong |
|  | chr17_41209164_C_T | c.5194-12G>A; p.? | Pathogenic | 72564/50782 | 1 (1.38x10^-5^) | 65 | - | - | 1484.71  (5.72x10^-35^-3.85x10^40^) | 0.87 | 0.52 | No evidence |
|  | chr17_41203189_A_G | c.5278-55C>T; p.? | - | 72559/50778 | 1 (1.38x10^-5^) | 28 | - | - | 2765.34  (1.07x10^-34^-7.18x10^40^) | 0.86 | 9.31 | No evidence§ |
|  | chr17_41203002_A_G | c.5332+78C>T; p.? | Likely benign | 72560/50780 | 8 (1.10x10^-4^) | 58.8±12.7 (37-71) | 4 (7.88x10^-5^) | 67.5±6.8 (62-76) | 1.4 (0.41-4.8) | 0.59 | 5.50x10^-3^ | Benign Strong |
|  | chr17_41201198_A_C | c.5346G>T; p.(Trp1782Cys) | Not Provided | 72560/50778 | - | - | 1 (1.97x10^-5^) | 55 | 2.59x10^-4^  (9.96x10^-42^-6.71x10^33^) | 0.85 | 0.25 | Benign Supporting |
|  | chr17_41247921_C_G | c.612G>C; p.(Leu204Phe) | Conflicting | 72320/50643 | 7 (9.68x10^-5^) | 59.9±5.8 (49-67) | 17 (3.36x10^-4^) | 57.5±10.2 (45-74) | 0.34 (0.14-0.84) | 0.02 | 4.23x10^-13^ | Benign Very Strong |
| *BRCA2* | chr13_32972834_A_INDEL | c.10184del; p.(Glu3395Glyfs*32) | Conflicting | 72549/50774 | 2 (2.76x10^-5^) | 56±12.7 (47-65) | - | - | 3901.4  (3.96x10^-21^-3.85x10^27^) | 0.77 | 1.47 | No evidence |
|  | chr13_32906711_G_T | c.1096T>G; p.(Leu366Val) | Conflicting | 72447/50706 | 1 (1.38x10^-5^) | 52 | 3 (5.92x10^-5^) | 54±22.9 (37-80) | 0.19 (0.02-1.98) | 0.17 | 0.04 | Benign Strong |
|  | chr13_32907078_G_T | c.1463T>G; p.(Ile488Arg) | Uncertain | 72559/50777 | 1 (1.38x10^-5^) | 74 | 4 (7.88x10^-5^) | 55.8±13 (38-66) | 0.17 (0.01-1.69) | 0.13 | 6.44x10^-3^ | Benign Strong |
|  | chr13_32907359_A_C | c.1744A>C; p.(Thr582Pro) | Benign | 72564/50781 | 1 (1.38x10^-5^) | 55 | 1 (1.97x10^-5^) | 65 | 1.8 (0.26-12.22) | 0.55 | 0.35 | Benign Supporting |
|  | chr13_32907403_C_T | c.1788T>C; p.(=) | Benign | 72548/50772 | 34 (4.69x10^-4^) | 58±13.5 (31-76) | 18 (3.55x10^-4^) | 54.6±16 (22-75) | 1.4 (0.8-2.48) | 0.23 | 3.02x10^-5^ | Benign Very Strong |
|  | chr13_32893377_T_G | c.231T>G; p.(=) | Benign | 72559/50774 | 2 (2.76x10^-5^) | 34.5±6.4 (30-39) | 2 (3.94x10^-5^) | 55±15.6 (44-66) | 0.61 (0.08-4.51) | 0.63 | 0.67 | No evidence |
|  | chr13_32911030_A_C | c.2538A>C; p.(=) | Benign | 72560/50780 | 18 (2.48x10^-4^) | 57.3±10.7 (33-73) | 8 (1.58x10^-4^) | 56.8±15.6 (30-74) | 1.56 (0.66-3.64) | 0.30 | 4.40x10^-3^ | Benign Strong |
|  | chr13_32911172_A_G | c.2680G>A; p.(Val894Ile) | Benign | 72557/50777 | 18 (2.48x10^-4^) | 54.2±11.3 (35-74) | 16 (3.15x10^-4^) | 54.3±7.7 (43-69) | 0.79 (0.4-1.57) | 0.51 | 2.76x10^-8^ | Benign Very Strong |
|  | chr13_32911565_A_G | c.3073A>G; p.(Lys1025Glu) | Conflicting | 72490/50734 | 15 (2.07x10^-4^) | 60.3±11.9 (35-74) | 7 (1.38x10^-4^) | 52.4±17.3 (22-67) | 1.33 (0.52-3.35) | 0.54 | 2.40x10^-4^ | Benign Very Strong |
|  | chr13_32911644_C_T | c.3152T>C; p.(Leu1051Ser) | Uncertain​ | 72557/50778 | 4 (5.51x10^-5^) | 67±7.7 (60-77) | - | - | 10099.37  (2.84x10^-27^-3.59x10^34^) | 0.80 | 0.54 | No evidence |
|  | chr13_32893467_A_G | c.316+5G>A; p.? | Pathogenic | 72564/50776 | 1 (1.38x10^-5^) | 40 | - | - | 4725.36  (1.82x10^-34^-1.23x10^41^) | 0.85 | 2.54 | No evidence§ |
|  | chr13_32911756_C_T | c.3264T>C; p.(=) | Benign | 72564/50780 | 11 (1.52x10^-4^) | 55.7±9.6 (39-68) | 6 (1.18x10^-4^) | 61.5±9.8 (46-72) | 1.38 (0.5-3.78) | 0.52 | 4.50x10^-3^ | Benign Strong |
|  | chr13_32912174_A_G | c.3682A>G; p.(Asn1228Asp) | Benign | 72533/50756 | 21 (2.90x10^-4^) | 57.7±9.9 (35-73) | 11 (2.17x10^-4^) | 48.5±11.4 (29-71) | 1.39 (0.66-2.92) | 0.38 | 2.90x10^-6^ | Benign Very Strong |
|  | chr13_32890548_C_T | c.-39-11C>T; p.? | Uncertain | 72565/50782 | - | - | 1 (1.97x10^-5^) | 45 | 2.66x10^-4^  (1.03x10^-41^-6.91x10^33^) | 0.85 | 0.37 | Benign Supporting |
|  | chr13_32890547_INDEL_TCT | c.-39-12_-39-10del; p.? | Conflicting | 72563/50781 | 3 (4.13x10^-5^) | 58.3±9.5 (51-69) | 6 (1.18x10^-4^) | 54.7±9.5 (44-68) | 0.33 (0.08-1.38) | 0.13 | 2.57x10^-4^ | Benign Very Strong |
|  | chr13_32890555_G_T | c.-39-4G>T; p.? | Benign | 72564/50779 | 1 (1.38x10^-5^) | 65 | 2 (3.94x10^-5^) | 57±5.7 (53-61) | 0.28 (0.02-3.33) | 0.32 | 0.04 | Benign Strong |
|  | chr13_32899354_A_G | c.425+33A>G; p.? | (Likely) benign | 72417/50701 | 46 (6.35x10^-4^) | 58.7±9.6 (39-74) | 40 (7.89x10^-4^) | 54.6±11.9 (21-78) | 0.83 (0.54-1.29) | 0.42 | 8.39x10^-20^ | Benign Very Strong |
|  | chr13_32912868_A_G | c.4376A>G; p.(Asn1459Ser) | Conflicting | 72472/50739 | 1 (1.38x10^-5^) | 67 | - | - | 3855.89 (1.49x10^-34^-1x10^41^) | 0.85 | 0.80 | No evidence |
|  | chr13_32913148_C_T | c.4656T>C; p.(=) | Likely benign | 72558/50780 | 9 (1.24x10^-4^) | 54.3±11.4 (33-67) | 13 (2.56x10^-4^) | 52.2±8.1 (41-67) | 0.48 (0.2-1.14) | 0.10 | 2.23x10^-7^ | Benign Very Strong |
|  | chr13_32913173_C_A | c.4681C>A; p.(His1561Asn) | Conflicting | 72560/50782 | 3 (4.13x10^-5^) | 63.3±4.7 (58-67) | 1 (1.97x10^-5^) | 70 | 2.63 (0.27-25.75) | 0.40 | 0.38 | Benign Supporting |
|  | chr13_32900313_C_T | c.475+26T>C; p.? | Likely benign | 72469/50740 | 7 (9.66x10^-5^) | 48.1±11.4 (33-61) | 2 (3.94x10^-5^) | 53.5±16.3 (42-65) | 2.37 (0.48-11.77) | 0.29 | 4.87 | No evidence§ |
|  | chr13_32900405_A_C | c.502C>A; p.(Pro168Thr) | Benign | 72558/50779 | 12 (1.65x10^-4^) | 57±11.4 (33-71) | 4 (7.88x10^-5^) | 56.8±12.7 (41-68) | 1.86 (0.58-5.88) | 0.29 | 9.31x10^-3^ | Benign Strong |
|  | chr13_32913910_A_G | c.5418A>G; p.(=) | Benign | 72543/50766 | 9 (1.24x10^-4^) | 55±9.5 (39-68) | 7 (1.38x10^-4^) | 59±11.1 (44-72) | 0.95 (0.35-2.59) | 0.93 | 8.24x10^-4^ | Benign Very Strong |
|  | chr13_32914127_A_G | c.5635G>A; p.(Glu1879Lys) | Conflicting | 72565/50780 | 14 (1.93x10^-4^) | 55.2±9.6 (38-66) | 10 (1.97x10^-4^) | 57.6±5.5 (48-67) | 1.29 (0.56-2.95) | 0.54 | 4.11x10^-4^ | Benign Very Strong |
|  | chr13_32900691_A_G | c.572A>G; p.(Asp191Gly) | Not Provided | 72563/50780 | 2 (2.76x10^-5^) | 54.5±31.8 (32-77) | - | - | 4261.53 (1.84x10^-23^-9.86x10^29^) | 0.79 | 3.53 | No evidence§ |
|  | chr13_32900694_C_T | c.575T>C; p.(Met192Thr) | Conflicting | 72555/50773 | 6 (8.27x10^-5^) | 59.3±6.4 (50-67) | - | - | 7422.57 (5.55x10^-21^-9.92x10^27^) | 0.75 | 0.44 | Benign Supporting |
|  | chr13_32900706_A_G | c.587G>A; p.(Ser196Asn) | Uncertain | 72565/50779 | 1 (1.38x10^-5^) | 67 | 1 (1.97x10^-5^) | 38 | 0.5 (0.02-9.09) | 0.64 | 0.26 | Benign Supporting |
|  | chr13_32914467_C_T | c.5975C>T; p.(Ser1992Leu) | Conflicting | 72560/50780 | 2 (2.76x10^-5^) | 60±9.9 (53-67) | 1 (1.97x10^-5^) | 45 | 1.49 (0.13-16.74) | 0.74 | 0.32 | Benign Supporting |
|  | chr13_32914470_C_T | c.5978T>C; p.(Leu1993Ser) | - | 72547/50766 | 3 (4.14x10^-5^) | 62±17.3 (43-77) | 1 (1.97x10^-5^) | 55 | 2.35 (0.23-24.06) | 0.47 | 0.30 | Benign Supporting |
|  | chr13_32915240_A_G | c.6748A>G; p.(Thr2250Ala) | Benign | 72566/50779 | 17 (2.34x10^-4^) | 54.1±10.5 (31-71) | 8 (1.58x10^-4^) | 61.4±7.9 (48-70) | 2.12 (0.9-4.96) | 0.08 | 0.14 | Benign Moderate |
|  | chr13_32903812_A_T | c.681+183T>A; p.? | - | 72485/50738 | 2 (2.76x10^-5^) | 59.5±10.6 (52-67) | - | - | 1509.26 (5.31x10^-24^-4.29x10^29^) | 0.81 | 0.58 | No evidence |
|  | chr13_32918701_A_C | c.6848C>A; p.(Pro2283His) | Uncertain | 72547/50769 | 3 (4.14x10^-5^) | 60±20 (40-80) | 1 (1.97x10^-5^) | 68 | 3.1 (0.3-31.51) | 0.34 | 1.20 | No evidence |
|  | chr13_32918769_C_G | c.6916G>C; p.(Ala2306Pro) | Uncertain | 72312/50628 | 3 (4.15x10^-5^) | 54.3±16.5 (38-71) | 2 (3.95x10^-5^) | 66±5.7 (62-70) | 0.97 (0.15-5.95) | 0.98 | 0.23 | Benign Supporting |
|  | chr13_32918788_A_T | c.6935A>T; p.(Asp2312Val) | Benign | 72417/50682 | 6 (8.29x10^-5^) | 55.5±16.3 (32-72) | 2 (3.95x10^-5^) | 59.5±3.5 (57-62) | 1.94 (0.38-9.83) | 0.42 | 0.24 | Benign Supporting |
|  | chr13_32919384_G_T | c.6937+594T>G; p.? | Benign | 72531/50758 | 42 (5.79x10^-4^) | 55.2±12.4 (30-79) | 16 (3.15x10^-4^) | 51.9±11.1 (37-71) | 1.64 (0.91-2.94) | 0.10 | 3.35x10^-4^ | Benign Very Strong |
|  | chr13_32921033_A_G | c.7007G>A; p.(Arg2336His) | Pathogenic | 72565/50781 | 4 (5.51x10^-5^) | 46±20.1 (28-74) | 3 (5.91x10^-5^) | 56±11.5 (47-69) | 1.17 (0.25-5.31) | 0.83 | 1.13 | No evidence |
|  | chr13_32929309_A_G | c.7319A>G; p.(His2440Arg) | Benign | 72565/50781 | 8 (1.10x10^-4^) | 55.2±9.8 (39-68) | 6 (1.18x10^-4^) | 61.5±9.8 (46-72) | 0.95 (0.32-2.79) | 0.94 | 3.69x10^-3^ | Benign Strong |
|  | chr13_32930598_T_C | c.7469T>C; p.(Ile2490Thr) | Benign | 72560/50777 | 35 (4.82x10^-4^) | 50.2±10.6 (31-69) | 19 (3.74x10^-4^) | 55.3±12.7 (31-73) | 1.41 (0.8-2.49) | 0.23 | 3.93x10^-3^ | Benign Strong |
|  | chr13_32931973_A_C | c.7712A>C; p.(Glu2571Ala) | - | 72559/50779 | 2 (2.76x10^-5^) | 59.5±7.8 (54-65) | 3 (5.91x10^-5^) | 49±3.6 (46-53) | 0.48 (0.07-3.09) | 0.44 | 0.01 | Benign Strong |
|  | chr13_32936671_A_G | c.7817A>G; p.(Asp2606Gly) | - | 72554/50777 | 4 (5.51x10^-5^) | 64.5±13.7 (44-73) | - | - | 1.01 (0.21-4.74) | 0.98 | 0.99 | No evidence |
|  | chr13_32936680_A_G | c.7826G>A; p.(Gly2609Asp) | Likely pathogenic | 72563/50781 | 2 (2.76x10^-5^) | 51.5±0.7 (51-52) | 1 (1.97x10^-5^) | 56 | 0.87 (0.07-10.42) | 0.92 | 0.20 | Benign Moderate |
|  | chr13_32936722_A_G | c.7868A>G; p.(His2623Arg) | (Likely) pathogenic | 72545/50765 | 5 (6.89x10^-5^) | 47.8±10.6 (35-64) | 2 (3.94x10^-5^) | 56.5±16.3 (45-68) | 1.51 (0.28-8.01) | 0.63 | 0.88 | No evidence |
|  | chr13_32936724_C_T | c.7870T>C; p.(Tyr2624His) | - | 72561/50779 | 3 (4.13x10^-5^) | 57.3±14.2 (41-67) | - | - | 14223.95 (1.30x10^-31^-1.55x10^39^) | 0.82 | 2.56 | No evidence§ |
|  | chr13_32936725_A_G | c.7871A>G; p.(Tyr2624Cys) | Uncertain | 72533/50769 | 3 (4.14x10^-5^) | 45.7±6.1 (39-51) | 2 (3.94x10^-5^) | 62.5±0.7 (62-63) | 1.06 (0.16-6.71) | 0.95 | 1.10 | No evidence |
|  | chr13_32936742_A_C | c.7888A>C; p.(Lys2630Gln) | Likely pathogenic | 72544/50775 | - | - | 3 (5.91x10^-5^) | 55.7±10.2 (44-63) | 3.61x10^-5^ (1.39x10^-39^-9.37x10^29^) | 0.80 | 0.01 | Benign Strong |
|  | chr13_32936782_C_G | c.7928C>G; p.(Ala2643Gly) | Conflicting | 72409/50694 | 3 (4.14x10^-5^) | 53.3±7 (46-60) | 3 (5.92x10^-5^) | 58.7±6.7 (51-63) | 0.54 (0.1-2.81) | 0.47 | 0.04 | Benign Strong |
|  | chr13_32937333_A_G | c.7994A>G; p.(Asp2665Gly) | Benign | 72555/50775 | 33 (4.55x10^-4^) | 59±11.6 (29-79) | 27 (5.32x10^-4^) | 50.8±14.4 (23-80) | 0.88 (0.52-1.48) | 0.65 | 3.51x10^-13^ | Benign Very Strong |
|  | chr13_32906415_A_G | c.800G>A; p.(Gly267Glu) | Conflicting | 72553/50778 | 7 (9.65x10^-5^) | 50±7.7 (39-63) | 2 (3.94x10^-5^) | 51±12.7 (42-60) | 2.24 (0.46-10.93) | 0.32 | 3.09 | No evidence§ |
|  | chr13_32937396_C_T | c.8057T>C; p.(Leu2686Pro) | (Likely) pathogenic | 72547/50767 | 2 (2.76x10^-5^) | 58.5±7.8 (53-64) | 1 (1.97x10^-5^) | 68 | 1.16 (0.1-13.33) | 0.90 | 0.17 | Benign Moderate |
|  | **chr13_32937506_C_G** | **c.8167G>C; p.(Asp2723His)** | **Pathogenic** | **72392/50680** | **18 (2.49x10^-4^)** | **48.5±10.9 (27-74)** | **1 (1.97x10^-5^)** | **64** | **12.3 (1.65-91.23)** | **1.41x10^-2^** | **8193.33** | **Pathogenic Very Strong** |
|  | chr13_32937582_A_G | c.8243G>A; p.(Gly2748Asp) | Pathogenic | 72565/50780 | 2 (2.76x10^-5^) | 45.5±2.1 (44-47) | - | - | 10986.93 (4.82x10^-36^-2.51x10^43^) | 0.84 | 3.60 | No evidence§ |
|  | chr13_32906446_T_G | c.831T>G; p.(Asn277Lys) | Conflicting | 72558/50776 | 20 (2.76x10^-4^) | 58±14.1 (33-78) | 12 (2.36x10^-4^) | 55.5±14.5 (22-71) | 1.04 (0.5-2.18) | 0.90 | 6.16x10^-6^ | Benign Very Strong |
|  | chr13_32944549_A_T | c.8342A>T; p.(Asn2781Ile) | Conflicting | 72384/50656 | 4 (5.53x10^-5^) | 61.5±9.4 (50-73) | 3 (5.92x10^-5^) | 60±10.8 (51-72) | 0.93 (0.2-4.16) | 0.93 | 7.12x10^-3^ | Benign Strong |
|  | chr13_32944563_A_G | c.8356G>A; p.(Ala2786Thr) | Conflicting | 72565/50781 | 3 (4.13x10^-5^) | 49±18.3 (29-65) | 1 (1.97x10^-5^) | 52 | 2.09 (0.2-21.36) | 0.53 | 1.19 | No evidence |
|  | chr13_32944713_A_G | c.8487+19A>G; p.? | Benign | 72545/50773 | 13 (1.79x10^-4^) | 57.4±10.8 (33-71) | 12 (2.36x10^-4^) | 63.6±9.2 (43-75) | 0.77 (0.34-1.72) | 0.53 | 1.59x10^-6^ | Benign Very Strong |
|  | chr13_32953453_A_G | c.8755-1G>A; p.? | Pathogenic | 72562/50776 | 3 (4.13x10^-5^) | 31.7±1.2 (31-33) | - | - | 6378.44 (2.58x10^-31^-1.57x10^38^) | 0.83 | 41.18 | Pathogenic Strong |
|  | chr13_32953473_A_G | c.8774A>G; p.(Gln2925Arg) | Uncertain | 72561/50781 | 2 (2.76x10^-5^) | 51.5±2.1 (50-53) | - | - | 4295.27 (1.60x10^-23^-1.16x10^30^) | 0.79 | 1.53 | No evidence |
|  | chr13_32953529_A_T | c.8830A>T; p.(Ile2944Phe) | Benign | 72510/50745 | 9 (1.24x10^-4^) | 50.4±8.8 (39-66) | 9 (1.77x10^-4^) | 52.6±7 (38-59) | 0.77 (0.3-1.98) | 0.60 | 3.89x10^-4^ | Benign Very Strong |
|  | **chr13_32954180_C_T** | **c.9154C>T; p.(Arg3052Trp)** | **Pathogenic** | **72563/50779** | **10 (1.38x10^-4^)** | **49.1±15.6 (30-78)** | **1 (1.97x10^-5^)** | **56** | **8.32 (1.04-66.48)** | **4.57x10^-2^** | **86.82** | **Pathogenic Strong** |
|  | chr13_32954340_A_T | c.9256+58A>T; p.? | Likely benign​ | 72345/50651 | 24 (3.32x10^-4^) | 53.9±12.2 (27-75) | 14 (2.76x10^-4^) | 52.6±10.5 (31-66) | 1.1 (0.56-2.16) | 0.77 | 8.70x10^-7^ | Benign Very Strong |
|  | chr13_32968940_A_T | c.9371A>T; p.(Asn3124Ile) | Pathogenic | 72548/50755 | 16 (2.21x10^-4^) | 50.3±10.5 (38-74) | - | - | 23417.61 (1.55x10^-20^-3.53x10^28^) | 0.72 | 3530.99 | Pathogenic Very Strong |
|  | chr13_32969073_A_T | c.9501+3A>T; p.? | Benign | 72442/50723 | 25 (3.45x10^-4^) | 53.6±11.1 (32-76) | 22 (4.34x10^-4^) | 57.6±10.8 (32-73) | 0.81 (0.45-1.47) | 0.50 | 6.12x10^-9^ | Benign Very Strong |
|  | chr13_32969079_A_C | c.9501+9A>C; p.? | Benign | 72550/50761 | 25 (3.45x10^-4^) | 58.4±10.5 (38-76) | 13 (2.56x10^-4^) | 49.1±14.1 (25-74) | 1.31 (0.65-2.63) | 0.44 | 4.01x10^-7^ | Benign Very Strong |
|  | chr13_32971023_C_T | c.9502-12T>C; p.? | (Likely) benign | 72494/50742 | 30 (4.14x10^-4^) | 56.2±11.9 (26-78) | 30 (5.91x10^-4^) | 52±8.5 (29-74) | 0.71 (0.42-1.19) | 0.20 | 3.54x10^-16^ | Benign Very Strong |
|  | chr13_32971042_A_G | c.9509A>G; p.(Asp3170Gly) | Benign | 72523/50748 | 6 (8.27x10^-5^) | 48.7±14 (32-65) | 3 (5.91x10^-5^) | 52±21.7 (29-72) | 1.52 (0.37-6.1) | 0.55 | 0.78 | No evidence |
|  | chr13_32971125_C_T | c.9592T>C; p.(Cys3198Arg) | Benign | 72506/50747 | 12 (1.66x10^-4^) | 53.8±12 (37-78) | 6 (1.18x10^-4^) | 54.5±10.8 (38-68) | 1.53 (0.57-4.11) | 0.39 | 0.05 | Benign Moderate |
|  | chr13_32972279_C_T | c.9649-20C>T; p.? | Benign | 72559/50780 | 13 (1.79x10^-4^) | 55.3±13.2 (34-75) | 9 (1.77x10^-4^) | 55.4±6.7 (47-65) | 0.99 (0.41-2.37) | 0.98 | 2.43x10^-4^ | Benign Very Strong |
|  | chr13_32906579_A_C | c.964A>C; p.(Lys322Gln) | Conflicting | 72483/50722 | 2 (2.76x10^-5^) | 56.5±13.4 (47-66) | 1 (1.97x10^-5^) | 67 | 1.56 (0.14-17.25) | 0.72 | 0.50 | No evidence |

Odds ratios and p-values were estimated using logistic regression analysis. Case-control likelihood ratios are estimated using the case-control likelihood ratio method.

ACMG/AMP, American College of Medical Genetics and Genomics (ACMG) and Association for Molecular Pathology (AMP); CI, confidence interval; LR, Likelihood ratio; OR, odds ratio. Variant nomenclature according to: *BRCA1* (NM_007294.4, NP_009225.1), *BRCA2* (NM_000059.3, NP_000050.2). Significant risks (p.value < 0.05, OR > 1.0, CI not including 1) of breast cancer are indicated in bold. P-values were estimated using the likelihood-ratio test.

† Age: mean±sd (range)

‡Aligned ACMG/AMP evidence strengths are modified following cut-offs resulted from the analysis of simulated datasets; Moderate and Supporting evidence strengths in favor of pathogenicity are noted as "No evidence
