# Supplementary material for: A Likelihood Ratio Approach for Utilizing Case-Control Data in the Clinical Classification of Rare Sequence Variants: Application to BRCA1 and BRCA2
Source: Hum Mutat. 2023 Sep 14;2023:9961341. doi: 10.1155/2023/9961341 (PMC11080979; doi:10.1155/2023/9961341)
Supplement: Supplementary 3 — Supplementary Table S5: Case-control likelihood ratios and evidence for assignment to ACMG/AMP code strengths for the 92 BRCA1 and BRCA2 variants using country-specific analyses with different penetrance models. [file 9961341.f3.pdf]

**A likelihood ratio approach for utilizing case-control data in the clinical classification of rare sequence variants: application to *BRCA1* and *BRCA2***

**Supplementary Table S5.** Case-control likelihood ratios and evidence for assignment to ACMG/AMP code strengths for the 92 *BRCA1* and *BRCA2* variants, using country-specific analyses with different penetrance models

| | | | | | | | Case Carriers | | Control Carriers | | Country-stratified analysis (main analysis) [Dorling et al. 2021] | | Country-stratified analysis using country-specific incidences [Dorling et al. 2021] | | Country-stratified analysis [Kuchenbaecker et al. 2017] | | Country-stratified analysis [Antoniou et al. 2003] | | Country-stratified analysis [Li et al. 2021] | | Country-unstratified analysis [Dorling et al. 2021] | |
|---|---|---|---|---|---|---|---|---|---|---|---|---|---|---|---|---|---|---|---|---|---|---|
| Gene | Variant_ID (GRCh37/hg19) | HGVS nucleotide | HGVS protein | ClinVar Class | Total cases | Total controls | N (Frequency) | †Age | N (Frequency) | †Age | LR | ACMG/AMP evidence strength PS4‡ | LR | ACMG/AMP evidence strength PS4‡ | LR | ACMG/AMP evidence strength PS4‡ | LR | ACMG/AMP evidence strength PS4‡ | LR | ACMG/AMP evidence strength PS4‡ | LR | ACMG/AMP evidence strength PS4‡ |
| *BRCA1* | chr17_41196683_C_T | c.*1012A>G | p.? | - | 72564 | 50781 | 1 ($1.38\times10^{-5}$) | 55 | 1 ($1.97\times10^{-5}$) | 56 | 0.09 | Benign Moderate | 0.09 | Benign Moderate | 0.03 | Benign Strong | 0.01 | Benign Strong | 0.17 | Benign Moderate | 0.14 | Benign Moderate |
| *BRCA1* | chr17_41197404_A_G | c.*291C>T | p.? | Uncertain | 72523 | 50754 | 8 ($1.10\times10^{-4}$) | 56.4±9.7 (38-69) | 12 ($2.36\times10^{-4}$) | 59.6±12 (27-74) | $5.14\times10^{-9}$ | Benign Very Strong | $3.11\times10^{-9}$ | Benign Very Strong | $2.65\times10^{-12}$ | Benign Very Strong | $9.56\times10^{-13}$ | Benign Very Strong | $1.96\times10^{-7}$ | Benign Very Strong | $1.82\times10^{-9}$ | Benign Very Strong |
| *BRCA1* | chr17_41267714_A_T | c.134+29T>A | p.? | - | 72453 | 50706 | 2 ($2.76\times10^{-5}$) | 38±1.4 (37-39) | 1 ($1.97\times10^{-5}$) | 42 | 2.69 | No evidence§ | 2.70 | No evidence§ | 1.93 | No evidence | 1.74 | No evidence | 1.74 | No evidence | 5.92 | No evidence§ |
| *BRCA1* | chr17_41245683_A_G | c.1865C>T | p.(Ala622Val) | Benign | 72545 | 50769 | 14 ($1.93\times10^{-4}$) | 58.1±11.2 (39-72) | 11 ($2.17\times10^{-4}$) | 53.6±12.7 (25-77) | $7.28\times10^{-10}$ | Benign Very Strong | $2.21\times10^{-10}$ | Benign Very Strong | $5.41\times10^{-13}$ | Benign Very Strong | $5.38\times10^{-15}$ | Benign Very Strong | $2.96\times10^{-7}$ | Benign Very Strong | $5.72\times10^{-9}$ | Benign Very Strong |
| *BRCA1* | chr17_41245577_C_T | c.1971A>G | p.(=) | Benign | 72561 | 50778 | 4 ($5.51\times10^{-5}$) | 47±6.9 (37-52) | 1 ($1.97\times10^{-5}$) | 56 | 3.05 | No evidence§ | 3.63 | No evidence§ | 1.42 | No evidence | 0.69 | No evidence | 4.11 | No evidence§ | 1.44 | No evidence |
| *BRCA1* | chr17_41245465_T_C | c.2083G>A | p.(Asp695Asn) | Conflicting | 72564 | 50782 | 4 ($5.51\times10^{-5}$) | 44.5±10.9 (32-58) | 7 ($1.38\times10^{-4}$) | 57.4±13.6 (38-75) | $1.30\times10^{-4}$ | Benign Very Strong | $9.76\times10^{-5}$ | Benign Very Strong | $2.07\times10^{-6}$ | Benign Very Strong | $1.24\times10^{-5}$ | Benign Very Strong | $1.11\times10^{-3}$ | Benign Very Strong | $6.68\times10^{-5}$ | Benign Very Strong |
| *BRCA1* | chr17_41244982_T_C | c.2566T>C | p.(Tyr856His) | Benign | 72565 | 50780 | 2 ($2.76\times10^{-5}$) | 59±0 (59-59) | 1 ($1.97\times10^{-5}$) | 55 | 0.11 | Benign Moderate | 0.11 | Benign Moderate | 0.04 | Benign Strong | 0.01 | Benign Strong | 0.23 | Benign Moderate | 0.11 | Benign Moderate |
| *BRCA1* | chr17_41244886_A_G | c.2662C>T | p.(His888Tyr) | Conflicting | 72557 | 50774 | 4 ($5.51\times10^{-5}$) | 52.5±4.2 (47-57) | 5 ($9.85\times10^{-5}$) | 53.2±7.4 (46-65) | $6.83\times10^{-5}$ | Benign Very Strong | $7.11\times10^{-5}$ | Benign Very Strong | $2.16\times10^{-6}$ | Benign Very Strong | $8.54\times10^{-8}$ | Benign Very Strong | $6.33\times10^{-4}$ | Benign Very Strong | $1.23\times10^{-4}$ | Benign Very Strong |
| *BRCA1* | chr17_41243840_A_C | c.3708T>G | p.(Asn1236Lys) | Benign | 72562 | 50779 | 45 ($6.20\times10^{-4}$) | 57.9±10.8 (34-74) | 45 ($8.86\times10^{-4}$) | 54.8±10.4 (30-78) | $4.93\times10^{-34}$ | Benign Very Strong | $2.77\times10^{-35}$ | Benign Very Strong | $8.53\times10^{-48}$ | Benign Very Strong | $6.28\times10^{-53}$ | Benign Very Strong | $1.94\times10^{-25}$ | Benign Very Strong | - | - |
| *BRCA1* | chr17_41243655_GAA_INDEL | c.3891_3893delTTC | p.(Ser1298del) | Uncertain | 72563 | 50781 | - | - | 2 ($3.94\times10^{-5}$) | 43±1.4 (42-44) | 0.04 | Benign Strong | 0.04 | Benign Strong | 0.02 | Benign Strong | $6.61\times10^{-3}$ | Benign Strong | 0.05 | Benign Moderate | 0.02 | Benign Strong |
| *BRCA1* | chr17_41243494_C_T | c.4054G>A | p.(Glu1352Lys) | Uncertain | 72557 | 50777 | 1 ($1.38\times10^{-5}$) | 65 | - | - | 0.64 | No evidence | 0.64 | No evidence | 0.59 | No evidence | 0.58 | No evidence | 0.83 | No evidence | 0.73 | No evidence |
| *BRCA1* | chr17_41243449_C_T | c.4096+3A>G | p.? | Uncertain | 72562 | 50778 | 4 ($5.51\times10^{-5}$) | 63.5±10.8 (51-74) | 1 ($1.97\times10^{-5}$) | 69 | 0.20 | Benign Moderate | 0.19 | Benign Moderate | 0.09 | Benign Moderate | 0.02 | Benign Strong | 0.32 | Benign Supporting | 0.06 | Benign Moderate |
| *BRCA1* | chr17_41243033_C_T | c.4113G>A | p.(=) | Likely benign | 72563 | 50781 | 8 ($1.10\times10^{-4}$) | 57.9±16.2 (33-80) | 1 ($1.97\times10^{-5}$) | 66 | 0.56 | No evidence | 0.39 | Benign Supporting | 0.23 | Benign Moderate | 0.05 | Benign Strong | 1.51 | No evidence | 0.74 | No evidence |
| *BRCA1* | chr17_41234451_A_G | c.4327C>T | p.(Arg1443*) | Pathogenic | 72558 | 50781 | 11 ($1.52\times10^{-4}$) | 41±8.8 (26-54) | 3 ($5.91\times10^{-5}$) | 63±8.7 (57-73) | 526.71 | Pathogenic Very Strong | 879.97 | Pathogenic Very Strong | 72.34 | Pathogenic Strong | 125.77 | Pathogenic Strong | 348.42 | Pathogenic Strong | 336.34 | Pathogenic Strong |
| *BRCA1* | chr17_41251882_T_G | c.457A>C | p.(Ser153Arg) | Conflicting | 72559 | 50779 | 7 ($9.65\times10^{-5}$) | 49.9±12.5 (27-62) | 3 ($5.91\times10^{-5}$) | 57.7±11.4 (45-67) | 0.36 | Benign Supporting | 0.24 | Benign Supporting | 0.14 | Benign Moderate | $9.38\times10^{-3}$ | Benign Strong | 0.64 | No evidence | 0.08 | Benign Moderate |
| *BRCA1* | chr17_41226348_C_T | c.4675G>A | p.(Glu1559Lys) | Pathogenic | 72546 | 50770 | 1 ($1.38\times10^{-5}$) | 25 | 2 ($3.94\times10^{-5}$) | 47.5±13.4 (38-57) | 0.23 | Benign Supporting | 0.19 | Benign Moderate | 0.15 | Benign Moderate | 0.05 | Benign Strong | 0.38 | Benign Supporting | 0.29 | Benign Supporting |
| *BRCA1* | chr17_41219642_C_T | c.5057A>G | p.(His1686Arg) | (Likely) pathogenic | 72566 | 50779 | 1 ($1.38\times10^{-5}$) | 31 | - | - | 5.92 | No evidence§ | 5.92 | No evidence§ | 6.40 | No evidence§ | 7.54 | No evidence§ | 4.06 | No evidence§ | 6.74 | No evidence§ |
| *BRCA1* | chr17_41219619_C_G | c.5074+6C>G | p.? | Benign | 72525 | 50754 | 22 ($3.03\times10^{-4}$) | 55.7±12.1 (29-72) | 5 ($9.85\times10^{-5}$) | 56.6±10.7 (46-72) | $1.05\times10^{-3}$ | Benign Very Strong | $3.18\times10^{-4}$ | Benign Very Strong | $1.81\times10^{-5}$ | Benign Very Strong | $8.68\times10^{-6}$ | Benign Very Strong | 0.05 | Benign Moderate | $2.32\times10^{-3}$ | Benign Very Strong |
| *BRCA1* | chr17_41215947_T_G | c.5096G>T | p.(Arg1699Leu) | Uncertain | 72560 | 50780 | 17 ($2.34\times10^{-4}$) | 50.6±15.9 (24-76) | 3 ($5.91\times10^{-5}$) | 63.7±4.2 (59-67) | 307.47 | Pathogenic Strong | 304.28 | Pathogenic Strong | 63.34 | Pathogenic Strong | 2.30 | No evidence§ | 256.46 | Pathogenic Strong | 255.53 | Pathogenic Strong |
| *BRCA1* | chr17_41209164_C_T | c.5194-12G>A | p.? | Pathogenic | 72564 | 50782 | 1 ($1.38\times10^{-5}$) | 65 | - | - | 0.52 | No evidence | 0.49 | No evidence | 0.47 | Benign Supporting | 0.49 | No evidence | 0.67 | No evidence | 0.73 | No evidence |
| *BRCA1* | chr17_41203189_A_G | c.5278-55C>T | p.? | - | 72559 | 50778 | 1 ($1.38\times10^{-5}$) | 28 | - | - | 9.31 | No evidence§ | 8.23 | No evidence§ | 19.00 | Pathogenic Strong | 6.78 | No evidence§ | 4.54 | No evidence§ | 10.05 | No evidence§ |
| *BRCA1* | chr17_41203002_A_G | c.5332+78C>T | p.? | Likely benign | 72560 | 50780 | 8 ($1.10\times10^{-4}$) | 58.8±12.7 (37-71) | 4 ($7.88\times10^{-5}$) | 67.5±6.8 (62-76) | $5.50\times10^{-3}$ | Benign Strong | $2.19\times10^{-3}$ | Benign Strong | $4.22\times10^{-4}$ | Benign Very Strong | $1.52\times10^{-3}$ | Benign Very Strong | 0.02 | Benign Strong | $1.72\times10^{-3}$ | Benign Very Strong |
| *BRCA1* | chr17_41201198_A_C | c.5346G>T | p.(Trp1782Cys) | Not Provided | 72560 | 50778 | - | - | 1 ($1.97\times10^{-5}$) | 55 | 0.25 | Benign Supporting | 0.25 | Benign Supporting | 0.14 | Benign Moderate | 0.07 | Benign Moderate | 0.36 | Benign Supporting | 0.16 | Benign Moderate |
| *BRCA1* | chr17_41247921_C_G | c.612G>C | p.(Leu204Phe) | Conflicting | 72320 | 50643 | 7 ($9.68\times10^{-5}$) | 59.9±5.8 (49-67) | 17 ($3.36\times10^{-4}$) | 57.5±10.2 (45-74) | $4.23\times10^{-13}$ | Benign Very Strong | $2.92\times10^{-13}$ | Benign Very Strong | $8.97\times10^{-17}$ | Benign Very Strong | $8.46\times10^{-19}$ | Benign Very Strong | $5.10\times10^{-11}$ | Benign Very Strong | $1.02\times10^{-14}$ | Benign Very Strong |
| *BRCA2* | chr13_32972834_A_INDEL | c.10184del | p.(Glu3395Glyfs*32) | Conflicting | 72549 | 50774 | 2 ($2.76\times10^{-5}$) | 56±12.7 (47-65) | - | - | 1.47 | No evidence | 1.41 | No evidence | 1.22 | No evidence | 1.38 | No evidence | 1.88 | No evidence | 1.76 | No evidence |
| *BRCA2* | chr13_32906711_G_T | c.1096T>G | p.(Leu366Val) | Conflicting | 72447 | 50706 | 1 ($1.38\times10^{-5}$) | 52 | 3 ($5.92\times10^{-5}$) | 54±22.9 (37-80) | 0.04 | Benign Strong | 0.03 | Benign Strong | 0.01 | Benign Strong | 0.04 | Benign Strong | 0.05 | Benign Strong | 0.04 | Benign Strong |
| *BRCA2* | chr13_32907078_G_T | c.1463T>G | p.(Ile488Arg) | Uncertain | 72559 | 50777 | 1 ($1.38\times10^{-5}$) | 74 | 4 ($7.88\times10^{-5}$) | 55.8±13 (38-66) | $6.44\times10^{-3}$ | Benign Strong | $6.22\times10^{-3}$ | Benign Strong | $2.88\times10^{-4}$ | Benign Very Strong | $2.17\times10^{-4}$ | Benign Very Strong | $8.52\times10^{-3}$ | Benign Strong | $4.57\times10^{-3}$ | Benign Strong |
| *BRCA2* | chr13_32907359_A_C | c.1744A>C | p.(Thr582Pro) | Benign | 72564 | 50781 | 1 ($1.38\times10^{-5}$) | 55 | 1 ($1.97\times10^{-5}$) | 65 | 0.35 | Benign Supporting | 0.32 | Benign Supporting | 0.11 | Benign Moderate | 0.09 | Benign Moderate | 0.31 | Benign Supporting | 0.32 | Benign Supporting |
| *BRCA2* | chr13_32907403_C_T | c.1788T>C | p.(=) | Benign | 72548 | 50772 | 34 ($4.69\times10^{-4}$) | 58±13.5 (31-76) | 18 ($3.55\times10^{-4}$) | 54.6±16 (22-75) | $3.02\times10^{-5}$ | Benign Very Strong | $1.10\times10^{-5}$ | Benign Very Strong | $6.94\times10^{-12}$ | Benign Very Strong | $1.01\times10^{-7}$ | Benign Very Strong | $1.51\times10^{-4}$ | Benign Very Strong | $1.54\times10^{-7}$ | Benign Very Strong |
| *BRCA2* | chr13_32893377_T_G | c.231T>G | p.(=) | Benign | 72559 | 50774 | 2 ($2.76\times10^{-5}$) | 34.5±6.4 (30-39) | 2 ($3.94\times10^{-5}$) | 55±15.6 (44-66) | 0.67 | No evidence | 0.58 | No evidence | 0.21 | Benign Moderate | 0.23 | Benign Supporting | 0.43 | Benign Supporting | 1.29 | No evidence |
| *BRCA2* | chr13_32911030_A_C | c.2538A>C | p.(=) | Benign | 72560 | 50780 | 18 ($2.48\times10^{-4}$) | 57.3±10.7 (33-73) | 8 ($1.58\times10^{-4}$) | 56.8±15.6 (30-74) | $4.40\times10^{-3}$ | Benign Strong | $3.62\times10^{-3}$ | Benign Strong | $1.55\times10^{-6}$ | Benign Very Strong | $1.23\times10^{-4}$ | Benign Very Strong | $5.03\times10^{-3}$ | Benign Strong | $1.06\times10^{-3}$ | Benign Very Strong |
| *BRCA2* | chr13_32911172_A_G | c.2680G>A | p.(Val894Ile) | Benign | 72557 | 50777 | 18 ($2.48\times10^{-4}$) | 54.2±11.3 (35-74) | 16 ($3.15\times10^{-4}$) | 54.3±7.7 (43-69) | $2.76\times10^{-8}$ | Benign Very Strong | $3.07\times10^{-8}$ | Benign Very Strong | $2.16\times10^{-16}$ | Benign Very Strong | $3.05\times10^{-13}$ | Benign Very Strong | $2.44\times10^{-7}$ | Benign Very Strong | $4.42\times10^{-8}$ | Benign Very Strong |
| *BRCA2* | chr13_32911565_A_G | c.3073A>G | p.(Lys1025Glu) | Conflicting | 72490 | 50734 | 15 ($2.07\times10^{-4}$) | 60.3±11.9 (35-74) | 7 ($1.38\times10^{-4}$) | 52.4±17.3 (22-67) | $2.40\times10^{-4}$ | Benign Very Strong | $1.71\times10^{-4}$ | Benign Very Strong | $1.54\times10^{-7}$ | Benign Very Strong | $3.21\times10^{-7}$ | Benign Very Strong | $8.15\times10^{-4}$ | Benign Very Strong | $4.17\times10^{-4}$ | Benign Very Strong |
| *BRCA2* | chr13_32911644_C_T | c.3152T>C | p.(Leu1051Ser) | Uncertain | 72557 | 50778 | 4 ($5.51\times10^{-5}$) | 67±7.7 (60-77) | - | - | 0.54 | No evidence | 0.43 | Benign Supporting | 0.29 | Benign Supporting | 0.42 | Benign Supporting | 0.92 | No evidence | 0.50 | No evidence |
| *BRCA2* | chr13_32893467_A_G | c.316+5G>A | p.? | Pathogenic | 72564 | 50776 | 1 ($1.38\times10^{-5}$) | 40 | - | - | 2.54 | No evidence§ | 2.63 | No evidence§ | 2.84 | No evidence§ | 2.85 | No evidence§ | 2.68 | No evidence§ | 2.19 | No evidence§ |
| *BRCA2* | chr13_32911756_C_T | c.3264T>C | p.(=) | Benign | 72564 | 50780 | 11 ($1.52\times10^{-4}$) | 55.7±9.6 (39-68) | 6 ($1.18\times10^{-4}$) | 61.5±9.8 (46-72) | $4.50\times10^{-3}$ | Benign Strong | $3.10\times10^{-3}$ | Benign Strong | $1.91\times10^{-6}$ | Benign Very Strong | $6.75\times10^{-5}$ | Benign Very Strong | $8.65\times10^{-3}$ | Benign Strong | $3.42\times10^{-3}$ | Benign Strong |
| *BRCA2* | chr13_32912174_A_G | c.3682A>G | p.(Asn1228Asp) | Benign | 72533 | 50756 | 21 ($2.90\times10^{-4}$) | 57.7±9.9 (35-73) | 11 ($2.17\times10^{-4}$) | 48.5±11.4 (29-71) | $2.90\times10^{-6}$ | Benign Very Strong | $2.05\times10^{-6}$ | Benign Very Strong | $5.31\times10^{-12}$ | Benign Very Strong | $4.43\times10^{-8}$ | Benign Very Strong | $1.96\times10^{-5}$ | Benign Very Strong | $5.81\times10^{-6}$ | Benign Very Strong |
| *BRCA2* | chr13_32890548_C_T | c.-39-11C>T | p.? | Uncertain | 72565 | 50782 | - | - | 1 ($1.97\times10^{-5}$) | 45 | 0.37 | Benign Supporting | 0.37 | Benign Supporting | 0.21 | Benign Moderate | 0.36 | Benign Supporting | 0.37 | Benign Supporting | 0.26 | Benign Supporting |
| *BRCA2* | chr13_32890547_INDEL_TCT | c.-39-12_-39-10del | p.? | Conflicting | 72563 | 50781 | 3 ($4.13\times10^{-5}$) | 58.3±9.5 (51-69) | 6 ($1.18\times10^{-4}$) | 54.7±9.5 (44-68) | $2.57\times10^{-4}$ | Benign Very Strong | $2.61\times10^{-4}$ | Benign Very Strong | $8.22\times10^{-7}$ | Benign Very Strong | $4.61\times10^{-6}$ | Benign Very Strong | $3.36\times10^{-4}$ | Benign Very Strong | $3.17\times10^{-4}$ | Benign Very Strong |
| *BRCA2* | chr13_32890555_G_T | c.-39-4G>T | p.? | Benign | 72564 | 50779 | 1 ($1.38\times10^{-5}$) | 65 | 2 ($3.94\times10^{-5}$) | 57±5.7 (53-61) | 0.04 | Benign Strong | 0.04 | Benign Strong | $5.33\times10^{-3}$ | Benign Strong | $5.20\times10^{-3}$ | Benign Strong | 0.06 | Benign Moderate | 0.05 | Benign Moderate |
| *BRCA2* | chr13_32899354_A_G | c.425+33A>G | p.? | (Likely) benign | 72417 | 50701 | 46 ($6.35\times10^{-4}$) | 58.7±9.6 (39-74) | 40 ($7.89\times10^{-4}$) | 54.6±11.9 (21-78) | $8.39\times10^{-20}$ | Benign Very Strong | $2.43\times10^{-20}$ | Benign Very Strong | $4.77\times10^{-37}$ | Benign Very Strong | $4.02\times10^{-30}$ | Benign Very Strong | $1.01\times10^{-17}$ | Benign Very Strong | - | - |
| *BRCA2* | chr13_32912868_A_G | c.4376A>G | p.(Asn1459Ser) | Conflicting | 72472 | 50739 | 1 ($1.38\times10^{-5}$) | 67 | - | - | 0.80 | No evidence | 0.80 | No evidence | 0.70 | No evidence | 0.71 | No evidence | 0.95 | No evidence | 0.85 | No evidence |
| *BRCA2* | chr13_32913148_C_T | c.4656T>C | p.(=) | Likely benign | 72558 | 50780 | 9 ($1.24\times10^{-4}$) | 54.3±11.4 (33-67) | 13 ($2.56\times10^{-4}$) | 52.2±8.1 (41-67) | $2.23\times10^{-7}$ | Benign Very Strong | $2.21\times10^{-7}$ | Benign Very Strong | $1.03\times10^{-12}$ | Benign Very Strong | $1.46\times10^{-10}$ | Benign Very Strong | $1.34\times10^{-6}$ | Benign Very Strong | $1.42\times10^{-7}$ | Benign Very Strong |
| *BRCA2* | chr13_32913173_C_A | c.4681C>A | p.(His1561Asn) | Conflicting | 72560 | 50782 | 3 ($4.13\times10^{-5}$) | 63.3±4.7 (58-67) | 1 ($1.97\times10^{-5}$) | 70 | 0.38 | Benign Supporting | 0.37 | Benign Supporting | 0.12 | Benign Moderate | 0.40 | Benign Supporting | 0.42 | Benign Supporting | 0.21 | Benign Moderate |
| *BRCA2* | chr13_32900313_C_T | c.475+26T>C | p.? | Likely benign | 72469 | 50740 | 7 ($9.66\times10^{-5}$) | 48.1±11.4 (33-61) | 2 ($3.94\times10^{-5}$) | 53.5±16.3 (42-65) | 4.87 | No evidence§ | 5.18 | No evidence§ | 0.69 | No evidence | 1.64 | No evidence | 3.72 | No evidence§ | 5.98 | No evidence§ |
| *BRCA2* | chr13_32900405_A_C | c.502C>A | p.(Pro168Thr) | Benign | 72558 | 50779 | 12 ($1.65\times10^{-4}$) | 57±11.4 (33-71) | 4 ($7.88\times10^{-5}$) | 56.8±12.7 (41-68) | $9.31\times10^{-3}$ | Benign Strong | $8.30\times10^{-3}$ | Benign Strong | $3.26\times10^{-5}$ | Benign Very Strong | $1.19\times10^{-4}$ | Benign Very Strong | 0.03 | Benign Strong | 0.05 | Benign Strong |
| *BRCA2* | chr13_32913910_A_G | c.5418A>G | p.(=) | Benign | 72543 | 50766 | 9 ($1.24\times10^{-4}$) | 55±9.5 (39-68) | 7 ($1.38\times10^{-4}$) | 59±11.1 (44-72) | $8.24\times10^{-4}$ | Benign Very Strong | $5.87\times10^{-4}$ | Benign Very Strong | $2.47\times10^{-7}$ | Benign Very Strong | $1.19\times10^{-5}$ | Benign Very Strong | $1.61\times10^{-3}$ | Benign Very Strong | $8.88\times10^{-4}$ | Benign Very Strong |
| *BRCA2* | chr13_32914127_A_G | c.5635G>A | p.(Glu1879Lys) | Conflicting | 72565 | 50780 | 14 ($1.93\times10^{-4}$) | 55.2±9.6 (38-66) | 10 ($1.97\times10^{-4}$) | 57.6±5.5 (48-67) | $4.11\times10^{-4}$ | Benign Very Strong | $3.57\times10^{-4}$ | Benign Very Strong | $3.40\times10^{-9}$ | Benign Very Strong | $7.85\times10^{-8}$ | Benign Very Strong | $1.91\times10^{-3}$ | Benign Very Strong | $1.86\times10^{-5}$ | Benign Very Strong |
| *BRCA2* | chr13_32900691_A_G | c.572A>G | p.(Asp191Gly) | Not Provided | 72563 | 50780 | 2 ($2.76\times10^{-5}$) | 54.5±31.8 (32-77) | - | - | 3.53 | No evidence§ | 3.72 | No evidence§ | 3.52 | No evidence§ | 3.72 | No evidence§ | 3.05 | No evidence§ | 3.25 | No evidence§ |
| *BRCA2* | chr13_32900694_C_T | c.575T>C | p.(Met192Thr) | Conflicting | 72555 | 50773 | 6 ($8.27\times10^{-5}$) | 59.3±6.4 (50-67) | - | - | 0.44 | Benign Supporting | 0.44 | Benign Supporting | 0.05 | Benign Moderate | 0.22 | Benign Moderate | 0.65 | No evidence | 0.97 | No evidence |
| *BRCA2* | chr13_32900706_A_G | c.587G>A | p.(Ser196Asn) | Uncertain | 72565 | 50779 | 1 ($1.38\times10^{-5}$) | 67 | 1 ($1.97\times10^{-5}$) | 38 | 0.26 | Benign Supporting | 0.26 | Benign Supporting | 0.17 | Benign Moderate | 0.22 | Benign Moderate | 0.33 | Benign Supporting | 0.29 | Benign Supporting |
| *BRCA2* | chr13_32914467_C_T | c.5975C>T | p.(Ser1992Leu) | Conflicting | 72560 | 50780 | 2 ($2.76\times10^{-5}$) | 60±9.9 (53-67) | 1 ($1.97\times10^{-5}$) | 45 | 0.32 | Benign Supporting | 0.36 | Benign Supporting | 0.12 | Benign Moderate | 0.25 | Benign Supporting | 0.35 | Benign Supporting | 0.26 | Benign Supporting |
| *BRCA2* | chr13_32914470_C_T | c.5978T>C | p.(Leu1993Ser) | - | 72547 | 50766 | 3 ($4.14\times10^{-5}$) | 62±17.3 (43-77) | 1 ($1.97\times10^{-5}$) | 55 | 0.30 | Benign Supporting | 0.27 | Benign Supporting | 0.07 | Benign Moderate | 0.10 | Benign Moderate | 0.56 | No evidence | 0.30 | Benign Supporting |
| *BRCA2* | chr13_32915240_A_G | c.6748A>G | p.(Thr2250Ala) | Benign | 72566 | 50779 | 17 ($2.34\times10^{-4}$) | 54.1±10.5 (31-71) | 8 ($1.58\times10^{-4}$) | 61.4±7.9 (48-70) | 0.14 | Benign Moderate | 0.12 | Benign Moderate | $3.24\times10^{-5}$ | Benign Very Strong | $3.15\times10^{-4}$ | Benign Very Strong | 0.10 | Benign Moderate | $1.89\times10^{-3}$ | Benign Very Strong |
| *BRCA2* | chr13_32903812_A_T | c.681+183T>A | p.? | - | 72485 | 50738 | 2 ($2.76\times10^{-5}$) | 59.5±10.6 (52-67) | - | - | 0.58 | No evidence | 0.60 | No evidence | 0.32 | Benign Supporting | 0.43 | Benign Supporting | 0.66 | No evidence | 1.00 | No evidence |
| *BRCA2* | chr13_32918701_A_C | c.6848C>A | p.(Pro2283His) | Uncertain | 72547 | 50769 | 3 ($4.14\times10^{-5}$) | 60±20 (40-80) | 1 ($1.97\times10^{-5}$) | 68 | 1.20 | No evidence | 1.12 | No evidence | 0.93 | No evidence | 0.17 | Benign Moderate | 1.72 | No evidence | 0.64 | No evidence |
| *BRCA2* | chr13_32918769_C_G | c.6916G>C | p.(Ala2306Pro) | Uncertain | 72312 | 50628 | 3 ($4.15\times10^{-5}$) | 54.3±16.5 (38-71) | 2 ($3.95\times10^{-5}$) | 66±5.7 (62-70) | 0.23 | Benign Supporting | 0.17 | Benign Moderate | 0.03 | Benign Strong | 0.08 | Benign Moderate | 0.16 | Benign Moderate | 0.29 | Benign Supporting |
| *BRCA2* | chr13_32918788_A_T | c.6935A>T | p.(Asp2312Val) | Benign | 72417 | 50682 | 6 ($8.29\times10^{-5}$) | 55.5±16.3 (32-72) | 2 ($3.95\times10^{-5}$) | 59.5±3.5 (57-62) | 0.24 | Benign Supporting | 0.22 | Benign Moderate | 0.02 | Benign Strong | 0.02 | Benign Strong | 0.37 | Benign Supporting | 0.36 | Benign Supporting |
| *BRCA2* | chr13_32919384_G_T | c.6937+594T>G | p.? | Benign | 72531 | 50758 | 42 ($5.79\times10^{-4}$) | 55.2±12.4 (30-79) | 16 ($3.15\times10^{-4}$) | 51.9±11.1 (37-71) | $3.35\times10^{-4}$ | Benign Very Strong | $2.88\times10^{-4}$ | Benign Very Strong | $2.25\times10^{-11}$ | Benign Very Strong | $1.03\times10^{-7}$ | Benign Very Strong | $3.82\times10^{-3}$ | Benign Strong | - | - |
| *BRCA2* | chr13_32921033_A_G | c.7007G>A | p.(Arg2336His) | Pathogenic | 72565 | 50781 | 4 ($5.51\times10^{-5}$) | 46±20.1 (28-74) | 3 ($5.91\times10^{-5}$) | 56±11.5 (47-69) | 1.13 | No evidence | 0.97 | No evidence | 0.54 | No evidence | 0.28 | Benign Supporting | 0.87 | No evidence | 0.51 | No evidence |
| *BRCA2* | chr13_32929309_A_G | c.7319A>G | p.(His2440Arg) | Benign | 72565 | 50781 | 8 ($1.10\times10^{-4}$) | 55.2±9.8 (39-68) | 6 ($1.18\times10^{-4}$) | 61.5±9.8 (46-72) | $3.69\times10^{-3}$ | Benign Strong | $3.10\times10^{-3}$ | Benign Strong | $3.66\times10^{-6}$ | Benign Very Strong | $5.62\times10^{-5}$ | Benign Very Strong | $5.61\times10^{-3}$ | Benign Strong | $3.44\times10^{-3}$ | Benign Strong |
| *BRCA2* | chr13_32930598_T_C | c.7469T>C | p.(Ile2490Thr) | Benign | 72560 | 50777 | 35 ($4.82\times10^{-4}$) | 50.2±10.6 (31-69) | 19 ($3.74\times10^{-4}$) | 55.3±12.7 (31-73) | $3.93\times10^{-3}$ | Benign Strong | $4.79\times10^{-3}$ | Benign Strong | $5.24\times10^{-11}$ | Benign Very Strong | $2.33\times10^{-5}$ | Benign Very Strong | $1.86\times10^{-3}$ | Benign Very Strong | $1.17\times10^{-4}$ | Benign Very Strong |
| *BRCA2* | chr13_32931973_A_C | c.7712A>C | p.(Glu2571Ala) | - | 72559 | 50779 | 2 ($2.76\times10^{-5}$) | 59.5±7.8 (54-65) | 3 ($5.91\times10^{-5}$) | 49±3.6 (46-53) | 0.01 | Benign Strong | 0.01 | Benign Strong | $6.34\times10^{-4}$ | Benign Very Strong | $3.50\times10^{-3}$ | Benign Strong | 0.02 | Benign Strong | 0.01 | Benign Strong |
| *BRCA2* | chr13_32936671_A_G | c.7817A>G | p.(Asp2606Gly) | - | 72554 | 50777 | 4 ($5.51\times10^{-5}$) | 64.5±13.7 (44-73) | - | - | 0.99 | No evidence | 0.90 | No evidence | 0.55 | No evidence | 0.57 | No evidence | 1.55 | No evidence | 1.07 | No evidence |
| *BRCA2* | chr13_32936680_A_G | c.7826G>A | p.(Gly2609Asp) | Likely pathogenic | 72563 | 50781 | 2 ($2.76\times10^{-5}$) | 51.5±0.7 (51-52) | 1 ($1.97\times10^{-5}$) | 56 | 0.20 | Benign Moderate | 0.23 | Benign Supporting | 0.03 | Benign Strong | 0.06 | Benign Strong | 0.24 | Benign Supporting | 0.29 | Benign Supporting |
| *BRCA2* | chr13_32936722_A_G | c.7868A>G | p.(His2623Arg) | (Likely) pathogenic | 72545 | 50765 | 5 ($6.89\times10^{-5}$) | 47.8±10.6 (35-64) | 2 ($3.94\times10^{-5}$) | 56.5±16.3 (45-68) | 0.88 | No evidence | 0.95 | No evidence | 0.10 | Benign Moderate | 0.27 | Benign Supporting | 0.85 | No evidence | 1.38 | No evidence |
| *BRCA2* | chr13_32936724_C_T | c.7870T>C | p.(Tyr2624His) | - | 72561 | 50779 | 3 ($4.13\times10^{-5}$) | 57.3±14.2 (41-67) | - | - | 2.56 | No evidence§ | 2.47 | No evidence§ | 3.06 | No evidence§ | 2.76 | No evidence§ | 3.59 | No evidence§ | 1.60 | No evidence |
| *BRCA2* | chr13_32936725_A_G | c.7871A>G | p.(Tyr2624Cys) | Uncertain | 72533 | 50769 | 3 ($4.14\times10^{-5}$) | 45.7±6.1 (39-51) | 2 ($3.94\times10^{-5}$) | 62.5±0.7 (62-63) | 1.10 | No evidence | 1.13 | No evidence | 0.28 | Benign Supporting | 0.16 | Benign Moderate | 0.69 | No evidence | 0.82 | No evidence |
| *BRCA2* | chr13_32936742_A_C | c.7888A>C | p.(Lys2630Gln) | Likely pathogenic | 72544 | 50775 | - | - | 3 ($5.91\times10^{-5}$) | 55.7±10.2 (44-63) | 0.01 | Benign Strong | 0.01 | Benign Strong | $3.40\times10^{-4}$ | Benign Very Strong | $1.00\times10^{-3}$ | Benign Very Strong | 0.01 | Benign Strong | 0.02 | Benign Strong |
| *BRCA2* | chr13_32936782_C_G | c.7928C>G | p.(Ala2643Gly) | Conflicting | 72409 | 50694 | 3 ($4.14\times10^{-5}$) | 53.3±7 (46-60) | 3 ($5.92\times10^{-5}$) | 58.7±6.7 (51-63) | 0.04 | Benign Strong | 0.05 | Benign Strong | $2.26\times10^{-3}$ | Benign Very Strong | $2.30\times10^{-3}$ | Benign Very Strong | 0.05 | Benign Moderate | 0.04 | Benign Strong |
| *BRCA2* | chr13_32937333_A_G | c.7994A>G | p.(Asp2665Gly) | Benign | 72555 | 50775 | 33 ($4.55\times10^{-4}$) | 59±11.6 (29-79) | 27 ($5.32\times10^{-4}$) | 50.8±14.4 (23-80) | $3.51\times10^{-13}$ | Benign Very Strong | $1.37\times10^{-13}$ | Benign Very Strong | $2.04\times10^{-23}$ | Benign Very Strong | $4.36\times10^{-20}$ | Benign Very Strong | $8.95\times10^{-12}$ | Benign Very Strong | - | - |
| *BRCA2* | chr13_32906415_A_G | c.800G>A | p.(Gly267Glu) | Conflicting | 72553 | 50778 | 7 ($9.65\times10^{-5}$) | 50±7.7 (39-63) | 2 ($3.94\times10^{-5}$) | 51±12.7 (42-60) | 3.09 | No evidence§ | 3.37 | No evidence§ | 0.18 | Benign Moderate | 1.17 | No evidence | 2.74 | No evidence§ | 3.20 | No evidence§ |
| *BRCA2* | chr13_32937396_C_T | c.8057T>C | p.(Leu2686Pro) | (Likely) pathogenic | 72547 | 50767 | 2 ($2.76\times10^{-5}$) | 58.5±7.8 (53-64) | 1 ($1.97\times10^{-5}$) | 68 | 0.17 | Benign Moderate | 0.17 | Benign Moderate | 0.04 | Benign Strong | 0.04 | Benign Strong | 0.19 | Benign Moderate | 0.28 | Benign Supporting |
| *BRCA2* | chr13_32937506_C_G | c.8167G>C | p.(Asp2723His) | Pathogenic | 72392 | 50680 | 18 ($2.49\times10^{-4}$) | 48.5±10.9 (27-74) | 1 ($1.97\times10^{-5}$) | 64 | 8193.33 | Pathogenic Very Strong | 9472.75 | Pathogenic Very Strong | 1784.63 | Pathogenic Very Strong | 3934.35 | Pathogenic Very Strong | 9412.08 | Pathogenic Very Strong | 17698.20 | Pathogenic Very Strong |
| *BRCA2* | chr13_32937582_A_G | c.8243G>A | p.(Gly2748Asp) | Pathogenic | 72565 | 50780 | 2 ($2.76\times10^{-5}$) | 45.5±2.1 (44-47) | - | - | 3.60 | No evidence§ | 3.28 | No evidence§ | 3.20 | No evidence§ | 3.82 | No evidence§ | 4.31 | No evidence§ | 4.30 | No evidence§ |
| *BRCA2* | chr13_32906446_T_G | c.831T>G | p.(Asn277Lys) | Conflicting | 72558 | 50776 | 20 ($2.76\times10^{-4}$) | 58±14.1 (33-78) | 12 ($2.36\times10^{-4}$) | 55.5±14.5 (22-71) | $6.16\times10^{-6}$ | Benign Very Strong | $3.75\times10^{-6}$ | Benign Very Strong | $9.75\times10^{-12}$ | Benign Very Strong | $1.08\times10^{-9}$ | Benign Very Strong | $1.15\times10^{-5}$ | Benign Very Strong | $1.94\times10^{-5}$ | Benign Very Strong |
| *BRCA2* | chr13_32944549_A_T | c.8342A>T | p.(Asn2781Ile) | Conflicting | 72384 | 50656 | 4 ($5.53\times10^{-5}$) | 61.5±9.4 (50-73) | 3 ($5.92\times10^{-5}$) | 60±10.8 (51-72) | $7.12\times10^{-3}$ | Benign Strong | $7.28\times10^{-3}$ | Benign Strong | $1.12\times10^{-4}$ | Benign Very Strong | $1.13\times10^{-3}$ | Benign Very Strong | 0.02 | Benign Strong | $8.76\times10^{-3}$ | Benign Strong |
| *BRCA2* | chr13_32944563_A_G | c.8356G>A | p.(Ala2786Thr) | Conflicting | 72565 | 50781 | 3 ($4.13\times10^{-5}$) | 49±18.3 (29-65) | 1 ($1.97\times10^{-5}$) | 52 | 1.19 | No evidence | 1.28 | No evidence | 1.31 | No evidence | 0.73 | No evidence | 1.22 | No evidence | 1.06 | No evidence |
| *BRCA2* | chr13_32944713_A_G | c.8487+19A>G | p.? | Benign | 72545 | 50773 | 13 ($1.79\times10^{-4}$) | 57.4±10.8 (33-71) | 12 ($2.36\times10^{-4}$) | 63.6±9.2 (43-75) | $1.59\times10^{-6}$ | Benign Very Strong | $6.48\times10^{-7}$ | Benign Very Strong | $3.32\times10^{-12}$ | Benign Very Strong | $2.95\times10^{-10}$ | Benign Very Strong | $1.89\times10^{-6}$ | Benign Very Strong | $4.60\times10^{-7}$ | Benign Very Strong |
| *BRCA2* | chr13_32953453_A_G | c.8755-1G>A | p.? | Pathogenic | 72562 | 50776 | 3 ($4.13\times10^{-5}$) | 31.7±1.2 (31-33) | - | - | 41.18 | Pathogenic Strong | 39.64 | Pathogenic Strong | 65.19 | Pathogenic Strong | 71.35 | Pathogenic Strong | 20.71 | Pathogenic Strong | 75.29 | Pathogenic Strong |
| *BRCA2* | chr13_32953473_A_G | c.8774A>G | p.(Gln2925Arg) | Uncertain | 72561 | 50781 | 2 ($2.76\times10^{-5}$) | 51.5±2.1 (50-53) | - | - | 1.53 | No evidence | 1.61 | No evidence | 0.62 | No evidence | 1.25 | No evidence | 1.38 | No evidence | 1.41 | No evidence |
| *BRCA2* | chr13_32953529_A_T | c.8830A>T | p.(Ile2944Phe) | Benign | 72510 | 50745 | 9 ($1.24\times10^{-4}$) | 50.4±8.8 (39-66) | 9 ($1.77\times10^{-4}$) | 52.6±7 (38-59) | $3.89\times10^{-4}$ | Benign Very Strong | $8.80\times10^{-4}$ | Benign Very Strong | $2.39\times10^{-8}$ | Benign Very Strong | $1.11\times10^{-6}$ | Benign Very Strong | $3.57\times10^{-3}$ | Benign Strong | $1.22\times10^{-4}$ | Benign Very Strong |
| *BRCA2* | chr13_32954180_C_T | c.9154C>T | p.(Arg3052Trp) | Pathogenic | 72563 | 50779 | 10 ($1.38\times10^{-4}$) | 49.1±15.6 (30-78) | 1 ($1.97\times10^{-5}$) | 56 | 86.82 | Pathogenic Strong | 104.46 | Pathogenic Strong | 20.23 | Pathogenic Strong | 42.62 | Pathogenic Strong | 84.20 | Pathogenic Strong | 117.18 | Pathogenic Strong |
| *BRCA2* | chr13_32954340_A_T | c.9256+58A>T | p.? | Likely benign | 72345 | 50651 | 24 ($3.32\times10^{-4}$) | 53.9±12.2 (27-75) | 14 ($2.76\times10^{-4}$) | 52.6±10.5 (31-66) | $8.70\times10^{-7}$ | Benign Very Strong | $1.03\times10^{-6}$ | Benign Very Strong | $5.01\times10^{-13}$ | Benign Very Strong | $2.52\times10^{-11}$ | Benign Very Strong | $4.90\times10^{-6}$ | Benign Very Strong | $8.17\times10^{-6}$ | Benign Very Strong |
| *BRCA2* | chr13_32968940_A_T | c.9371A>T | p.(Asn3124Ile) | Pathogenic | 72548 | 50755 | 16 ($2.21\times10^{-4}$) | 50.3±10.5 (38-74) | - | - | 3530.99 | Pathogenic Very Strong | 4197.75 | Pathogenic Very Strong | 1337.24 | Pathogenic Very Strong | 4557.92 | Pathogenic Very Strong | 5079.21 | Pathogenic Very Strong | 4957.08 | Pathogenic Very Strong |
| *BRCA2* | chr13_32969073_A_T | c.9501+3A>T | p.? | Benign | 72442 | 50723 | 25 ($3.45\times10^{-4}$) | 53.6±11.1 (32-76) | 22 ($4.34\times10^{-4}$) | 57.6±10.8 (32-73) | $6.12\times10^{-9}$ | Benign Very Strong | $5.06\times10^{-9}$ | Benign Very Strong | $2.30\times10^{-18}$ | Benign Very Strong | $8.76\times10^{-15}$ | Benign Very Strong | $4.12\times10^{-8}$ | Benign Very Strong | $6.08\times10^{-10}$ | Benign Very Strong |
| *BRCA2* | chr13_32969079_A_C | c.9501+9A>C | p.? | Benign | 72550 | 50761 | 25 ($3.45\times10^{-4}$) | 58.4±10.5 (38-76) | 13 ($2.56\times10^{-4}$) | 49.1±14.1 (25-74) | $4.01\times10^{-7}$ | Benign Very Strong | $1.70\times10^{-7}$ | Benign Very Strong | $1.98\times10^{-13}$ | Benign Very Strong | $1.99\times10^{-9}$ | Benign Very Strong | $1.09\times10^{-5}$ | Benign Very Strong | $1.17\times10^{-6}$ | Benign Very Strong |
| *BRCA2* | chr13_32971023_C_T | c.9502-12T>C | p.? | (Likely) benign | 72494 | 50742 | 30 ($4.14\times10^{-4}$) | 56.2±11.9 (26-78) | 30 ($5.91\times10^{-4}$) | 52±8.5 (29-74) | $3.54\times10^{-16}$ | Benign Very Strong | $5.06\times10^{-16}$ | Benign Very Strong | $1.89\times10^{-30}$ | Benign Very Strong | $4.18\times10^{-24}$ | Benign Very Strong | $2.49\times10^{-14}$ | Benign Very Strong | - | - |
| *BRCA2* | chr13_32971042_A_G | c.9509A>G | p.(Asp3170Gly) | Benign | 72523 | 50748 | 6 ($8.27\times10^{-5}$) | 48.7±14 (32-65) | 3 ($5.91\times10^{-5}$) | 52±21.7 (29-72) | 0.78 | No evidence | 0.71 | No evidence | 0.06 | Benign Moderate | 0.55 | No evidence | 0.87 | No evidence | 0.71 | No evidence |
| *BRCA2* | chr13_32971125_C_T | c.9592T>C | p.(Cys3198Arg) | Benign | 72506 | 50747 | 12 ($1.66\times10^{-4}$) | 53.8±12 (37-78) | 6 ($1.18\times10^{-4}$) | 54.5±10.8 (38-68) | 0.05 | Benign Moderate | 0.05 | Benign Strong | $5.98\times10^{-5}$ | Benign Very Strong | $5.61\times10^{-4}$ | Benign Very Strong | 0.06 | Benign Moderate | 0.03 | Benign Strong |
| *BRCA2* | chr13_32972279_C_T | c.9649-20C>T | p.? | Benign | 72559 | 50780 | 13 ($1.79\times10^{-4}$) | 55.3±13.2 (34-75) | 9 ($1.77\times10^{-4}$) | 55.4±6.7 (47-65) | $2.43\times10^{-4}$ | Benign Very Strong | $1.61\times10^{-4}$ | Benign Very Strong | $3.85\times10^{-8}$ | Benign Very Strong | $3.43\times10^{-7}$ | Benign Very Strong | $1.21\times10^{-3}$ | Benign Very Strong | $2.20\times10^{-4}$ | Benign Very Strong |
| *BRCA2* | chr13_32906579_A_C | c.964A>C | p.(Lys322Gln) | Conflicting | 72483 | 50722 | 2 ($2.76\times10^{-5}$) | 56.5±13.4 (47-66) | 1 ($1.97\times10^{-5}$) | 67 | 0.50 | No evidence | 0.46 | Benign Supporting | 0.19 | Benign Moderate | 0.15 | Benign Moderate | 0.58 | No evidence | 0.49 | No evidence |

† Age: mean±sd (range), ‡Aligned ACMG/AMP evidence strengths are modified following cut-offs resulted from the analysis of simulated datasets; Moderate and Supporting evidence strengths in favor of pathogenicity are noted as "No evidence§"

LR, Likelihood ratio; ACMG/AMP, American College of Medical Genetics and Genomics (ACMG) and Association for Molecular Pathology (AMP); NA, LR value not available due to overflowing. Variant nomenclature according to: *BRCA1* (NM_007294.4, NP_009225.1), *BRCA2* (NM_000059.3, NP_000050.2).
