# Supplementary figures and images for: A Likelihood Ratio Approach for Utilizing Case-Control Data in the Clinical Classification of Rare Sequence Variants: Application to BRCA1 and BRCA2
Source: Hum Mutat. 2023 Sep 14;2023:9961341. doi: 10.1155/2023/9961341 (PMC11080979; doi:10.1155/2023/9961341)

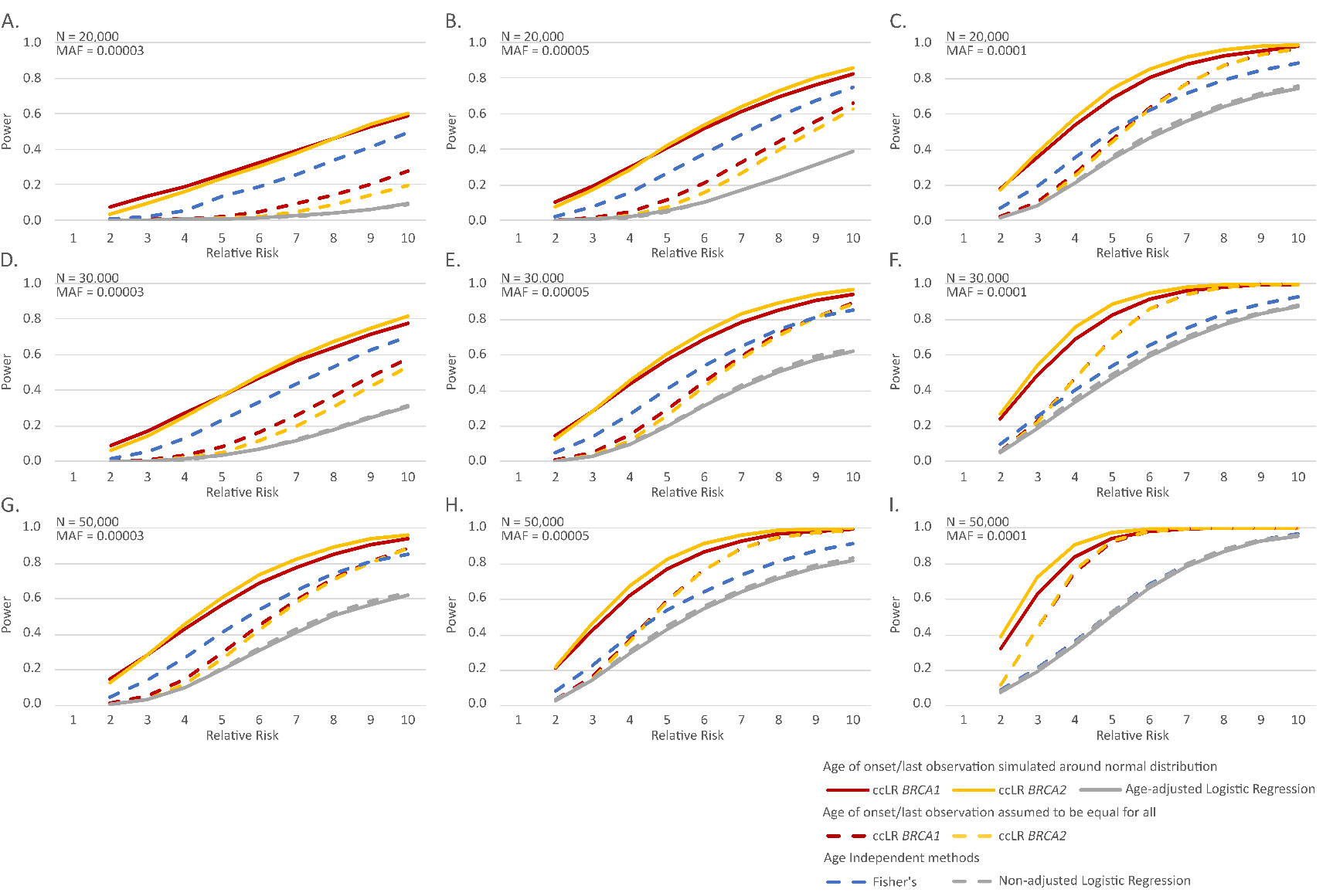


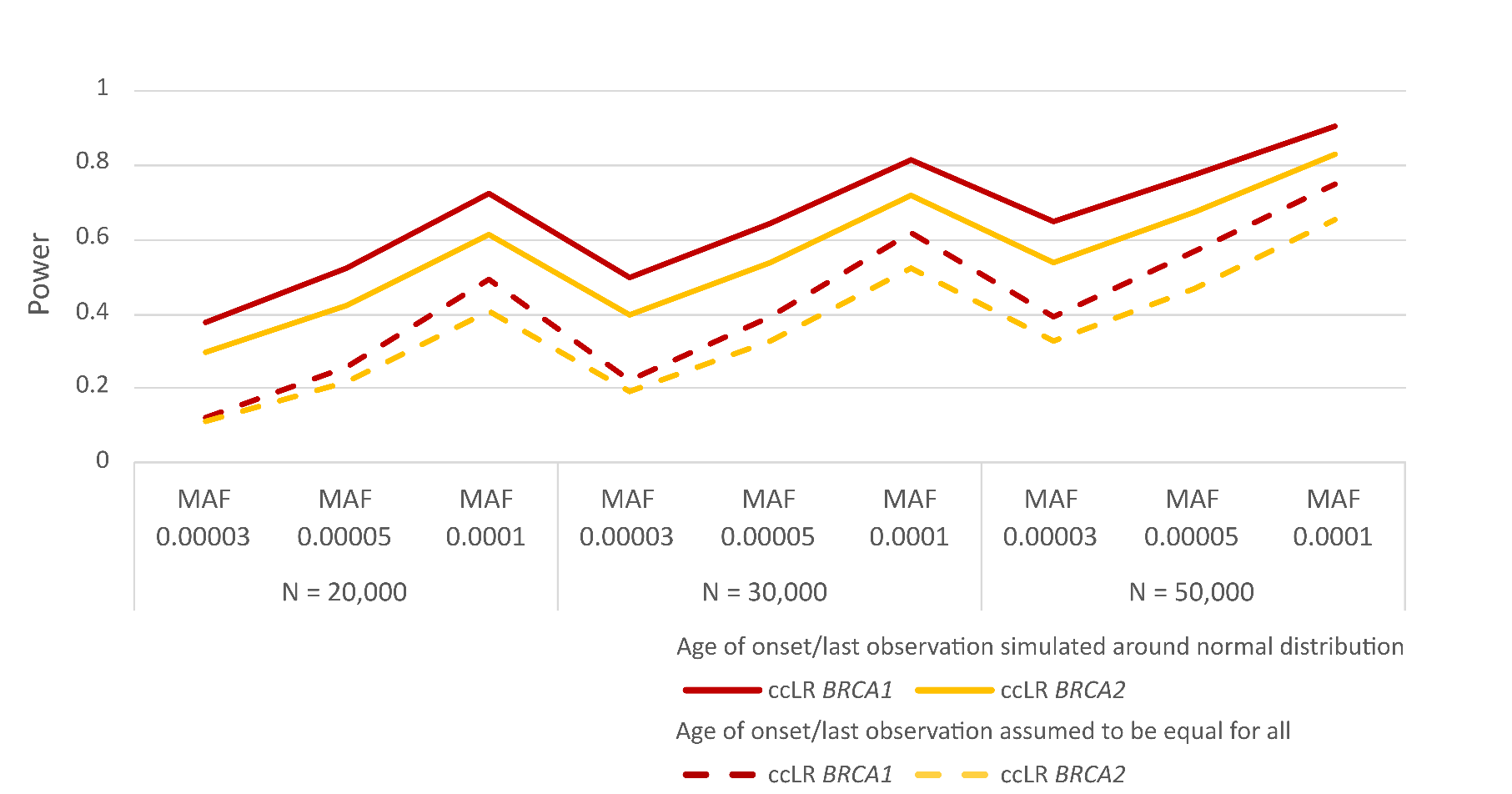

Supplement: Supplementary 4 — Supplementary Figure S1: Performance of the case-control likelihood ratio method and odds ratio analysis in providing at least strong ACMG/AMP evidence in favor of pathogenicity (LR ≥ 18.7), using simulated datasets of assumed same age. Power equals the probability of reaching at least strong pathogenic ACMG/AMP evidence. Genotype data simulations were carried out for causal variants conferring disease relative risk between 2 and 10. We performed 10,000 simulations for each case scenario. Results represent simulated case-control data for 20,000 (A–C), or 30,000 (D–F) or 50,000 (G–I) breast cancer cases and controls, and minor allele frequency of 0.00003 (A–G), 0.00005 (B–H), or 0.0001 (C–I). ccLR: case-control likelihood ratio; MAF: minor allele frequency; N: sample size. Supplementary Figure S2: performance of the case-control likelihood ratio method in providing ACMG/AMP evidence against pathogenicity using simulated datasets of assumed same age. Power equals the probability of reaching at least supporting benign ACMG/AMP evidence (LR ≤ 0.48) when the relative risk was set to 1. We performed 10,000 simulations for each case scenario. Results represent simulated case-control data for 20,000, 30,000, or 50,000 breast cancer cases and controls and minor allele frequency of 0.00003, 0.00005, or 0.0001. ccLR: case-control likelihood ratio; MAF: minor allele frequency; N: sample size. [file 9961341.f4.docx]
